# Isolation and anti-neuroinflammation activity of sesquiterpenoids from *Artemisia argyi*: computational simulation and experimental verification

**DOI:** 10.1186/s12906-024-04578-z

**Published:** 2024-07-11

**Authors:** Caiwenjie La, Menghe Li, Zexu Wang, Tao Liu, Qiongzhen Zeng, Pinghua Sun, Zhe Ren, Cuifang Ye, Qiuying Liu, Yifei Wang

**Affiliations:** 1 Department of Cell Biology, College of Life Science and Technology, Jinan University, Guangzhou, China; 2 Guangdong Provincial Biotechnology Drug & Engineering Technology Research Center, Guangzhou, China; 3 The Second Clinical Medical College, The First Affiliated Hospital, Shenzhen People’s Hospital, Jinan University, Southern University of Science and Technology, Shenzhen, 518020 China; 4 College of Pharmacy, Jinan University, Guangzhou, 510632 China

**Keywords:** *Artemisia argyi*, Natural products, Sesquiterpenoids, Neuroinflammation, Molecular dynamics simulation

## Abstract

**Background:**

*Artemisia argyi* is a traditional herbal medicine belonging to the genus *Artemisia* that plays an important role in suppressing inflammation. However, the chemical constituents and underlying mechanisms of its therapeutic potential in neuroinflammation are still incompletely understood, and warrant further investigation.

**Methods:**

Several column chromatography were employed to isolate and purify chemical constituents from *Artemisia argyi*, and modern spectroscopy techniques were used to elucidate their chemical structures. The screening of monomeric compounds with nitric oxide inhibition led to the identification of the most effective bioactive compound, which was subsequently confirmed for its anti-inflammatory capability through qRT‒PCR. Predictions of compound-target interactions were made using the PharmMapper webserver and the TargetNet database, and an integrative protein-protein interaction network was constructed by intersecting the predicted targets with neuroinflammation-related targets. Topological analysis was performed to identify core targets, and molecular docking and molecular dynamics simulations were utilized to validate the findings. The result of the molecular simulations was experimentally validated through drug affinity responsive target stability (DARTS) and Western blot experiments.

**Results:**

Seventeen sesquiterpenoids, including fifteen known sesquiterpenoids and two newly discovered guaiane-type sesquiterpenoids (argyinolide S and argyinolide T) were isolated from *Artemisia argyi*. Bioactivity screening revealed that argyinolide S (AS) possessed the most potent anti-inflammatory activity. However, argyinolide T (AT) showed weak anti-inflammatory activity, so AS was the target compound for further study. AS may regulate neuroinflammation through its modulation of eleven core targets: protein kinase B 1 (AKT1), epidermal growth factor receptor (EGFR), proto-oncogene tyrosine-protein Kinase (FYN), Janus Kinase (JAK) 1, mitogen-activated protein (MAP) Kinase 1,8 and 14, matrix metalloproteinase 9 (MMP9), ras-related C3 botulinum toxin substrate 1 (RAC1), nuclear factor kappa-B p65 (RELA), and retinoid X receptor alpha (RXRA). Molecular dynamics simulations and DARTS experiments confirmed the stable binding of AS to JAK1, and Western blot experiments demonstrated the ability of AS to inhibit the phosphorylation of downstream Signal transducer and activator of transcription 3 (STAT3) mediated by JAK1.

**Conclusions:**

The sesquiterpenoid compounds isolated from *Artemisia argyi*, exhibit significant inhibitory effects on inflammation in C57BL/6 murine microglia cells (BV-2). Among these compounds, AS, a newly discovered guaiane-type sesquiterpenoid in *Artemisia argyi*, has been demonstrated to effectively inhibit the occurrence of neuroinflammation by targeting JAK1.

**Supplementary Information:**

The online version contains supplementary material available at 10.1186/s12906-024-04578-z.

## Introduction

Traumatic injury to the central nervous system results in blood-brain barrier impairment and neuroglial cell activation, leading to the onset of neuroinflammation [1]. Neuroinflammation is an inflammatory state that occurs within the central nervous system (CNS), and serves as a shared pathological factor in several neurodegenerative diseases, such as Alzheimer’s disease, Parkinson’s disease, Huntington’s disease, and multiple sclerosis. Multiple cell types, including microglia, astrocytes, and macrophages, actively participate in the immune response associated with neuroinflammation [2]. Microglia, as crucial immune effector cells within the central nervous system, play essential roles in the onset and development of neuroinflammation [3, 4]. Microglia rapidly respond to injury by activating an inflammatory response, functioning as phagocytes to eliminate bacteria, dead cells, abnormal protein aggregates, and other potentially harmful substances that may jeopardize the central nervous system. Nevertheless, excessive activation of microglia can result in central nervous system disorders with systemic implications. Consequently, alleviating neurological conditions arising from neuroinflammation is achievable through the overall suppression of the inflammatory response in microglia [5–7].

Secondary metabolites derived from therapeutic plants can interact with various signaling pathways and targets, thereby mitigating the damage caused by neuroinflammation. The neuroprotective effects of naturally occurring flavonoids, fatty acids, sesquiterpene lactones, polyphenols, and their derivatives have been extensively studied due to their antioxidant and anti-inflammatory properties in the context of neurodegenerative diseases [8–10]. Various sesquiterpenoids derived from *Artemisia* plants have been shown to inhibit neuroinflammation through multiple signaling pathways, including nuclear factor kappa-B (NF-κB), c-JunN-terminal Kinase/p38 (JNK) and mitogen-activated protein Kinase (MAPK) [11, 12].

*Artemisia argyi* is a significant species within the *Artemisia* genus, with a medicinal history of more than 2,000 years. More than 200 chemical components, including terpenoids, flavonoids, polyphenols and other compounds, have been identified in *Artemisia argyi* leaves [13]. Therefore, *Artemisia argyi* leaves have rich biological activities and pharmacological effects, including antioxidant, anti-inflammatory and antitumor effects [14–17]. Previously, we discovered that the essential oil of *Artemisia argyi* suppresses the polymerization of NOD-like receptor thermal protein domain associated protein 3 (NLRP3) and exhibits anti-inflammatory effects [18], while the sesquiterpenoids from *Artemisia vulgaris* and *Artemisia argyi* demonstrate excellent anti-inflammatory activity in mouse RAW264.7 macrophages (RAW264.7) [19–21]. Based on prior research findings and pertinent literature, our hypothesis is that the sesquiterpenoids derived from this plant possess remarkable therapeutic efficacy against neuroinflammation. We extracted 17 sesquiterpenoids from *Artemisia argyi* leaves, including 15 known sesquiterpenoids and 2 new guaiane-type sesquiterpenoids named argyinolide S and argyinolide T. Using C57BL/6 murine microglia (BV-2) that were activated by lipopolysaccharide (LPS), we evaluated their ability to mitigate microglial inflammatory responses. The results indicate that among the compounds we tested, six sesquiterpenoids exhibit superior anti-inflammatory effects compared to dexamethasone, especially AS. Subsequently, bioinformatics approaches were employed to investigate the underlying mechanisms and potential targets responsible for the neuroinflammation-inhibiting effects of AS, and the computational results were confirmed by DARTS experiments.

## Materials and methods

### Plant material

Fresh *Artemisia argyi* leaves were collected from Nanyang, Henan Province, China, in May 2019. The leaves were identified by Professor Yifei Wang, and the voucher specimen (No. 201,905) was deposited at the Guangzhou Jinan Biomedicine Research and Development Center.

### Extraction and isolation

As previously described [21], the initial extraction process of *Artemisia argyi* leaves was as follows. After being collected from their cultivation site, *Artemisia argyi* leaves were dried and placed in a well-ventilated area at room temperature for three months. Subsequently, 200 kg of dried *Artemisia argyi* leaves were pulverized, followed by three extractions using 2000 L of 95% ethanol (Energy-chemical, Anhui China, W310136). The concentration of the extracts yielded 14.3 kg of final extract. This extract was further divided into petroleum ether (PE) and ethyl acetate (EA) extracts, with weights of 2.42 kg and 2.41 kg respectively. This extract was further divided into petroleum ether and ethyl acetate extracts, with weights of 2.42 kg and 2.41 kg respectively. The ethyl acetate fraction was subjected to silica gel (Qingdao Marine Chemical Ltd., Qingdao, China) column chromatography (PE: EA, 5:1–1:1), yielding Fr. 1–7. The petroleum ether extract was sonicated using 80% CH_3_OH-H_2_O, resulting in a CH_3_OH-H_2_O extract weighing 440 g. This extract was then subjected to macroporous adsorption resin (type: AB-8) column chromatography (ethanol: H_2_O, 50-100%), yielding Fr. A-D. Repeated chromatography was used to separate these fractions, including silica gel (Qingdao Marine Chemical Ltd., Qingdao, China), MCI gel (SaiPuRuiSi, Beijing, China), YMC ODS-A-HG gel (50 μm, YMC, Japan), and Sephadex LH-20 gel(GE Healthcare, Sweden). Seventeen monomers were separated by preparative high-performance liquid chromatography (HPLC) using an Agilent 1260 system with a YSC-PACK ODS-A column (250 × 10 mm, 5 μm).

### Structural identification and absolute configuration analysis

Nuclear magnetic resonance (NMR) spectra of ^1^H (600 MHz) and ^13^C (150 MHz) were obtained with deuterium reagent on a Bruker AVANCE-600 instrument, and the planar structures of the compounds were characterized. The relative configurations of the compounds were determined by the nuclear Overhauser effect spectroscopy (NOESY). Circular dichroism (CD) was measured by a Chirascan Plus spectrometer (Applied Photophysics, UK), and the absolute configurations were determined based on electron circular dichroism fitting and DP4 + NMR calculations. High-resolution electrospray ionization mass spectrometry (HRESIMS) spectra were acquired using an ALA Sciex Triple-TOF 5600 + apparatus. The CD and NMR calculation data were generated using Gaussian 09 software. For the CD calculations, a procedure outlined in a previous study [22] was followed, where a random conformational search was conducted under the MMFF force field. The optimized stable conformations were further subjected to B3LYP/6-31G (d) level optimization, after screening based on the Boltzmann distribution. Subsequently, the selected optimized conformations were utilized for further ECD calculations at the B3LYP/6-311G (d, p) level in the IEFPCM model. The contributions of each conformational isomer, as calculated by the Boltzmann distribution, were used to weight the overall ECD data after UV correction. The ECD curves and enantiomeric ECD curves were generated using SpecDis 1.70.1 software. GIAO MPW1PW91/6–31 + G(d, p) level calculations were employed to compute the NMR data while considering solvent effects.

### Cell culture and treatment

BV-2 cells were obtained from the Shanghai Cell Bank of the Chinese Academy of Sciences. The cells were grown in Dulbecco’s modified Eagle’s medium (DMEM), 10% fetal bovine serum (#10100-147, Gibco), and 1% penicillin‒streptomycin (#15,070,063, Gibco) at 37 °C in a humid environment with 5% CO_2_.

### Cytotoxic activity

The cellular toxicity of the compounds on BV-2 cells was determined using a CCK-8 assay kit. BV-2 cells were seeded at a density of 2 × 10^4^ cells per well in DMEM supplemented with 10% FBS and cultured overnight. The cells were then exposed to various doses of AS for 24 h. 10 µL of cell counting kit-8 (CCK-8) solution was added to each well after treatment, and each well was then incubated at 37 °C in the dark for 30 min. An enzyme-linked immunosorbent assay reader (Bio-Rad Laboratories, Inc., Hercules, CA, USA) was used to measure the absorbance at 450 nm. The viability of control cells not treated with AS was compared to the cell viability at each concentration. The experiment was carried out three times.

### Inhibition of NO production

The production of nitric oxide (NO) was indirectly measured using a Griess reagent kit (Beyotime, Shanghai, China). BV-2 cells were seeded in a 96-well plate at a density of 5 × 10^4^ cells per well and cultured for 18 h. The cells were treated with LPS (1 µg/mL) alone or in combination for a total of 18 h. 50 µL of the supernatant from each well was collected before the addition of equal volumes of Griess reagent A and B. The plate was then allowed to stand at room temperature for 10 min, after which the absorbance was measured at 540 nm using an enzyme-linked immunosorbent assay reader (Bio-Rad Laboratories, Inc., Hercules, CA, USA). The percentage of NO inhibition was calculated based on the absorbance values of the model group and the blank control group. The experiment was performed in triplicate.

### Identification of AS Neuroinflammation associated genes

Information regarding the targets of neuroinflammation was obtained from the GeneCards database [23] and the DisGeNET database [24]. The structural information of compound AS was uploaded to the PharmMapper webserver [25] and the TargetNet database [26] to predict potential target interactions. A set of composite targets was produced as a result of the intersection of the targets for AS and the targets for neuroinflammation.

### Construction of the PPI network and selection of key targets

The STRING database was used for further analysis of overlapping target proteins [27]. A network map of protein‒protein interactions (PPIs) was created using an interaction score with a high level of confidence (0.7). The cytoHubba plugin [28] in Cytoscape 3.9.0 was used to calculate the MCC, DMNC, MNC, and degree values. The key targets were those whose MCC, DMNC, MNC, and degree scores were higher than average.

### Molecular docking

The target protein structure was downloaded in PDB format from the RCSB database. The PDB IDs for each target are as follows: JAK1 (3EYG), FYN (2DQ7), RXRA (1FM9), MAPK1 (4FV9), MAPK8 (1UKI), EGFR (1XKK), MMP9 (4WZV), AKT1 (3QKL), MAPK14 (1A9U), RELA (6YOY), and RAC1 (1E96). Preprocessing of the proteins was performed using Autodock. The ligand structure was drawn in ChemDraw, and its three-dimensional conformation was obtained through energy minimization in Chem3D. Subsequently, the ligand was processed in the Autodock software package, where the Detect Root and Choose Torsions commands were executed, resulting in the export of pdbqt files. The docking box information was extracted from PyMOL using the getbox plugin, and utilized by the Vina main program for the docking process [29, 30]. PyMOL [31] and Discovery Studio were used to visualize the docking results.

### Molecular dynamics simulation

The initial conformation obtained from molecular docking was used as the starting structure to generate the topology and coordinate files of the receptor using the AMBER14SB force field. The input file of the ligand was generated under the gaff force field using AmberTools22 and then converted format using the acpype program [32, 33]. The gromacs software suite was used to run molecular dynamics simulations [34]. The TIP3P water model was used for solvation during the simulations. To stabilize the system temperature at 310 K, the prepared systems underwent an energy minimization step, followed by 100 ps NVT equilibration. The system pressure was then balanced at 1 bar by means of 100 ps NPT equilibration. A 50 ns molecular dynamics simulation was run after the equilibration stages.

### Molecular mechanic/Poisson-Boltzmann surface area (MM-PBSA) calculation

The relative binding energies of the protein-ligand complexes were calculated using the gmx_mmpbsa tool based on trajectory data obtained from molecular dynamics simulations [35]. Based on the stability throughout the simulation process, trajectories recorded between 40 and 50 ns were used for the calculation of the binding free energy.

### qRT‒PCR

BV-2 cells were seeded at a density of 5 × 10^5^ cells per well in a 6-well plate and cultured for 18 h. LPS (1 µg/mL) was then administered to the cells alone or in combination with AS for 6 h. Total RNA was isolated from cellular samples using TRIzol reagent, after which the RNA concentration was determined using the PrimeScript RT Reagent Kit. Subsequently, the extracted RNA underwent reverse transcription to generate complementary DNA (cDNA). qRT‒PCR assays were conducted utilizing the CFX96 Touch Real-Time PCR Detection System. Relative gene expression levels were normalized to the β-actin internal housekeeping gene. All reported data are representative of a minimum of three independent experiments, and the gene-specific primers used are listed in Table S1.

### 13 Western blot

BV-2 cells were seeded at a density of 5 × 10^5^ cells per well in a 6-well plate and cultured for 6 h. Following treatment with LPS (1 µg/mL) alone or in combination with AS for a total of 6 h, BV-2 cells were harvested and lysed using RIPA lysis buffer containing a mixture of protease and phosphatase inhibitors to obtain lysates. Total protein was then measured using a bicinchoninic acid (BCA) protein assay kit, and adjustments were made using sample buffer and RIPA lysis buffer. The samples were separated by sodium dodecyl sulfate -polyacrylamide gel electrophoresis (SDS-PAGE), transferred onto polyvinylidene fluoride membranes (Millipore), and subsequently blocked with 5% skim milk. The membranes were incubated with primary antibodies overnight at 4 °C, followed by incubation with specific secondary antibodies. Finally, protein bands were detected using ImageJ software. Information on the antibodies used can be found in Table S2.

### DARTS

The DARTS experiment was conducted following guidelines [36]. BV-2 cells were seeded in cell culture dishes and cultured until they reached 85-90% confluence. Total protein was extracted using NP40 lysis buffer containing protease and phosphatase inhibitors. The concentration of total protein was determined using a BCA protein assay kit. The total protein was then incubated with different concentrations of AS at 4 °C overnight. The following day, 0.05% pronase was added and the samples were incubated at room temperature for 20 min. Subsequently, 5× loading buffer was added, and the mixture was boiled at 100 °C for 10 min to terminate the reaction. The expression of the target protein was detected via Western blotting, with the pronase negative control group utilized as the negative control.

## Results

### Structural identification of compounds

**Fig. 1:**
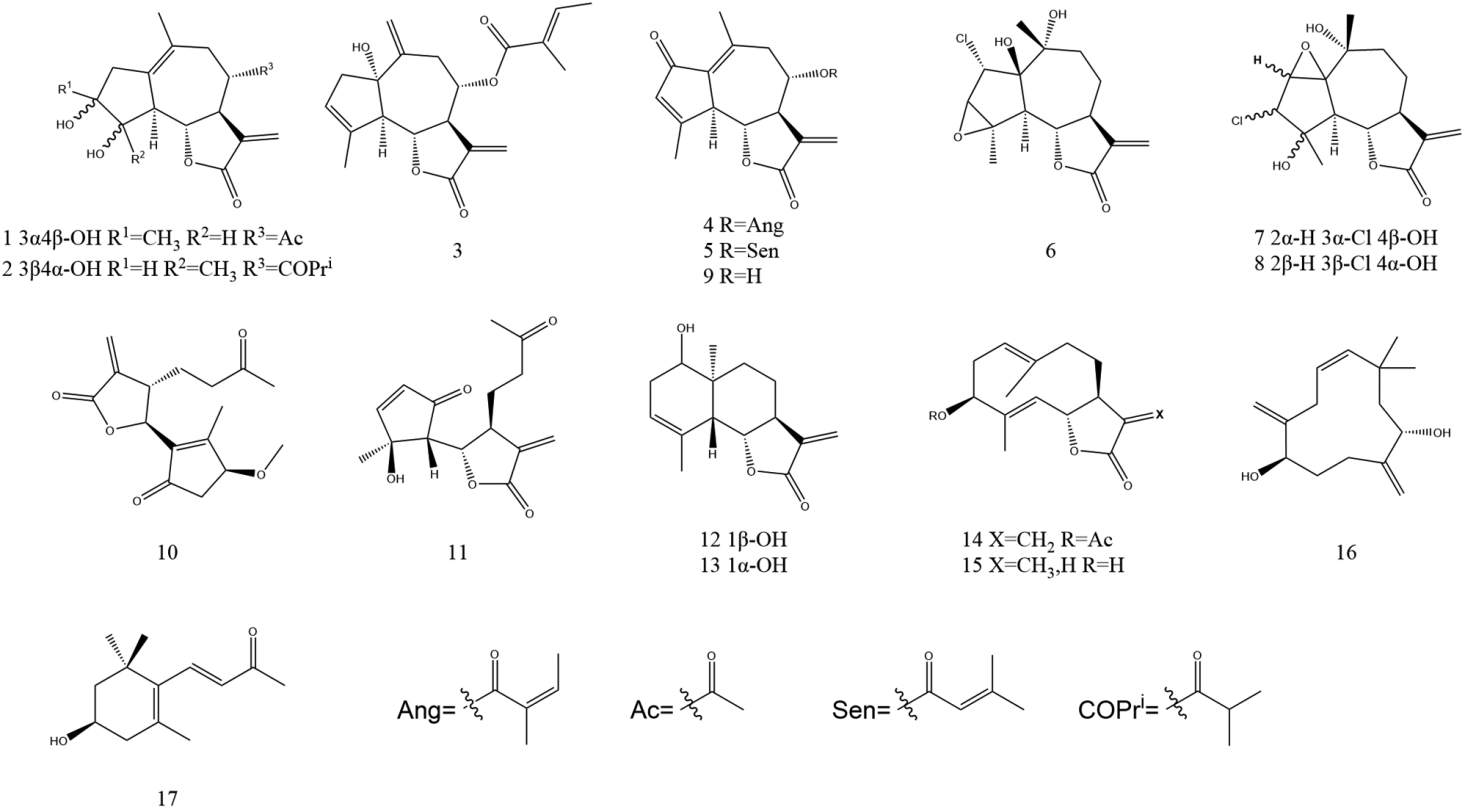
Structures of Compounds **1–17**

Two previously unreported guaiane-type sesquiterpenoids (**1–2**), along with a series of known compounds (**3–17**), were isolated from the 95% ethanol extract of *Artemisia argyi* (Fig. 1). The details are shown in Table 1. The structures of these compounds were elucidated using various techniques, including 1D-NMR, 2D-NMR, HRESIMS, DP4 + analysis, and ECD calculations.

**Table 1:** Compound information

No	Name	Molecular formula	Properties
1	argyinolide S	C_17_H_22_O_6_	white powder
2	argyinolide T	C_19_H_26_O_6_	white needle crystal
3	artemvulactone N	C_20_H_24_O_5_	colorless oil
4	moxartenolide	C_20_H_22_O_5_	white powder
5	Artemisiane E	C_20_H_22_O_5_	white powder
6	artemvulactone S	C_15_H_19_O_5_Cl	white powder
7	3*α*-Chloro-4*β*,10*α*-dihydroxy-1*β*,2*β*-epoxy-5*α*,7*α*H-guai-11(13)-en-12,6*α*-olide	C_15_H_19_O_5_Cl	colorless gum
8	3*β*-Chloro-4*α*,10*α*-dihydroxy-1*α*,2*α*-epoxy-5*α*,7*α*H-guaia-11(13)-en-12,6*α*-olide	C_15_H_19_O_5_Cl	colorless gum
9	11,13-dehydro-desacetylmatricarin	C_15_H_16_O_4_	white powder
10	3-*β*-methoxy-iso-seco-tanapartholide	C_16_H_20_O_5_	colorless oil
11	(4S*,5S*)-dihydro-5-[(1R*,2S*)-2-hydroxy-2-methyl-5-oxo-3-cyclopenten-1-yl]-3-methylene-4-(3-oxobutyl)-2(3*H*)-furanone	C_15_H_18_O_5_	colorless gum
12	douglanin	C_15_H_20_O_3_	white powder
13	santamarine	C_15_H_20_O_3_	white powder
14	(3a*S*,6*E*,9*S*,10*E*,11a*R*)-9-(Acetyloxy)-3a,4,5,8,9,11a-hexahydro-6,10-dimethyl-3-methylenecyclodeca[*b*]furan-2(3*H*)-one	C_17_H_22_O_4_	white powder
15	Cyclodeca[b]furan-2(3 H)-one, 3a,4,5,8,9,11a-hexahydro-9-hydroxy-3,6,10-trimethyl-, (3*S*,3a*S*,6*E*,9*S*,10*E*,11a*S*)- (ACI)	C_15_H_20_O_3_	colorless gum
16	*rel*-(1*R*,5*S*,8*E*)-10,10-Dimethyl-2,6-bis(methylene)-8-cycloundecene-1,5-diol	C_15_H_24_O_2_	white powder
17	(+)-3-hydroxy-β-ionone	C_13_H_20_O_2_	white powder

**Table 2:** ^1^H NMR (600 MHz) and ^13^C NMR (150 MHz) data (*δ*) for compound **1** in CDCl_3_ (*δ* in ppm, *J* in hz)

NO	1	
	*δ*_H_ (*J* in Hz)	*δ*_C_
1	-	133.86
2	3.05, m 2.36, d (17.1)	39.83
3	-	80.22
4	4.19, t (10.0)	79.79
5	3.0, d (10.34)	54.48
6	4.16, d (4.6)	80.15
7	3.12, tt (10.3, 3.1)	52.55
8	4.94, m	70.89
9	2.42, d (6.1)	42.10
10	-	127.00
11	-	136.48
12	-	170.35
13	5.74, d (2.9) 6.26, d (3.2)	122.69
14	1.91, s	25.39
15	1.77, s	24.61
1`	-	169.29
2`	2.14, s	21.27

#### Argyinolide S

Compound **1** was obtained as a white powder. The HRESIMS (m/z 322.1309 [M + Na]^+^, calculated for 322.14) and NMR data confirmed its molecular formula of C_17_H_22_O_6_, indicating 6 degrees of unsaturation. The ^1^H NMR spectrum (600 MHz, CDCl_3_) (Table 2) showed three methyl signals at *δ*_H_ 2.14 (s, 3 H), 1.91 (s, 3 H), and 1.77 (d, 3 H), as well as two terminal olefinic protons at *δ*_H_ 6.26 (d, *J* = 3.2 Hz, 1H) and 5.74 (d, *J* = 2.9 Hz, 1H). The ^13^C NMR spectrum (150 MHz, CDCl_3_) (Table 2) indicated 17 carbon signals, including three methyl carbons, two methylene carbons, five methine carbons, and five quaternary carbons. Within the quaternary carbons, there were two ester carbonyl carbons (*δ*_C_ 170.35, 169.29), two tetrasubstituted double bond carbons (*δ*_C_ 127.00, 133.86), two disubstituted double bond carbons (*δ*_C_ 136.48, 122.69), and one oxygen-bearing carbon (*δ*_C_ 80.22). The NMR data indicated that compound 1 belongs to the sesquiterpenoid class and has a planar structure similar to Argyinolide G [37], with the only difference being the attachment of Me-14 to C-3 (*δ*_C_ 80.22). In the HMBC spectrum, correlations between H-13 and C-7/C-11/C-12 supported the presence of a partial *α*,*β*-unsaturated *γ*-lactone moiety. The correlation between Me-15 and C-1/C-10 suggested a double bond between C-1 and C-10, while the correlation between Me-14 and C-3/C-4 indicated a hydrogen substituent on C-3. Other correlations, such as H-9 with C-10 and H-6 with C-1, provided additional information on molecular connectivity. COSY correlations from H-4/H-5/H-6/H-7/H-8/H-9 confirmed the structural elucidation (Fig. S51a). The relative configuration of compound **1** was inferred from its NOESY spectrum (Fig. S51b). Following the established rule that H-7 in natural guaiane-type sesquiterpenoids is always in the α-orientation [37], the signal relationship between H-4/H-6/H-8 suggested the same orientation for the hydroxyl group, indicating that H-4/H-6/H-8 adopt the *β*-orientation. The correlation between H-5 and H-7 indicated the same orientation for the *α*-orientation. The absolute configuration of compound **1** was determined by DP4 + analysis and further validated using ECD spectral fitting (Figs. S51c and d). Therefore, the structure of compound **1** was elucidated as (3*R*,4*S*,5*R*,6*R*,7*R*,8*S*)-8,9-dihydroxy-6,8-dimethyl-3-methylene-2-oxo-2,3,3a,4,5,7,8,9,9a,9b-decahydroazuleno[4,5-b]furan-4-yl acetate and named argyinolide S.

**Table 3:** ^1^H NMR (600 MHz) and ^13^C NMR (150 MHz) data (*δ*) for compound **2** in CD_3_OD (*δ* in ppm, *J* in hz)

NO	2	
	*δ*_H_ (*J* in Hz)	*δ*_C_
1	-	139.23
2	2.30, d(16.9) 2.80, d(12.6)	39.93
3	3.70, d(4.2)	80.01
4	-	83.95
5	2.80 d(12.6)	53.92
6	4.06, t(10.2)	80.87
7	3.28, m	53.83
8	4.86, m	72.04
9	2.24, dd(14.1, 2.8) 2.48, t(12.4)	42.58
10	-	126.29
11	-	138.95
12	-	171.48
13	5.70, d(2.9) 6.12, d(3.2)	121.43
14	1.76, s	23.78
15	1.54, s	23.36
1`	-	177.81
2`	2.63, m	35.34
3`	1.21, t(6.9)	19.45
4`	1.21, t (6.9)	18.89

#### Argyinolide T

Compound **2** was obtained as a white powder. Analysis by HRESIMS (m/z 351.1802 [M + H]^+^, calculated for 351.18) and NMR data revealed its molecular formula as C_19_H_26_O_6_ with a degree of unsaturation of 7. In the ^1^H NMR spectrum (600 MHz, CD_3_OD) (Table 3), four methyl proton signals were observed at *δ*_H_ 1.76 (s, 3H), *δ*_H_ 1.54 (s, 3H), and *δ*_H_ 1.21 (t, *J* = 6.9 Hz, 6H). Two olefinic methine proton signals were observed at *δ*_H_ 6.12 (d, *J* = 3.2 Hz, 1H) and *δ*_H_ 5.70 (d, *J* = 2.8 Hz, 1H). The tertiary carbon hydrogen signal connected to the hydroxyl group at C-3 exhibited a signal at *δ*_H_ 3.70 (d, *J* = 4.2 Hz, 1H), while the signals at *δ*_H_ 4.87 (m, 1H) and *δ*_H_ 4.06 (t, *J* = 10.2 Hz, 1H) indicated the presence of oxygen-bearing tertiary carbon protons. The ^13^C NMR spectrum (150 MHz, CD_3_OD) (Table 3) displayed a total of 19 carbon signals, including carbonyl signals from two ester groups (*δ*_C_ 177.81, 171.48), disubstituted olefinic carbons (*δ*_C_ 138.95, 121.43), and tetrasubstituted olefinic carbons (*δ*_C_ 139.23, 126.29). Tertiary carbon signals connected to the hydroxyl group were observed at *δ*_C_ 83.95 and *δ*_C_ 80.01, while oxygen-bearing tertiary carbon signals were observed at *δ*_C_ 80.87 and *δ*_C_ 72.04. A search of the NMR data revealed significant structural similarity between compound **2** and the known compound Argyinolide H [37], with the only difference being the absence of a methyl group at C-4’. Hence, compound **2** also belongs to the sesquiterpenoid class of labdane-type compounds. The COSY spectrum exhibited correlations between H-6/H-7/H-8/H-9 and H-2’/H_3_-3’/H_3_-4’. The HMBC spectrum displayed correlations between H-2’ (*δ*_H_ 2.63), H_3_-4’ (*δ*_H_ 1.21), and C-1’ (*δ*_C_ 177.81), among others (Fig. S52a). NOESY signals indicated that H-8 shares the same β-orientation as H-6, while H-3/H-5/H-7 share the same α-orientation (Fig. S52b). The absolute configuration was determined by DP4 + analysis and further confirmed through ECD spectral fitting (Figs. S5c and d). Compound **2** is a previously unreported novel compound named (3*S*,4*S*,5*S*,6*S*,7*R*,8*S*)-8,9-dihydroxy-6,9-dimethyl-3-methylene-2-oxo-2,3,3a,4,5,7,8,9,9a,9b-decahydroazuleno[4,5-b]furan-4-yl isobutyrate and named argyinolide T.

#### Identification of known compounds

Fifteen known compounds were identified as artemvulactone N (**3**) [20], moxartenolide (**4**) [38], Artemisiane E (**5**) [17], artemvulactone S (**6**) [20], 3*α*-chloro-4*β*,10*α*-dihydroxy-1*β*,2*β*-epoxy-5*α*,7*α*H-guai-11(13)-en-12,6*α*-olide (**7**) [39], 3*β*-chloro-4*α*,10*α*-dihydroxy-1*α*,2*α*-epoxy-5*α*,7*α*H-guaia-11(13)-en-12,6*α*-olide (**8**) [40], 11,13-dehydro-desacetylmatricarin (**9**) [41], 3-*β*-methoxy-iso-seco-tanapartholide (**10**) [42], (4S*,5S*)-dihydro-5-[(1R*,2S*)-2-hydroxy-2-methyl-5-oxo-3-cyclopenten-1-yl]-3-methylene-4-(3-oxobutyl)-2(3*H*)-furanone (**11**) [43], douglanin (**12**) [44], santamarine (**13**) [45], (3a*S*,6*E*,9*S*,10*E*,11a*R*)-9-(Acetyloxy)-3a,4,5,8,9,11a-hexahydro-6,10-dimethyl-3-methylenecyclodeca[*b*]furan-2(3*H*)-one (**14**) [46], Cyclodeca[b]furan-2(3 H)-one, 3a,4,5,8,9,11a-hexahydro-9-hydroxy-3,6,10-trimethyl-, (3*S*,3a*S*,6*E*,9*S*,10*E*,11a*S*)- (ACI) (**15**) [47], *rel*-(1*R*,5*S*,8*E*)-10,10-Dimethyl-2,6-bis(methylene)-8-cycloundecene-1,5-diol (**16**) [48], (+)-3-hydroxy-*β*-ionone (**17**) [49].

### Evaluation of anti-inflammatory activity in an LPS-induced BV-2 cell inflammation model

**Fig. 2:**
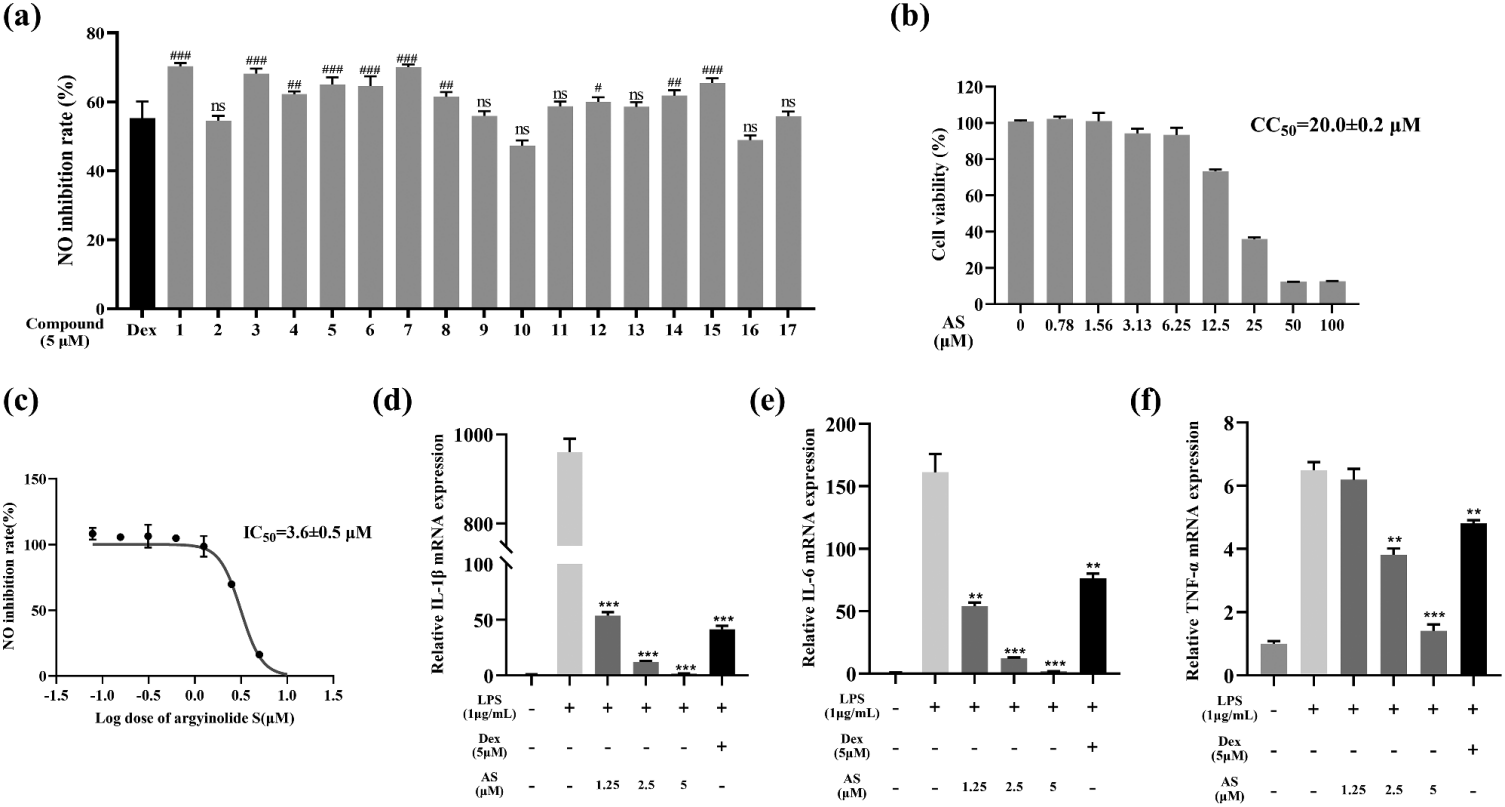
Results of the anti-inflammatory activity screening of the LPS-induced BV-2 microglial cell inflammation model. (**a**) Inhibition of NO by all the tested compounds at 5 µM. (**b**) Cytotoxicity of AS in BV-2 cells. (**c**) IC_50_ curve of AS mediated inhibition of NO production. (**d-f**) Inhibitory effect of AS on inflammatory factors at the mRNA level. (#p < 0.05, ##p < 0.01, ###p < 0.001, compared with the Dex group, *p < 0.05, **p < 0.01, ***p < 0.001, compared with the LPS group)

To assess the inhibitory potential of these compounds on nitric oxide production, a Griess assay was conducted at a concentration of 5 µM using an LPS-induced BV-2 cell inflammation model. The results demonstrated that six compounds (**1**, **3**, **5**, **6**, **7** and **17**), particularly compounds **1**, **3**, and **7**, exhibited notable advantages over the positive control drug dexamethasone in terms of nitric oxide inhibition at this concentration (Fig. 2a). In previous investigations, the anti-inflammatory activity of compounds 3 and 7 was partially elucidated [20, 50], whereas the structure and activity of AS were newly characterized, prompting further exploration of AS. Through CCK8 assay analysis, AS was found to exhibit a CC_50_ value of 20.0 ± 0.2 µM against BV-2 cells (Fig. 2b), with no significant cytotoxic effects observed at concentrations below 6.25 µM. Further investigation in the LPS-induced BV-2 cell inflammation model revealed, through the Griess assay, that AS exhibited an IC_50_ value of 3.6 ± 0.5 µM in inhibiting NO production (Fig. 2c). Additionally, qRT‒PCR analysis revealed significant downregulation of the mRNA levels of inflammatory cytokines, such as interleukin-1 beta (IL-1β), interleukin-6 (IL-6), and tumor necrosis factor-alpha (TNF-α), by AS (Fig. 2d‒f).

### Identifying the key targets of AS action on neuroinflammation

**Fig. 3:**
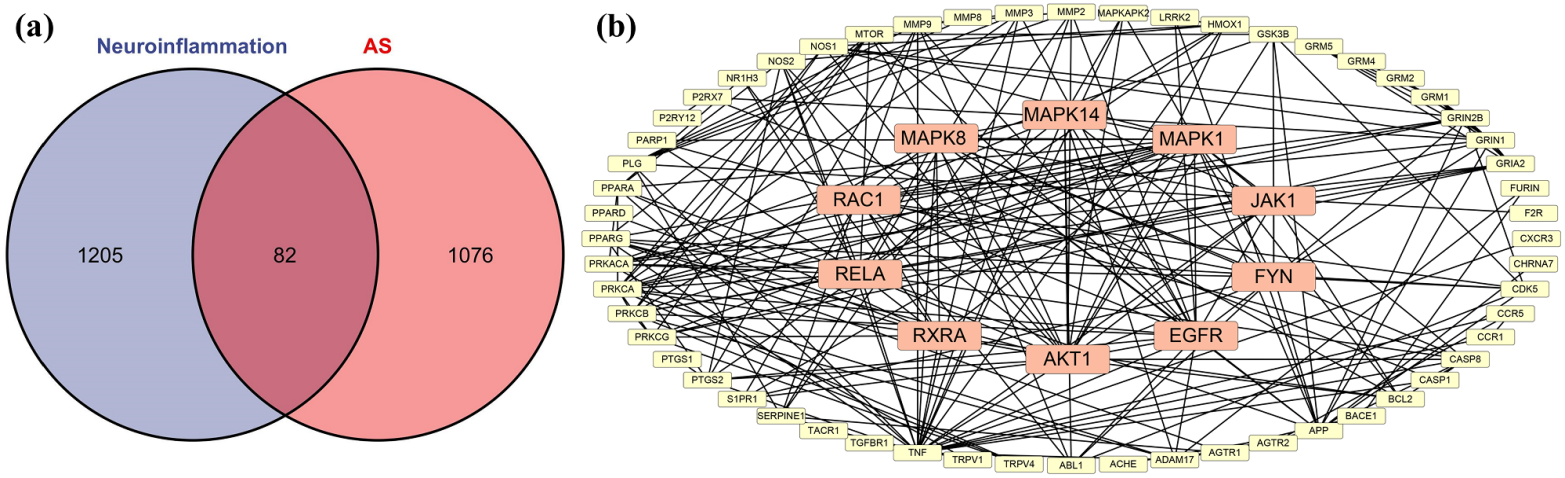
Intersection and key targets of neuroinflammation and AS. (**a**) Intersecting targets of AS and neuroinflammation. (**b**) Key targets of the intersection targets

After conducting a comprehensive analysis, a total of 1287 genes associated with neurological inflammation were identified. The target prediction analysis identified 1158 potential targets that AS could bind to. By comparing these two sets, a subset of 82 overlapping targets was identified (Fig. 3a). Subsequently, the STRING database was used to generate a protein-protein interaction (PPI) network. Core targets were determined based on multiple scoring methods including MCC, DMNC, MNC, and degree scores, using the cytoHubba plugin. Network reconstruction via Cytoscape revealed AKT1, EGFR, FYN, JAK1, MAPK1, MAPK14, MAPK8, MMP9, RAC1, RELA, and RXRA as key components of the network (Fig. 3b). These 11 targets may represent the core targets through which AS exerts its antineuroinflammatory effects.

### Molecular docking

**Fig. 4:**
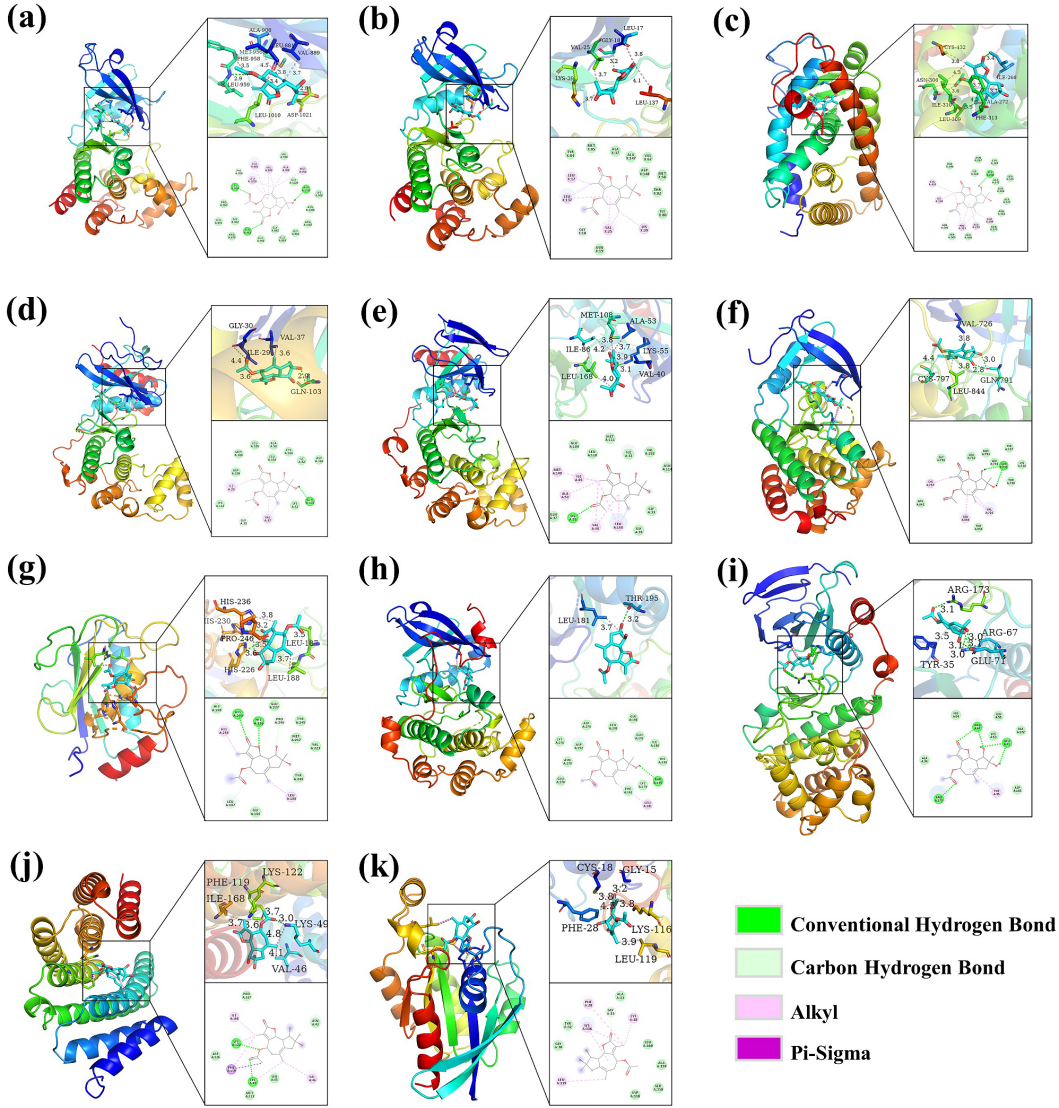
Molecular docking patterns of AS and key targets. (**a**) AS-JAK1. (**b**) AS-FYN. (**c**) AS-RXRA. (**d**) AS-MAPK1. (**e**) AS-MAPK8. (**f**) AS-EGFR. (**g**) AS-MMP9. (**h**) AS-AKT1. (**i**) AS-MAPK14. (**j**) AS-RELA. (**k**) AS-RAC1

The binding affinity of AS for 11 targets was found to be less than -6.0 kcal/mol, with the strongest binding observed for JAK1 at -10.1 kcal/mol, followed by FYN at -8.5 kcal/mol. Subsequent targets RXRA, MAPK1, and MAPK8 exhibited binding energies of -7.9 kcal/mol each, while binding energies with EGFR and MMP9 were -7.7 kcal/mol. The binding energy with AKT1 was determined to be -7.3 kcal/mol, whereas the binding energies with MAPK14, RELA, and RAC1 were all above -7.0 kcal/mol. Visual analysis revealed that AS primarily interacts via hydrogen bonding, carbon hydrogen bonding, and alkyl interactions with the target protein (Fig. 4).

### Molecular dynamics simulation and MM-PBSA

**Fig. 5:**
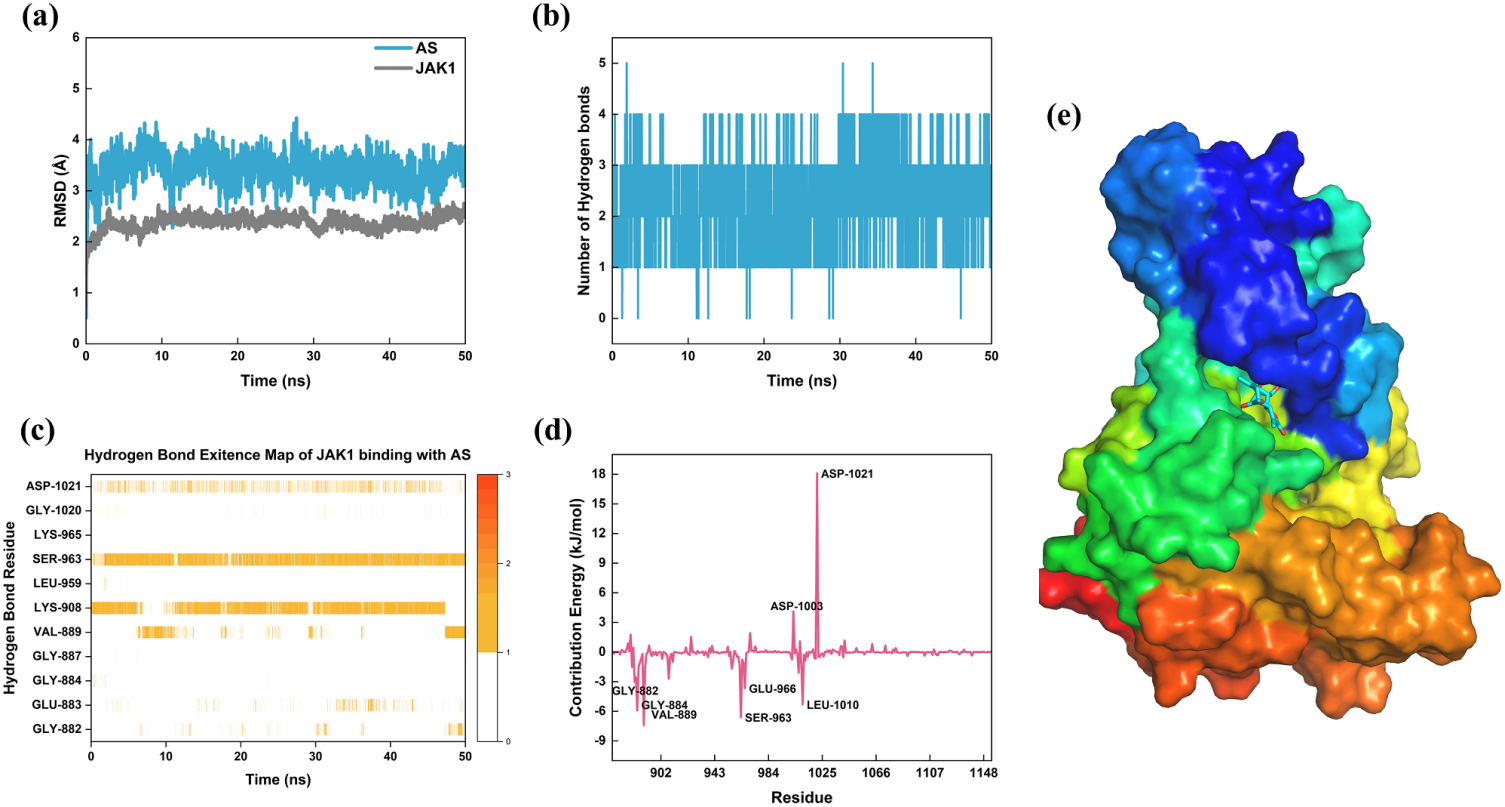
Analysis of molecular dynamics simulation results for AS binding with JAK1. (**a**) RMSD curves of AS and JAK1. (**b**) Number of hydrogen bonds in the simulation. (**c**) Hydrogen bond connections in the whole simulation process. (**d**) Results of MM-PBSA free energy decomposition into residues. (**e**) Binding mode of AS to JAK1

Based on the molecular docking results, a significant binding affinity between AS and JAK1 was discovered. Therefore, we conducted molecular dynamics simulations and MM-PBSA calculations to analyze the stability of the AS-JAK1 complex. During the 50 ns molecular dynamics simulation, the AS-JAK1 complex consistently maintained stable binding, with the root-mean-square deviation (RMSD) fluctuating within 1 Å (Fig. 5a). The hydrogen bond analysis revealed that throughout the simulation, stable hydrogen bonds formed between AS and JAK1’s LYS908 and SER963, while the hydrogen bonds with ASP1021 continually broke and reformed (Fig. 5b and c). Using MM-PBSA, the binding free energy between AS and JAK1 was determined to be -84.477 kJ/mol, indicating a fairly strong binding affinity. Upon decomposing the energy contribution by residues, six amino acid residues (GLY882, GLY884, VAL889, SER963, GLU966, and LEU1010) favor the stability of the complex. Conversely, the presence of two ASP residues, 1003 and 1021, adversely affects the stability of the complex, particularly for ASP1021 (Fig. 5d).

### AS suppresses BV-2 cell inflammatory response via the JAK1/STAT3 signaling pathway

**Fig. 6:**
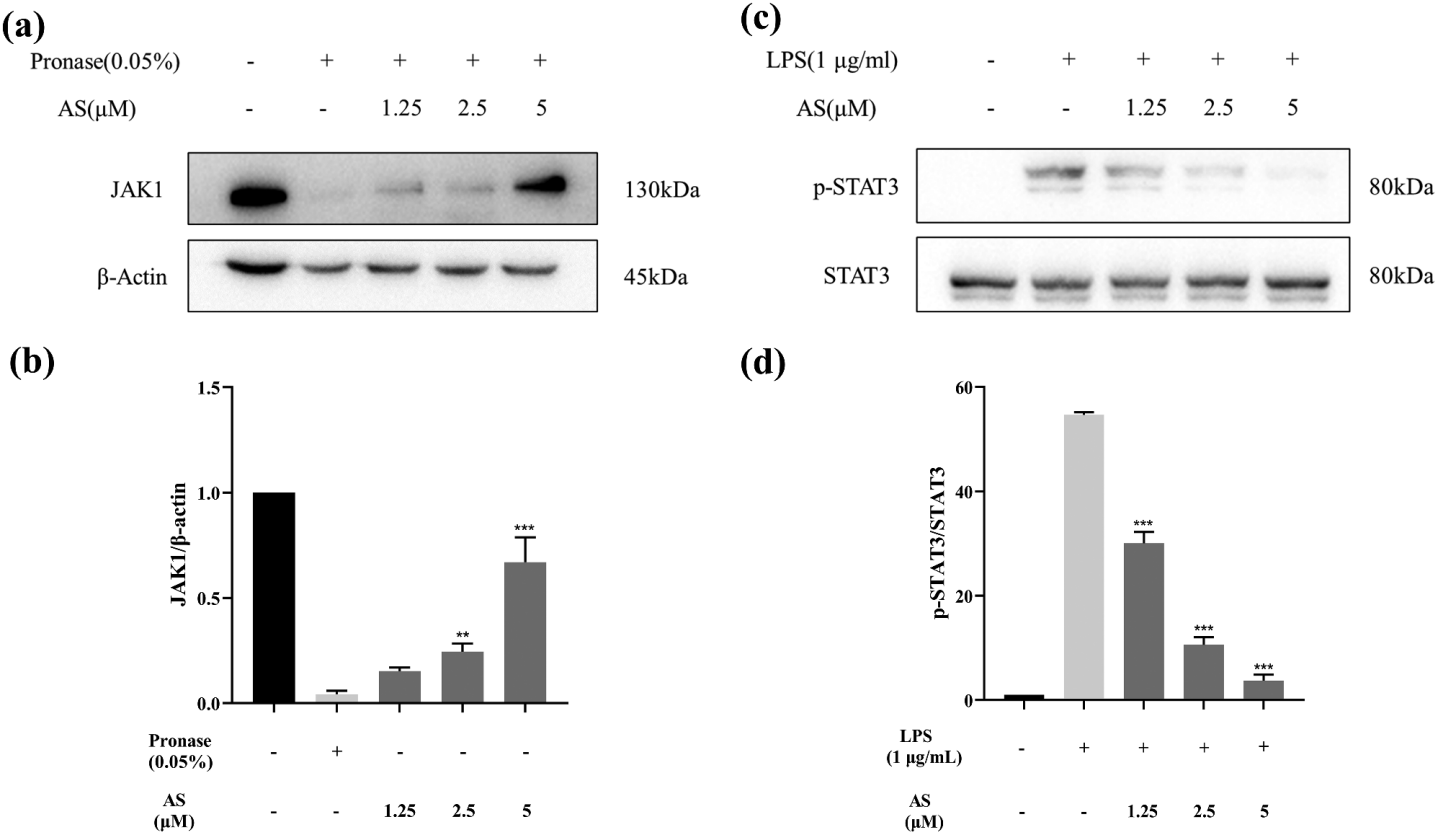
AS regulates the JAK1/STAT3 signaling pathway. (**a-b**) AS directly binds to JAK1. (**c-d**) AS inhibited the phosphorylation of STAT3 (*p < 0.05, **p < 0.01, ***p < 0.001, compared with the pronase or LPS model group)

The computational simulation results suggested that AS may exert its antineuroinflammatory effects through binding to JAK1. This hypothesis was substantiated by the application of DARTS and Western blot assays. The findings demonstrated that AS preserves the integrity of JAK1 against pronase digestion, indicative of a direct interaction between AS and JAK1 (Fig. 6a and b). Additionally, AS significantly inhibits the phosphorylation of the downstream protein, STAT3, mediated by JAK1(Fig. 6c and d).

## Discussion

Pathogen- or tissue-induced immunogenic stimulation triggers inflammation, resulting in various cascading reactions that maintain homeostasis within the body. In the brain, neuroinflammation is typically manifests as by elevated levels of proinflammatory cytokines, activation of microglia, peripheral nerve damage, and leukocyte infiltration [51]. Microglia serve as resident immune cells of the central nervous system. They play roles in detecting environmental changes, synaptic remodeling, maintaining myelin homeostasis, and recognizing pathogen- and damage-associated molecular patterns (PAMPs and DAMPs), thereby participating in the maintenance of homeostasis and defense mechanisms [52–54]. Typically, microglia in a stable brain exhibit a highly branched morphology. However, upon activation by external stimuli, these cells rapidly transform into an amoeboid shape and produce large quantities of TNF-α, IL-1β, interleukin-16 (IL-16), and chemotactic factors, including C-C motif chemokine ligand 2 (CCL2) and interleukin-18 (IL-18). Simultaneously, they recruit more cells to the damaged site for pathogen clearance [5]. Although neuroinflammation may serve as a protective mechanism, sustained neuroinflammation can induce neurotoxicity and trigger neurodegenerative diseases. Previous studies have indicated that excessive activation of microglia can lead to neuronal loss and damage. Therefore, the inhibition of inflammation mediated by microglia is a promising novel therapeutic strategy [55].

Herbal medicines derived from natural sources serve as the foundation of traditional Asian medicine, gradually gaining worldwide acceptance for their remarkable therapeutic effects and lower incidence of adverse reactions [56]. An emerging field of research focuses on the discovery of neuroprotective plant compounds as novel candidates for anti-neuroinflammatory therapy [57]. *Artemisia argyi*, a perennial herb of the Asteraceae family, possesses a long history of medicinal use and is commonly employed in the clinical treatment of asthma, dysmenorrhea, malaria, and influenza [13, 58]. Modern pharmacological studies have demonstrated that dried leaves of *Artemisia argyi*, contain a variety of chemical constituents, showcasing diverse pharmacological activities such as anti-inflammatory, antioxidant, and anti-tumor properties [13, 58–60]. In this study, we isolated and purified 17 structurally distinct sesquiterpenoid compounds from *Artemisia argyi* leaves and evaluated their effects on the inflammatory response of microglia stimulated with LPS. NO serves as a crucial mediator in the inflammatory response, generated by the activation of inducible nitric oxide synthase (iNOS), and its release is often used as an indicator of the degree of inflammation [61]. Under fixed concentrations, 10 compounds exhibited significantly greater inhibition of NO production compared to dexamethasone, with the novel compound AS demonstrating the most potent inhibition of NO. Among the 17 sesquiterpenes examined in this study, tricyclic and germacrane-type sesquiterpenes demonstrated superior anti-inflammatory activity, with their appended side chains and configurations significantly influencing their efficacy. Compounds 1 and 2 share a similar core structure, yet exhibit substantial differences in activity, underscoring the critical role of methyl attachment positions, hydroxyl configurations, and functional groups linked at the C-8 position in their anti-inflammatory potency. This phenomenon is also evident in compounds 7 and 8, where distinct configurations at carbons 2, 3, and 4 contribute to notable variations in activity. Further investigations confirmed that AS markedly suppresses the production of inflammatory factors IL-1β, IL-6, and TNF-α.

Evidence derived from computational analysis suggests that JAK1 may serve as a putative target for AS, undergoes processes of dimerization, phosphorylation, and activation upon the binding of cytokines to their corresponding receptors, subsequently leading to the induction of phosphorylation in members of the STAT family. The JAK-STAT signaling pathway represents a highly significant cellular communication hub that plays a critical role in regulating various subsequent processes, including immune adaptation, tissue repair, and inflammatory responses. Perturbations in the JAK/STAT signaling pathway bear pathological relevance particularly in the context of neuroinflammatory diseases. Prior studies have demonstrated the inhibitory impacts of JAK1 inhibitors on microglial cell inflammatory responses, concomitantly facilitating neural function restoration and ameliorating related symptoms in animal models of neurodegenerative disorders [62–65]. DARTS experimentation, as an important approach for exploring small molecule targets, enables the revelation of drug-target interactions within cells or tissues by monitoring variations in the stability of proteins serving as receptors for biologically active small molecules, all without necessitating additional modifications of the small molecules themselves [66]. This methodology assumes critical significance in elucidating the mechanisms of action of bioactive natural compounds with unknown modes of action. Through the integration of this method with computer-assisted target exploration, the direct targeting of JAK1 by AS has been substantiated. Subsequent Western blot experimentation has uncovered the suppressive impact of AS on the phosphorylation of downstream STAT3 protein, underscoring the critical involvement of the JAK1/STAT3 signaling pathway in mediating AS’s inhibitory effects on BV-2 cell inflammatory responses. Although our research offers significant findings regarding the anti-inflammatory characteristics of AS in BV-2 cells, it is essential to recognize the constraints of the in vitro model and underscore the necessity for additional confirmation in more intricate neuronal environments.

## Conclusion

Natural products serve as important sources for drug development. In this study, we isolated and purified 17 sesquiterpenoids from *Artemisia argyi*. The anti-inflammatory activity of these compounds was examined, and it was observed that six of them exhibited superior performance compared to dexamethasone. Particularly noteworthy, our investigation led to the discovery of a novel sesquiterpene lactone named AS. AS demonstrated the most potent antineuroinflammatory effect among all the compounds tested. Moreover, advanced bioinformatics techniques were employed to elucidate the main molecular target of AS as JAK1. Importantly, the computational simulation results were validated by experimental data. DARTS and Western blot experiments demonstrated that AS exerted anti-inflammatory effects through the inhibition of the JAK1/STAT3 pathway.

In summary, our study underscores the inhibitory effect of sesquiterpenoids from *Artemisia argyi* on the inflammatory response of BV-2 cells, laying a theoretical foundation for further investigation into the antineuroinflammatory properties of sesquiterpenoids from *Artemisia argyi*.

### Electronic supplementary material

Below is the link to the electronic supplementary material.

Supplementary Material 1

Supplementary Material 2

## Data Availability

The datasets used and/or analysed during the current study are available from the corresponding author on reasonable request.

## Abbreviations

AS: Argyinolide S
DARTS: Drug affinity responsive target stability
AKT1: Protein kinase B 1
EGFR: Epidermal growth factor receptor
FYN: Proto-oncogene tyrosine-protein kinase
JAK1: Janus kinase 1
MAPK: Mitogen-activated protein kinase
MMP9: Matrix metalloproteinase 9
RAC1: Ras-related C3 botulinum toxin substrate 1
RELA: Nuclear factor kappa-B p65
RXRA: Retinoid X receptor alpha
PPI: Protein-protein interactions
AS: Argyinolide S
CNS: Central nervous system
LPS: Lipopolysaccharide
STAT3: Signal transducer and activator of transcription 3
NF-κB: Nuclear factor kappa-B
JNK: c-JunN-terminal Kinase/p38
NLRP3: NOD-like receptor thermal protein domain associated protein 3
RAW264.7: Mouse RAW264.7 Macrophages
BV-2: C57BL/6 murine microglia
PE: Petroleum ether
EA: Ethyl acetate
NMR: Nuclear magnetic resonance
CD: Circular dichroism
HRESIMS: High-resolution electrospray ionization mass spectrometry
HPLC: High-performance liquid chromatography
ECD: Electronic circular dichroism
NO: Nitric oxide
MM-PBSA: Molecular mechanic/Poisson-Boltzmann surface area
CCK8: Cell Counting Kit-8
qRT-PCR: Quantitative Real-Time PCR
cDNA: Complementary DNA
BCA: Bicinchoninic acid
SDS-PAGE: Sodium dodecyl sulfate -polyacrylamide gel electrophoresis
IL-1β: Interleukin-1 beta
IL-6: Interleukin-6
TNF-α: Tumor necrosis factor-alpha
PAMPs and DAMPs: Pathogen- and damage-associated molecular patterns
IL-16: Interleukin-16
CCL2: C-C motif chemokine ligand 2
DEPT: Distortionless Enhancement by Polarization Transfer
IL-18: Interleukin-18
iNOS: Inducible nitric oxide synthase
