# Supplementary material for: Isolation and anti-neuroinflammation activity of sesquiterpenoids from Artemisia argyi: computational simulation and experimental verification
Source: BMC Complement Med Ther. 2024 Jul 11;24:264. doi: 10.1186/s12906-024-04578-z (PMC11238432; doi:10.1186/s12906-024-04578-z)
Supplement: Supplementary file 1 — Supplementary Material 1 [file 12906_2024_4578_MOESM1_ESM.docx]

**Isolation and Anti-Neuroinflammation Activity of Sesquiterpenoids from *Artemisia argyi*: Computational Simulation and Experimental Verification**

**Caiwenjie La ^a,b,1^, Menghe Li ^a,b^****^,1^, Zexu Wang ^a,b^, Tao Liu ^a,b^, Qiongzhen Zeng^a,c^, Pinghua Sun^d^, Zhe Ren ^a,b^, Cuifang Ye ^a,b^, Qiuying Liu, ^a,b,*^, Yifei Wang ^a,b*^**

^a^ Department of Cell Biology, College of Life Science and Technology, Jinan University, Guangzhou, China.

^b^ Guangdong Provincial biotechnology drug & Engineering Technology Research Center, Guangzhou, China.

^c^ Shenzhen People's Hospital (The Second Clinical Medical College, Jinan University; The First Affiliated Hospital, Southern University of Science and Technology), Shenzhen518020, China.

^d^ College of Pharmacy, Jinan University, Guangzhou, 510632, China

^1^ These authors have contributed equally to this work and share the first authorship

^*^ Corresponding author: Qiuying Liu, Yifei Wang.

Address: Institute of Biomedicine, College of Life Science and Technology, Jinan University, No. 601, Whampoa Road West, Guangzhou, 510632, PR China.

Tel/fax: +86 20 85227715 (Qiuying Liu), +86 20 85223426 (Yifei Wang).

E-mail address: qiuying_liu@126.com (Qiuying Liu), twang-yf@163.com (Yifei Wang).

[Supplementary Extraction and isolation 5](#_Toc169265644)

[Supplementary NMR data 8](#_Toc169265645)

[Figure S1. ^1^H-NMR spectrum of compound 1（600 MHz, CDCl_3_） 8](#_Toc169265646)

[Figure S2. ^13^C-NMR spectrum of compound 1（150 MHz, CDCl_3_） 8](#_Toc169265647)

[Figure S3. HSQC spectrum of compound 1 (600 MHz, CDCl_3_) 9](#_Toc169265648)

[Figure S4. ^1^H-^1^H COSY spectrum of compound 1 (600 MHz, CDCl_3_) 9](#_Toc169265649)

[Figure S5. HMBC spectrum of compound 1 (600 MHz, CDCl_3_) 10](#_Toc169265650)

[Figure S6. NOESY spectrum of compound 1 (600 MHz, CDCl_3_) 10](#_Toc169265651)

[Figure S7. DEPT135 spectrum of compound 1（150 MHz, CDCl_3_） 11](#_Toc169265652)

[Figure S8. HRESIMS spectrum of compound 1 11](#_Toc169265653)

[Figure S9. DP4+ result of compound 1 12](#_Toc169265654)

[Figure S10. NMR fitted curves of compound 1 13](#_Toc169265655)

[Figure S11. ^1^H-NMR spectrum of compound 2（600 MHz, CD_3_OD） 13](#_Toc169265656)

[Figure S12. ^13^C-NMR spectrum of compound 2（150 MHz, CD_3_OD） 14](#_Toc169265657)

[Figure S13. HSQC spectrum of compound 2（600 MHz, CD_3_OD） 15](#_Toc169265658)

[Figure S14. ^1^H-^1^H COSY spectrum of compound 2（600 MHz, CD_3_OD） 15](#_Toc169265659)

[Figure S15. HMBC spectrum of compound 2（600 MHz, CD_3_OD） 15](#_Toc169265660)

[Figure S16. NOESY spectrum of compound 2（600 MHz, CD_3_OD） 16](#_Toc169265661)

[Figure S17. DEPT135 spectrum of compound 2（150 MHz, CD_3_OD） 16](#_Toc169265662)

[Figure S18. HRESIMS spectrum of compound 2 17](#_Toc169265663)

[Figure S19. DP4+ result of compound 2 18](#_Toc169265664)

[Figure S20. NMR fitted curves of compound 2 19](#_Toc169265665)

[Figure S21. ^1^H-NMR spectrum of compound 3（600 MHz, CDCl_3_） 19](#_Toc169265666)

[Figure S22. ^13^C-NMR spectrum of compound 3（150 MHz, CDCl_3_） 20](#_Toc169265667)

[Figure S23. ^1^H-NMR spectrum of compound 4（600 MHz, CDCl_3_） 20](#_Toc169265668)

[Figure S24. ^13^C-NMR spectrum of compound 4（150 MHz, CDCl_3_） 21](#_Toc169265669)

[Figure S25. ^1^H-NMR spectrum of compound 5（600 MHz, CDCl_3_） 21](#_Toc169265670)

[Figure S26. ^13^C-NMR spectrum of compound 5（150 MHz, CDCl_3_） 22](#_Toc169265671)

[Figure S27. ^1^H-NMR spectrum of compound 6（600 MHz, CDCl_3_） 22](#_Toc169265672)

[Figure S28. ^13^C-NMR spectrum of compound 6（150 MHz, CDCl_3_） 23](#_Toc169265673)

[Figure S29. ^1^H-NMR spectrum of compound 7（600 MHz, CDCl_3_） 23](#_Toc169265674)

[Figure S30. ^13^C-NMR spectrum of compound 7（150 MHz, CDCl_3_） 24](#_Toc169265675)

[Figure S31. ^1^H-NMR spectrum of compound 8（600 MHz, CDCl_3_） 24](#_Toc169265676)

[Figure S32. ^13^C-NMR spectrum of compound 8（150 MHz, CDCl_3_） 25](#_Toc169265677)

[Figure S33. ^1^H-NMR spectrum of compound 9（600 MHz, CD_3_OD） 25](#_Toc169265678)

[Figure S34. ^13^C-NMR spectrum of compound 9（150 MHz, CD_3_OD） 26](#_Toc169265679)

[Figure S35. ^1^H-NMR spectrum of compound 10（600 MHz, CDCl_3_） 26](#_Toc169265680)

[Figure S36. ^13^C-NMR spectrum of compound 10（150 MHz, CDCl_3_） 27](#_Toc169265681)

[Figure S37. ^1^H-NMR spectrum of compound 11（600 MHz, CDCl_3_） 27](#_Toc169265682)

[Figure S38. ^13^C-NMR spectrum of compound 11（150 MHz, CDCl_3_） 28](#_Toc169265683)

[Figure S39. ^1^H-NMR spectrum of compound 12（600 MHz, CDCl_3_） 28](#_Toc169265684)

[Figure S40. ^13^C-NMR spectrum of compound 12（150 MHz, CDCl_3_） 29](#_Toc169265685)

[Figure S41. ^1^H-NMR spectrum of compound 13（600 MHz, CDCl_3_） 29](#_Toc169265686)

[Figure S42. ^13^C-NMR spectrum of compound 13（150 MHz, CDCl_3_） 30](#_Toc169265687)

[Figure S43. ^1^H-NMR spectrum of compound 14（600 MHz, CDCl_3_） 30](#_Toc169265688)

[Figure S44. ^13^C-NMR spectrum of compound 14（150 MHz, CDCl_3_） 31](#_Toc169265689)

[Figure S45. ^1^H-NMR spectrum of compound 15（600 MHz, CDCl_3_） 31](#_Toc169265690)

[Figure S46. ^13^C-NMR spectrum of compound 15（150 MHz, CDCl_3_） 32](#_Toc169265691)

[Figure S47. ^1^H-NMR spectrum of compound 16（600 MHz, CDCl_3_） 32](#_Toc169265692)

[Figure S48. ^13^C-NMR spectrum of compound 16（150 MHz, CDCl_3_） 33](#_Toc169265693)

[Figure S49. ^1^H-NMR spectrum of compound 17（600 MHz, CDCl_3_） 33](#_Toc169265694)

[Figure S50. ^13^C-NMR spectrum of compound 17（150 MHz, CDCl_3_） 34](#_Toc169265695)

[Figure S51. The elucidation process of the structure of Compound 1 (argyinolide S). 34](#_Toc169265696)

[Figure S52. The elucidation process of the structure of Compound 2 (argyinolide T). 35](#_Toc169265697)

[Table S1 The RT-qPCR sequences used in the study 35](#_Toc169265698)

[Table S2 The antibodies used in the study 35](#_Toc169265699)

# Supplementary Extraction and isolation

The EA layer Fr.2 was subjected to MCI column (CH_3_OH-H_2_O, 50%-100%) to give Fr.2.1-2.8. Fr.2.6 (10.00 g) was further separated by ODS column chromatography with the mobile phase of MeOH-H_2_O (50%-100%) to give seven fractions Fr.2.6.1-2.6.7. Fr.2.6.4 (5.20 g) was separated on a silica gel column containing PE-EA (20:1-0:1) to give six fractions Fr.2.6.4.1-2.6.4.6. Fr.2.6.4.4 (1.852 g) was again separated on a silica gel column containing PE-EA to give 3 components Fr.2.6.4.4.1-Fr.2.6.4.4.3. Of these, Fr.2.6.4.4.2 (1.075 g) was purified by Sephadex LH-20 column chromatography (MeOH) to give 1.019 g, which was separated using HPLC (CH_3_CN-H_2_O, 55:45) to compound **3** (1.7 mg, t=22.93 min), compound **14** (2.6 mg, t=24.61 min). Fr.2.7 was subjected to ODS column (MeOH-H_2_O, 40%-100%) to obtain Fr.2.7.1-Fr.2.7.9, and Fr.2.7.6 (818 mg) was divided into 6 segments by mobile phase PE-acetone (20:1-0:1), and Fr.2.7.6.2 (514 mg) was divided into Fr.2.7.6.2.1 (60 mg) and Fr.2.7.6.2.2 (80 mg) by PTLC, Fr.2.7.6.2.2 was subjected to HPLC (CH_3_CN-H_2_O-58:42) to obtain compound **5** (2.6 mg, t=14.43 min), compound **4** (10.1 mg, t=15.74 min).

Fr.4 (108.00 g) was divided into Fr.4.1-Fr.4.6 by MCI column (MeOH-H_2_O, 50%-100%), and Fr.4.2 was divided into Fr.4.2.1-Fr.4.2.9 by passing through a silica gel column containing PE-EA (25:1-0:1). Fr.4.2.8 (7.732 g) was further divided into 6 segments by ODS column (MeOH-H_2_O,45%-100%), and Fr.4.2.8.1 and Fr.4.2.8.2 totaling 6.154g were combined and again divided into Fr.4.2.8.1.1-Fr.4.2.8.1.6 by ODS column (MeOH-H_2_O,45%-100%). Fr.4.2.8.1.2 (1.429 g) was separated by silica gel column chromatography with the eluent of PE- acetone (18:1-0:1) into Fr.4.2.8.1.2.1-Fr.4.2.8.1.2.5. Fr.4.2.8.1.2.5 (264 mg) was partitioned by Sephadex LH-20 column (MeOH) into Fr.4.2.8.1.2.5.1-Fr.4.2.8.1.2.5.3, where Fr.4.2.8.1.2.5.2 (160 mg) was purified by HPLC (CH_3_CN-H_2_O, 40:60) to give compound **7** (112.6 mg, t=23.83 min). Fr.4.2.8.1.4 (1.271 g) was subjected to silica gel column chromatography (PE-EA, 20:1-0:1). Divided into 5 segments, of which Fr.4.2.8.1.4.2 (49 mg) was purified by HPLC (CH_3_CN-H_2_O, 29:71) to give compound **17** (3.3 mg, t=24.94 min).

Fr.4.2.8.1.6 (175 mg) was separated by silica gel column (PE-EA, 15:1-0:1) to give Fr.4.2.8.1.6.1-Fr.4.2.8.1.6.4. Fr.4.2.8.1.6.3 was divided into 2 segments by gel column (MeOH) and Fr.4.2.8.1.6.3.1 ( 136 mg) was purified by HPLC (CH_3_CN-H_2_O, 30:70) to give compound **15** (3.1 mg, t=36.13 min). Fr.4.2.8.3 (883 mg) was separated by silica gel column (PE-acetone, 20:1-0:1) into 6 segments, Fr.4.2.8.3.5 (530 mg) was separated by Sephadex LH-20 column (MeOH) and divided into 2 segments, Fr.4.2.8.3.5.2 (490 mg) was purified by HPLC (CH_3_CN-H_2_O, 27:73) to give compound **16** (2.4 mg, t=56.39 min). Fr.4.3 (14.00 g) was subjected to ODS column (MeOH:H_2_O, 50%-100%,) Fr.4.3.1-Fr.4.3.5, Fr.4.3.2 (8.00 g) was divided into 5 segments by silica gel column (PE-EA, 20:1-0:1), and Fr.4.3.2.4 (1.002 g) was again divided into Fr.4.3.2.4.1 and Fr.4.3.2.4.2 by silica gel column chromatography (PE-acetone, 20:1-0:1). Fr.4.3.2.4.2 (661 mg) was again subjected to silica gel column (PE-acetone, 10:1-0:1) and then Sephadex LH-20 column (MeOH) to obtain Fr.4.3.2.4.2.2 (397 mg), which was purified by HPLC (MeOH-H_2_O, 53:47) to give compound **12** ( 2.4 mg, t=16.18 min), compound **13** (14.6 mg, t=29.89 min).Fr.4.3.3 (3.773 g) was divided into 6 segments by silica gel column chromatography (PE-EA, 20:1-0:1), Fr.4.3.3.4 (159 mg) was divided into Fr.4.3.3.4.1 and Fr.4.3.3.4.2 (87 mg) by gel column chromatography (MeOH). Fr.4.3.3.4.2 was purified by HPLC (CH_3_CN-H_2_O, 39:61) to give compound **1** (2.2 mg, t=31.32 min).

Fr.5 (350 g) was subjected to MCI column chromatography (MeOH-H_2_O, 50%-100%) divided into 7 segments Fr.5.1-5.7. Fr.5.2 (32.9 g) was subjected to ODS column chromatography (MeOH-H_2_O, 37%-46%) divided into Fr.5.2.1-5.2.8. Fr.5.2.2 (5.6 g) was subjected to silica gel column chromatography (PE-EA, 30:1-1:5) to give 9 segments (Fr.5.2.2.1-5.2.2.9). Fr.5.2.2.9 (1.786 g) was subjected to ODS column chromatography (MeOH-H_2_O, 27%-45%) to give 2 segments (Fr.5.2.2.9.1-5.2.2.9.2). Fr.5.2.2. 9.1 (934 mg) was chromatographed by Sephadex LH-20 column chromatography (MeOH) to give 3 segments (Fr.5.2.2.9.1.1-5.2.2.2.9.1.3), and Fr.5.2.2.9.1.1 (206 mg) was purified by HPLC (CH_3_CN-H_2_O, 45:55) to give compound **10** (4 mg, t=9.85 min). Fr.5.2.4 (4.069 g) was divided into 7 segments Fr.5.2.4.1-5.2.4.7 by silica gel column chromatography (PE-EA, 15:1-3:1), and Fr.5.2.4.3 (1.048 g) was subjected to ODS column chromatography (MeOH-H_2_O, 23%-48%) to give 3 segments (Fr.5.2.4.3.1-5.2.4.3.3), Fr.5.2.4.3.2 (389 mg) was purified by Sephadex LH-20 column chromatography (MeOH) to give 3 segments (Fr.5.2.4.3.2.1-5.2.4.3.2.3), Fr.5.2.4.3.2.2 (81 mg) was purified by HPLC (CH_3_CN-H_2_O, 20:80) to give compound **8** (7 mg, t=75.44 min). Fr.5.2.4.4 (1.153 g) was separated by ODS column chromatography (MeOH-H_2_O, 23%-47%) into 4 segments Fr.5.2.4.4.1-5.2.4.4.4. Fr.5.2.4.4.2 (361 mg) was separated by Sephadex LH-20 column chromatography (MeOH) to give 4 segments ( Fr.5.2.4.4.2.1- Fr.5.3.4.4.2.4), Fr.5.2.4.4.2.3 (227 mg) was purified by HPLC (CH_3_CN-H_2_O, 40:60) to give compound **11** (4 mg, t=26.91 min). Fr.5.3 (31.9 g) was separated by silica gel column chromatography (DCM: MeOH, 100:1-10:1) into 9 segments Fr.5.3.1-5.3.9. Fr.5.3.1 (5.319g) was divided into 8 segments by ODS column chromatography (MeOH-H_2_O, 40%-65%). Fr.5.3.1.4 (703mg) was divided into 8 segments by silica gel column chromatography (PE -EA, 10:1-1:4) to give 4 segments (Fr.5.3.1.4.1-5.3.1.4.4), and the third segment Fr.5.3.1.4.3 (299 mg) was separated by HPLC (CH_3_CN-H_2_O, 30:70) to give compound **9** (2 mg, t=34.36 min). Fr.5.3.2 (3.618 g) was subjected to ODS column chromatography (MeOH-H_2_O, 40%-70%) to obtain 5 segments (Fr.5.3.2.1-5.3.2.5). Fr.5.3.2.2 (1.896 g) was subjected to silica gel column chromatography (PE-EA, 8:1-1:3) to obtain 5 segments (Fr.5.3.2.2.1- 5.3.2.2.5), Fr.5.3.2.2.3 (509 mg) was subjected to Sephadex LH-20 column chromatography (MeOH) to obtain 2 segments (Fr.5.3.2.2.3.1- 5.3.2.2.3.2), and Fr.5.3.2.2.3.2 (402 mg) was subjected to HPLC purification (CH_3_CN-H_2_O, 40:60) to obtain compound **2** (6 mg, t=23.00 min).

The same previous isolation method was used for PE extracts. Fr.D.3 (16.8 g) was divided into Fr.D.3.1-Fr.D.3.7 by ODS column chromatography (MeOH-H_2_O, 60%-100%), Fr.D.3.3 (4.30 g) was then divided into 3 segments by silica gel column chromatography (PE-acetone, 100:1-0:1), and was again separated into Fr.D.3.3.2.3 (433 g) by silica gel column (PE-EA, 60:1- 0:1) for Fr.D.3.3.2 (2.1 g) to obtain Fr.D.3.3.2.3 (433 mg) was continued to be separated by silica gel column (PE-acetone, 30:1-0:1), and Fr.D.3.3.2.3.2 (140 mg) was purified by HPLC (MeOH-H_2_O, 59:41) to obtain compound **6** (2.0 mg, t=24.07 min).

# Supplementary NMR data


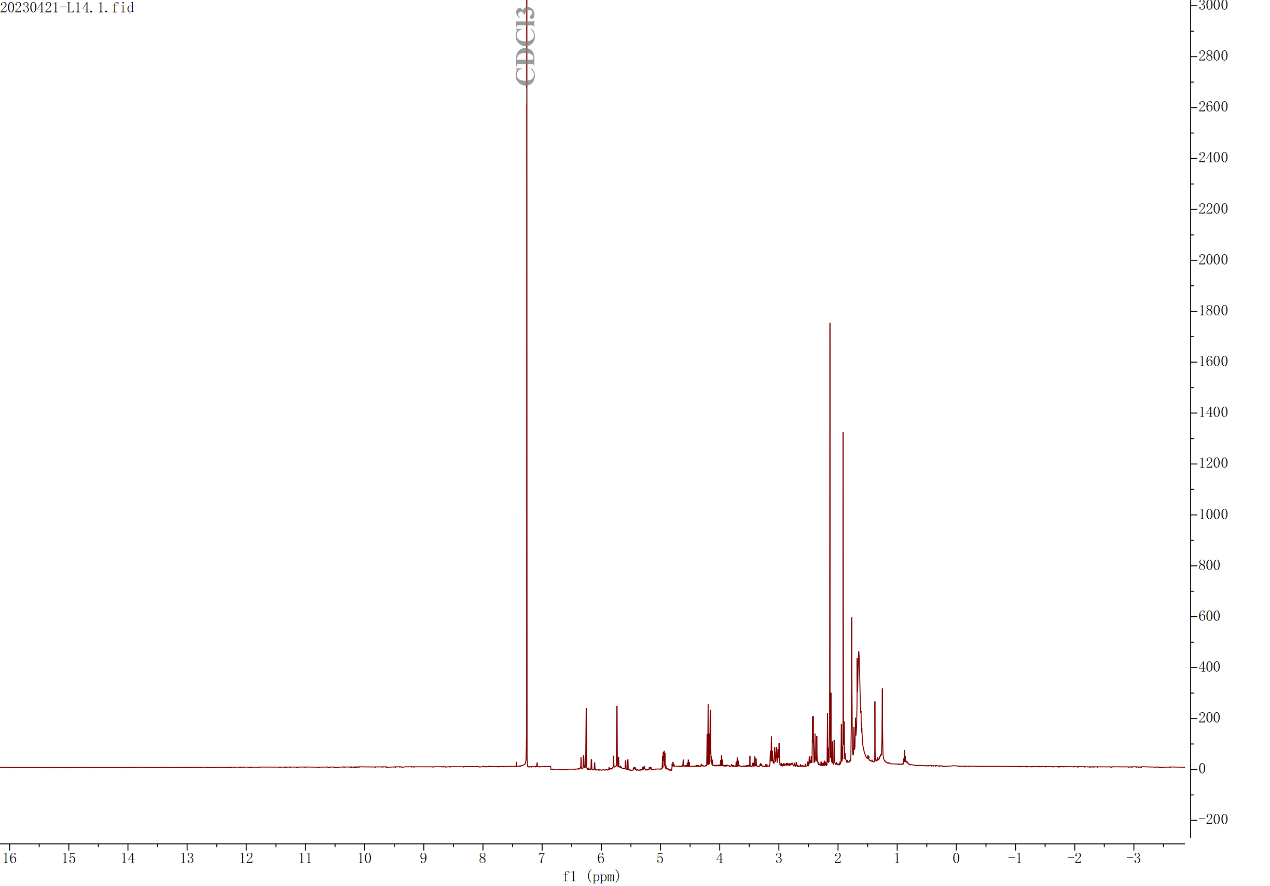


Figure S1. ^1^H-NMR spectrum of compound 1（600 MHz, CDCl_3_）


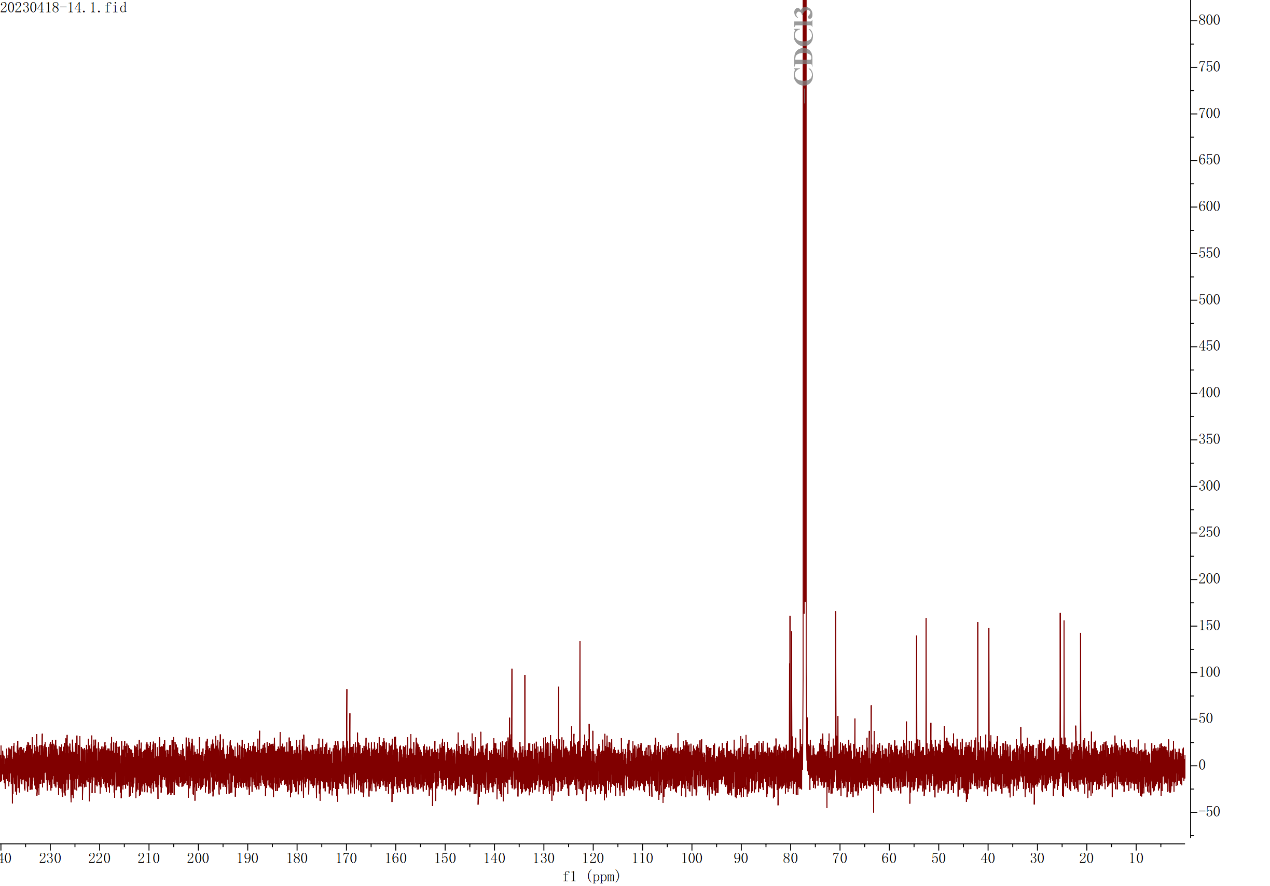


Figure S2. ^13^C-NMR spectrum of compound 1（150 MHz, CDCl_3_）


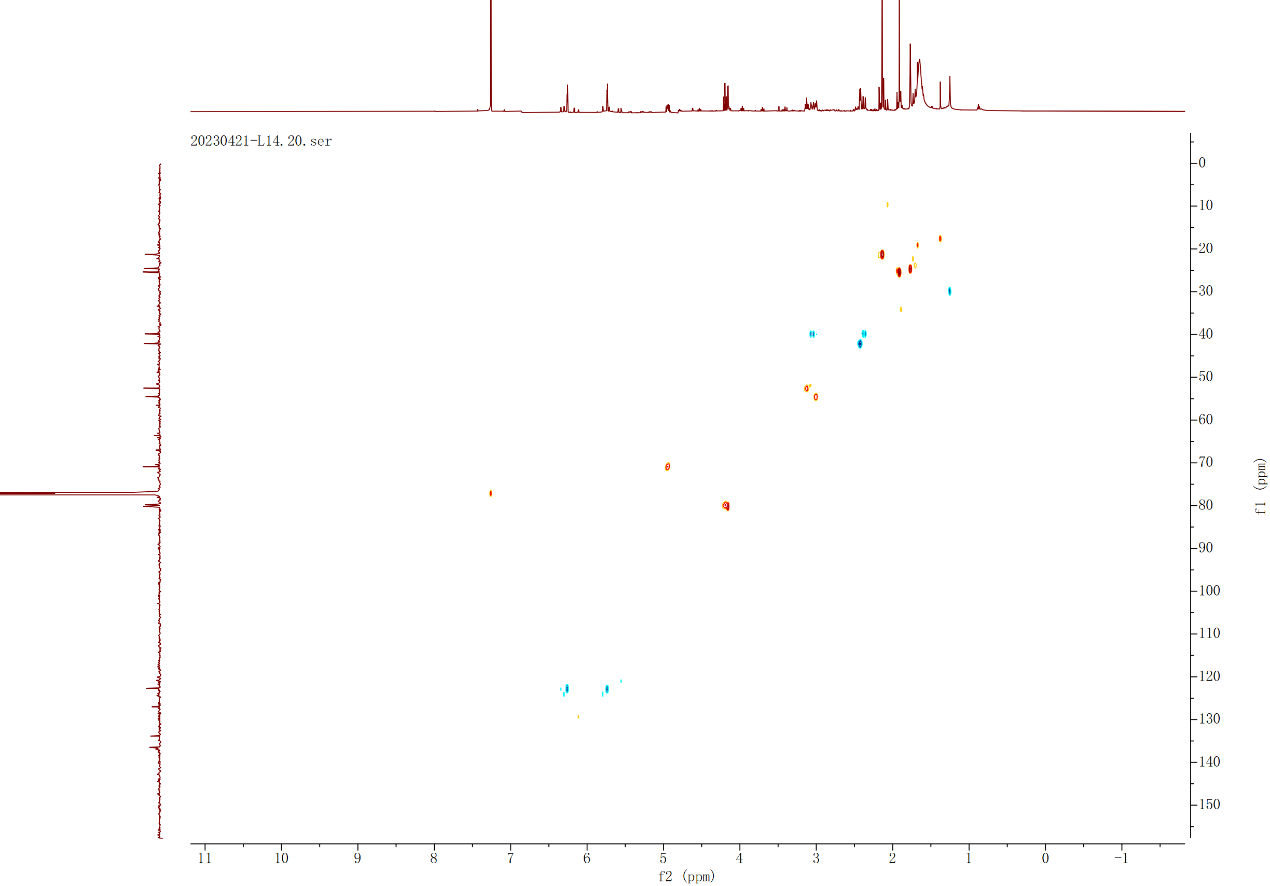


Figure S3. HSQC spectrum of compound 1 (600 MHz, CDCl_3_)


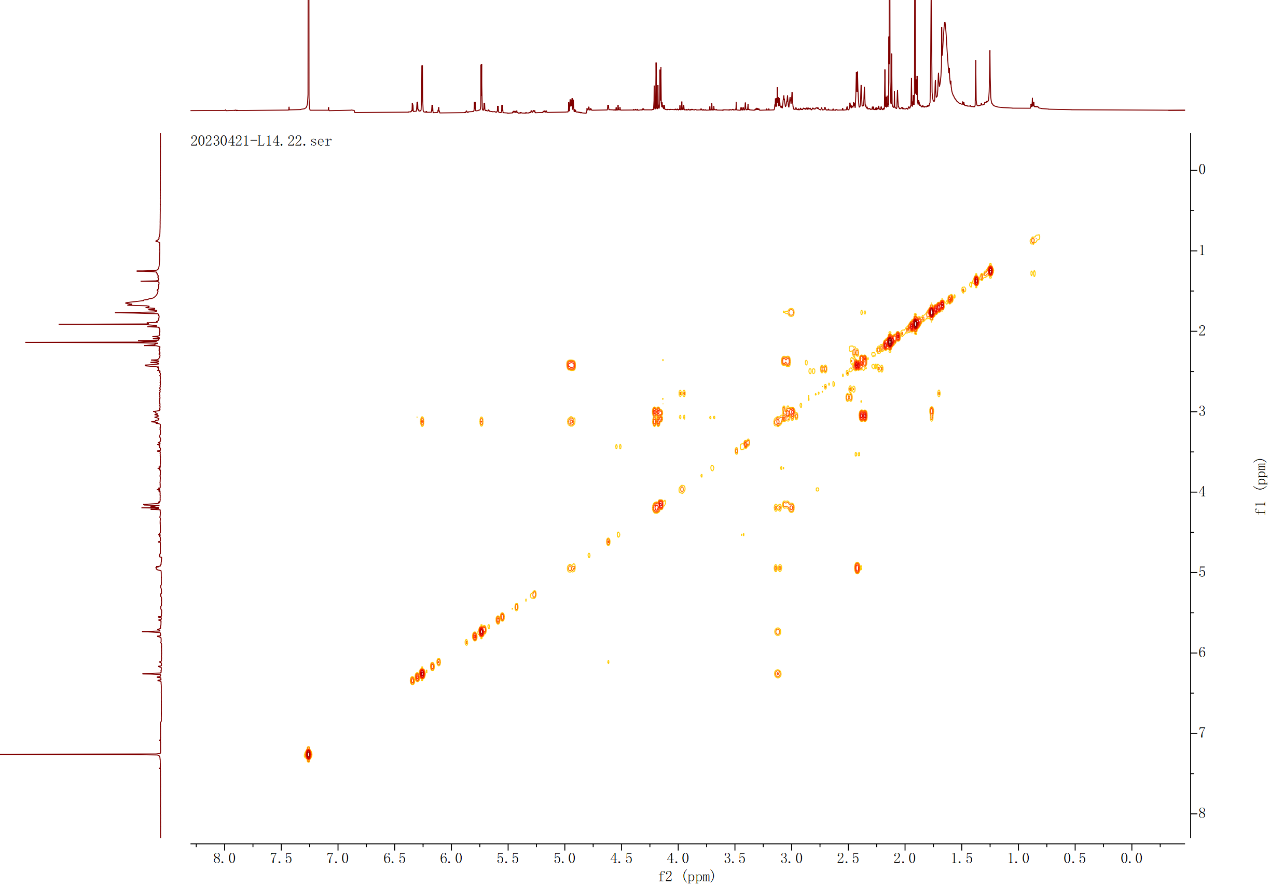


Figure S4. ^1^H-^1^H COSY spectrum of compound 1 (600 MHz, CDCl_3_)


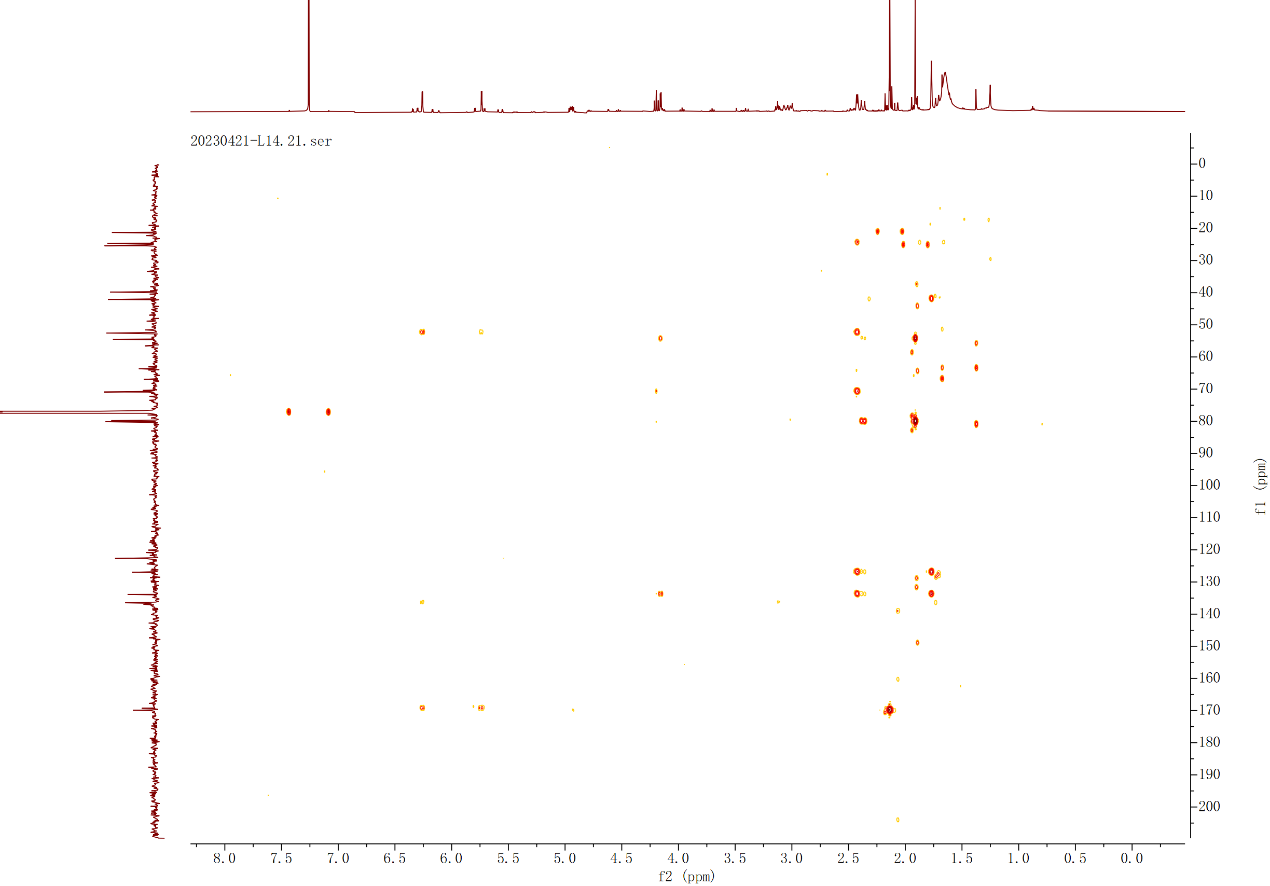


Figure S5. HMBC spectrum of compound 1 (600 MHz, CDCl_3_)


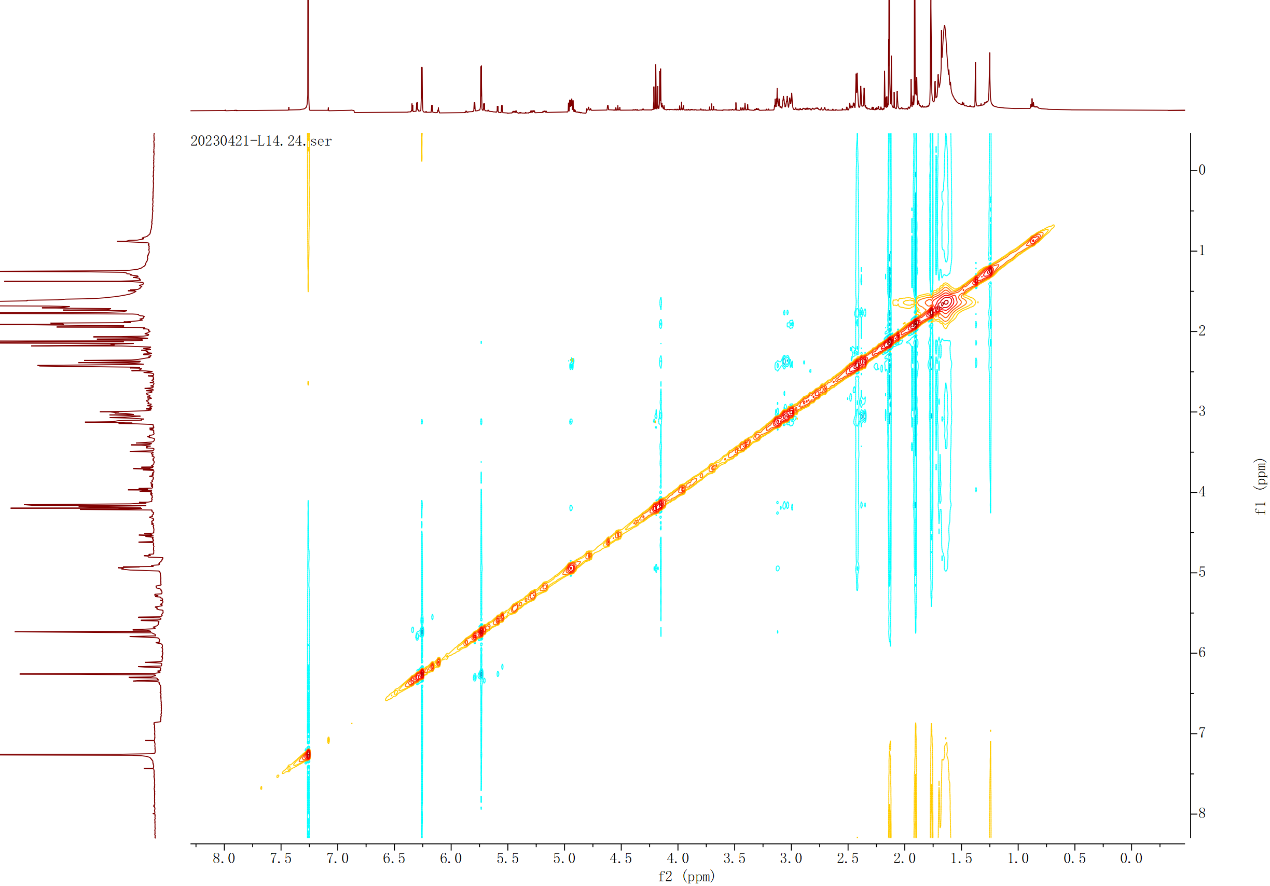


Figure S6. NOESY spectrum of compound 1 (600 MHz, CDCl_3_)


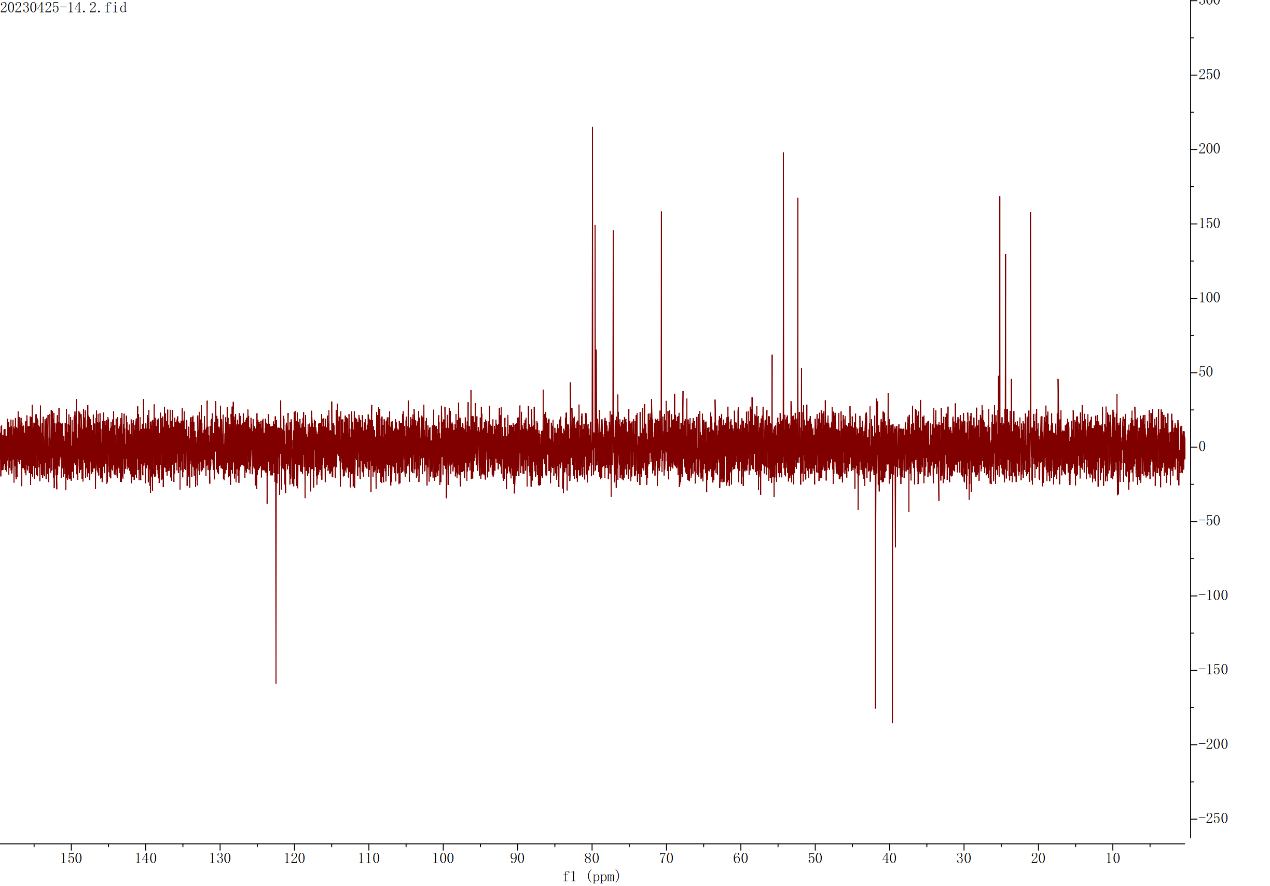


Figure S7. DEPT135 spectrum of compound 1（150 MHz, CDCl_3_）

Figure S8. HRESIMS spectrum of compound 1


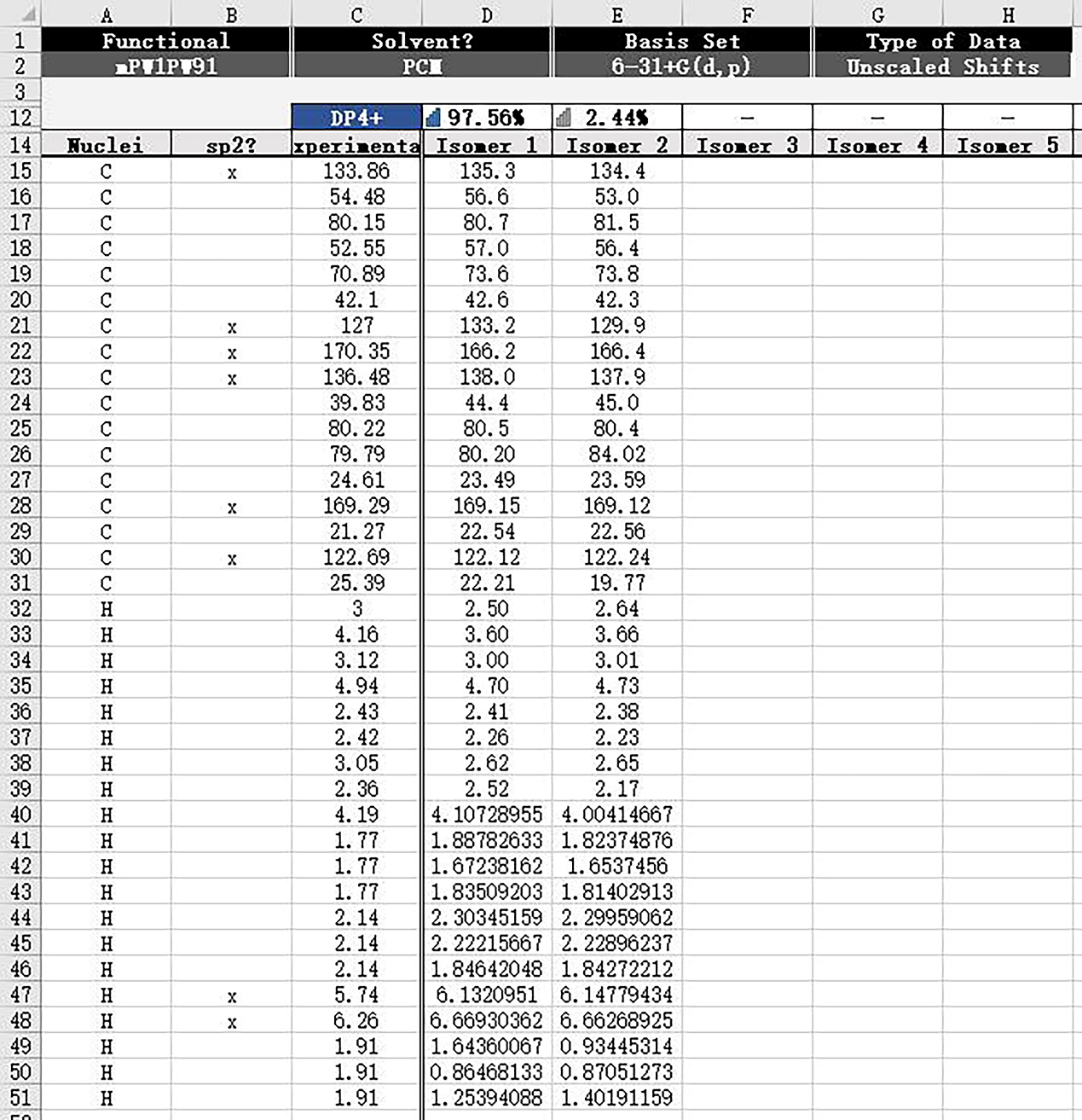


Figure S9. DP4+ result of compound 1


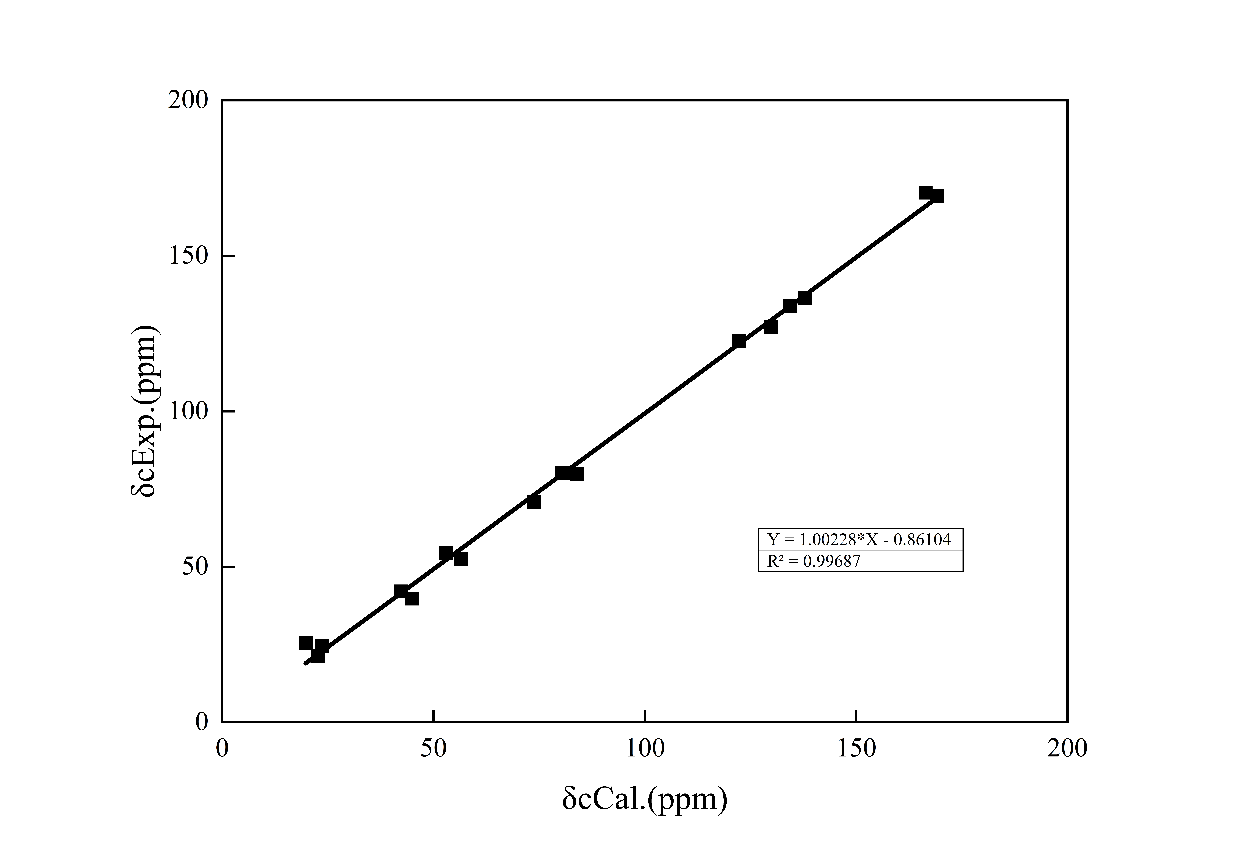


Figure S10. NMR fitted curves of compound 1


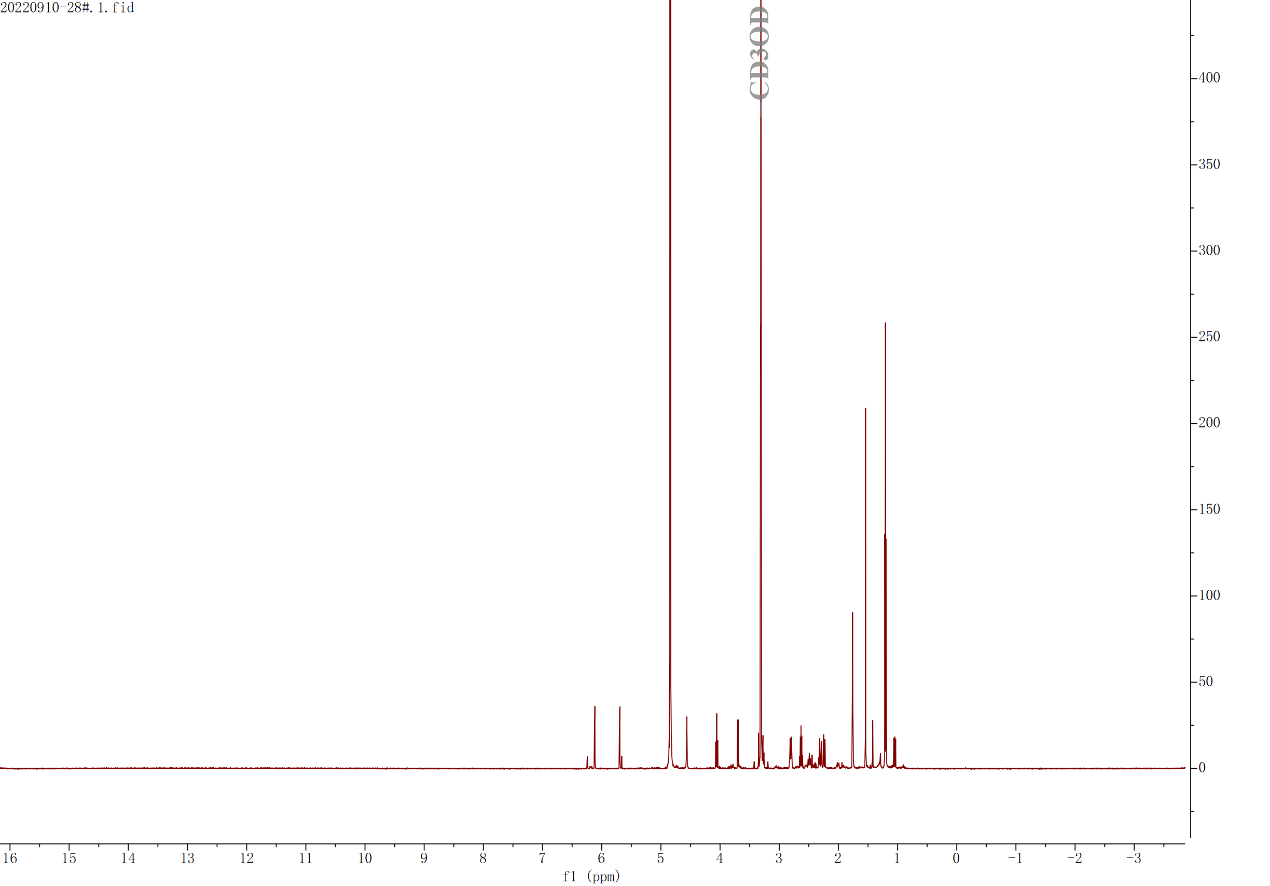


Figure S11. ^1^H-NMR spectrum of compound 2（600 MHz, CD_3_OD）


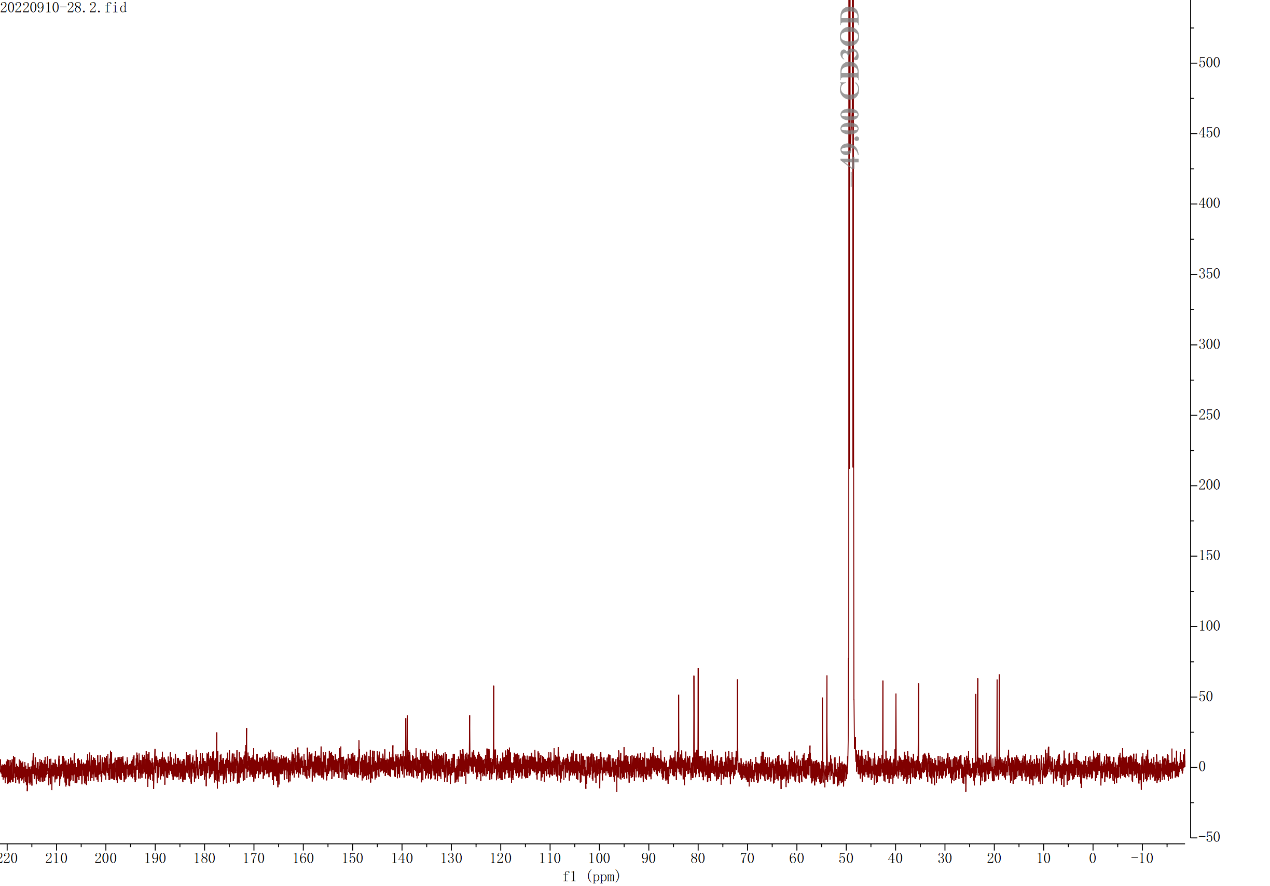


Figure S12. ^13^C-NMR spectrum of compound 2（150 MHz, CD_3_OD）


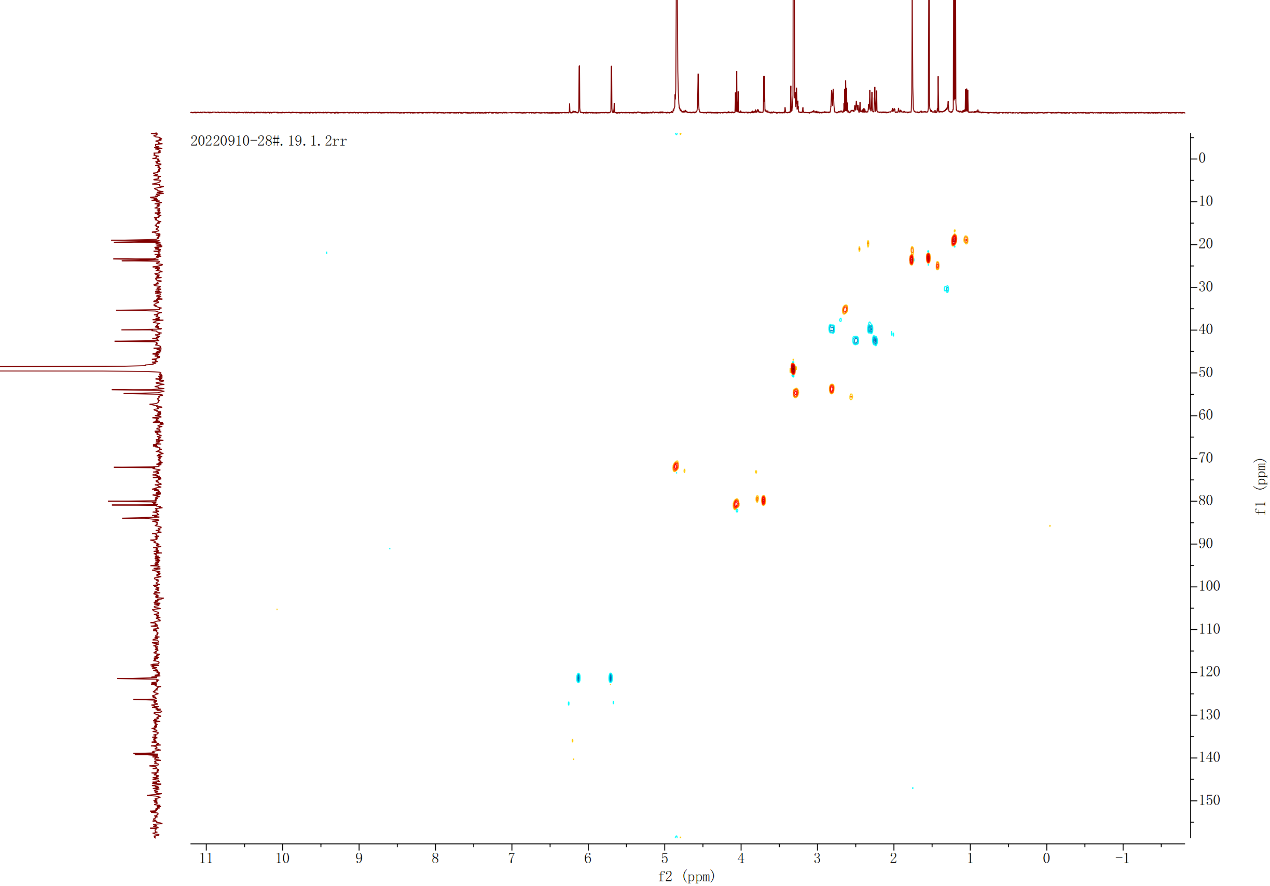


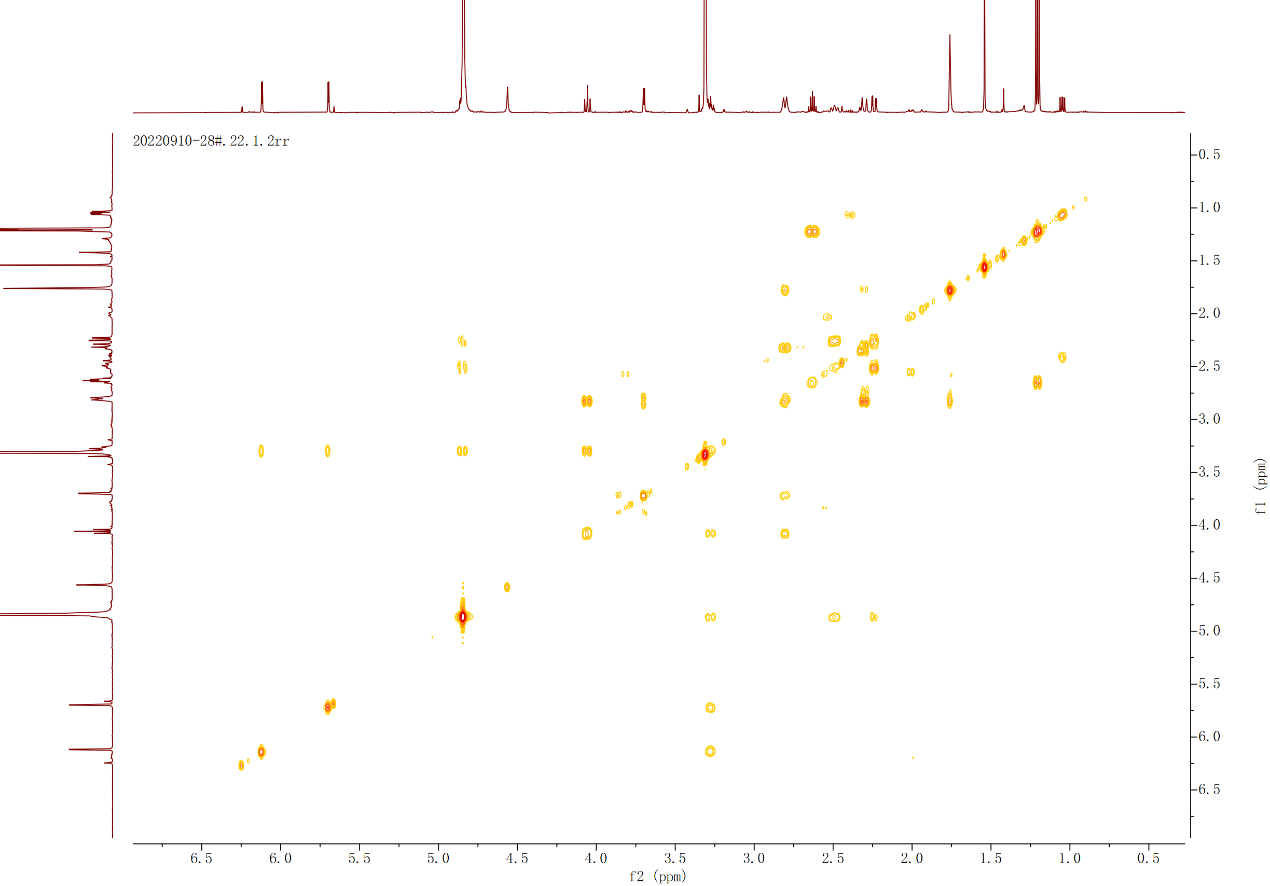
Figure S13. HSQC spectrum of compound 2（600 MHz, CD_3_OD）

Figure S14. ^1^H-^1^H COSY spectrum of compound 2（600 MHz, CD_3_OD）


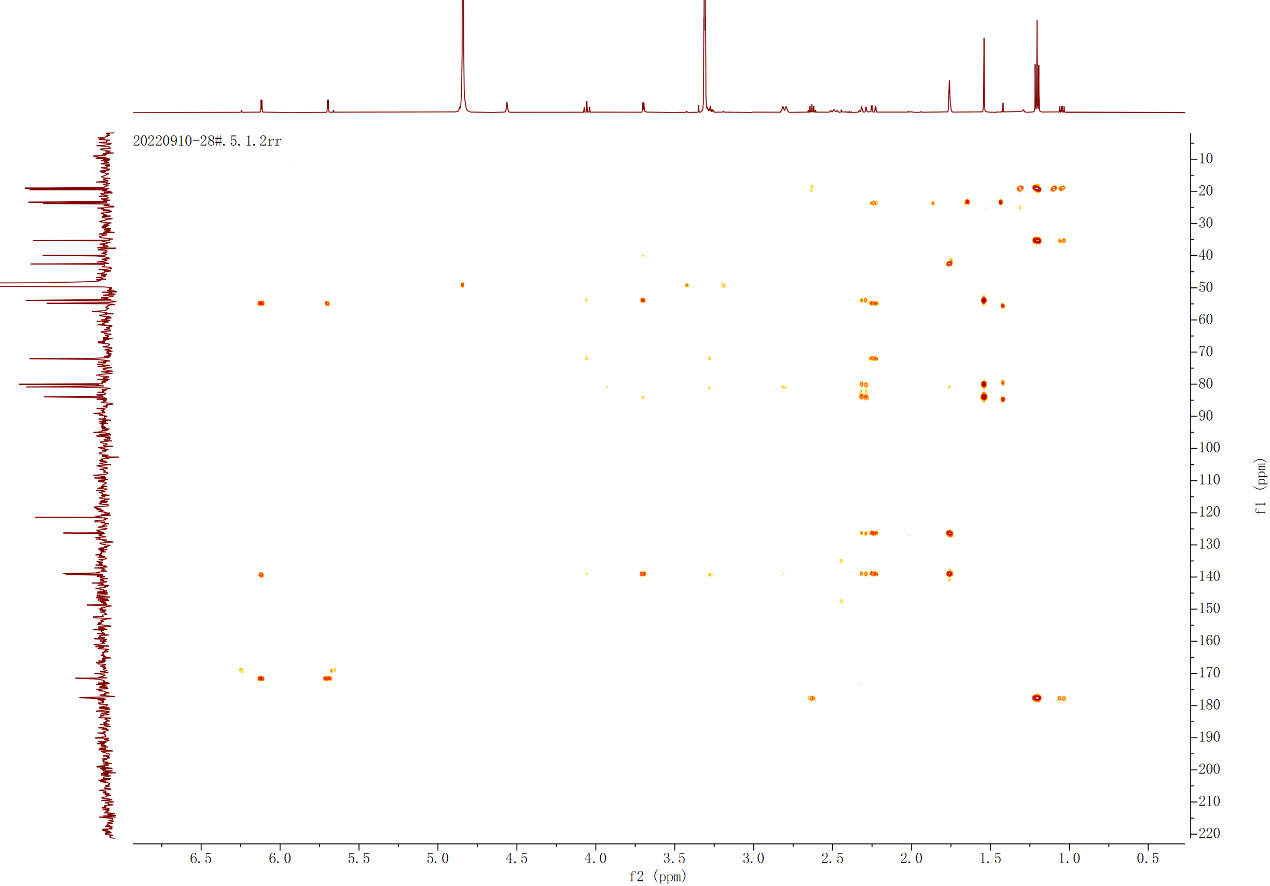


Figure S15. HMBC spectrum of compound 2（600 MHz, CD_3_OD）


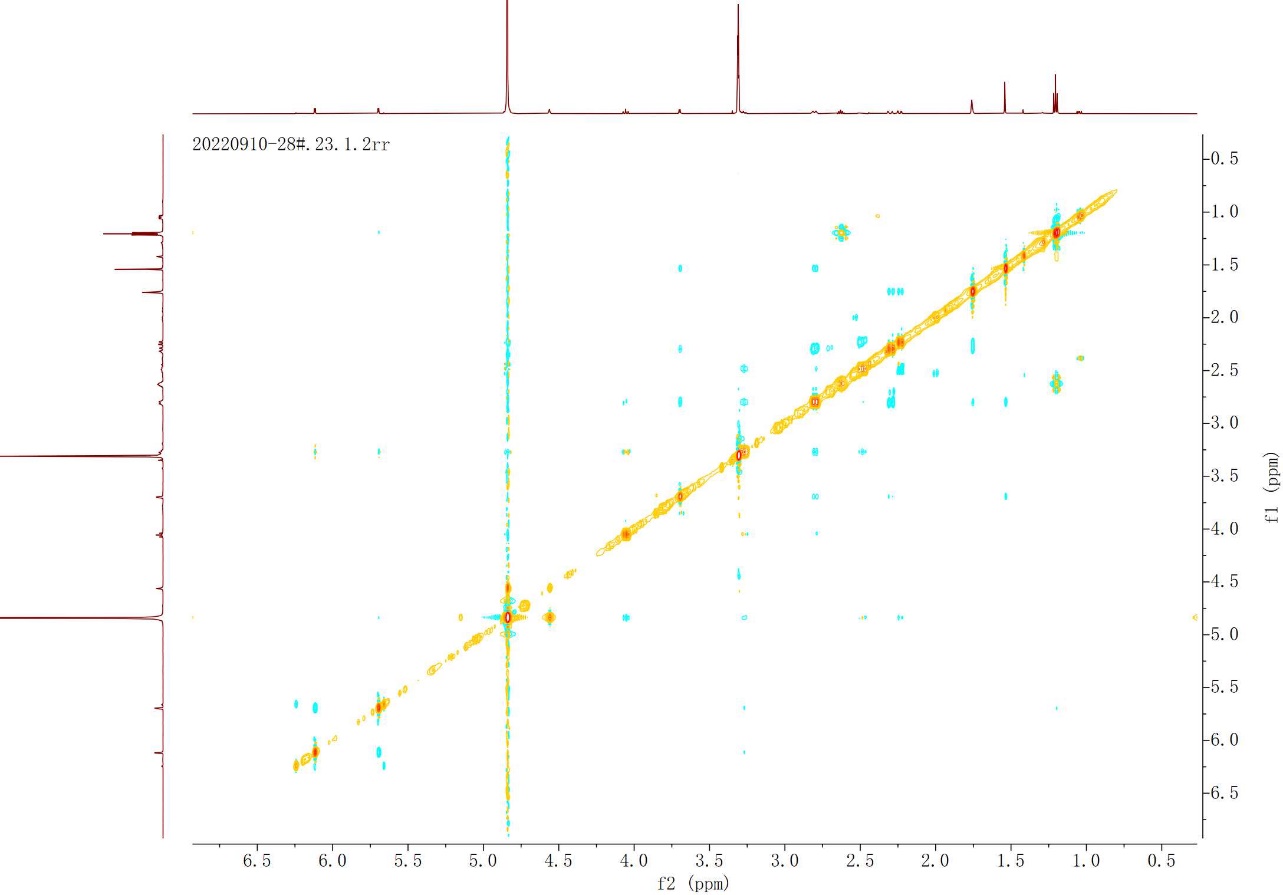


Figure S16. NOESY spectrum of compound 2（600 MHz, CD_3_OD）


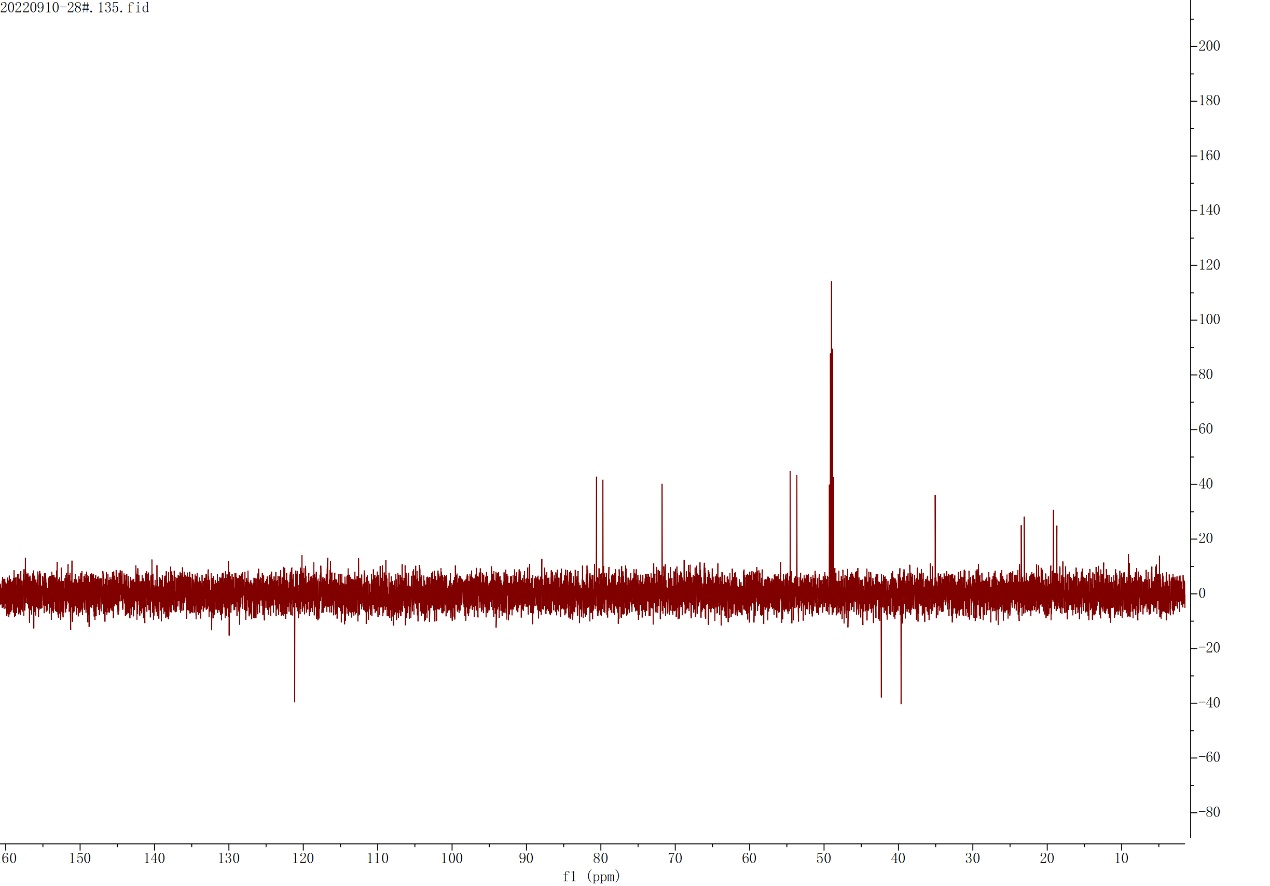


Figure S17. DEPT135 spectrum of compound 2（150 MHz, CD_3_OD）

Figure S18. HRESIMS spectrum of compound 2


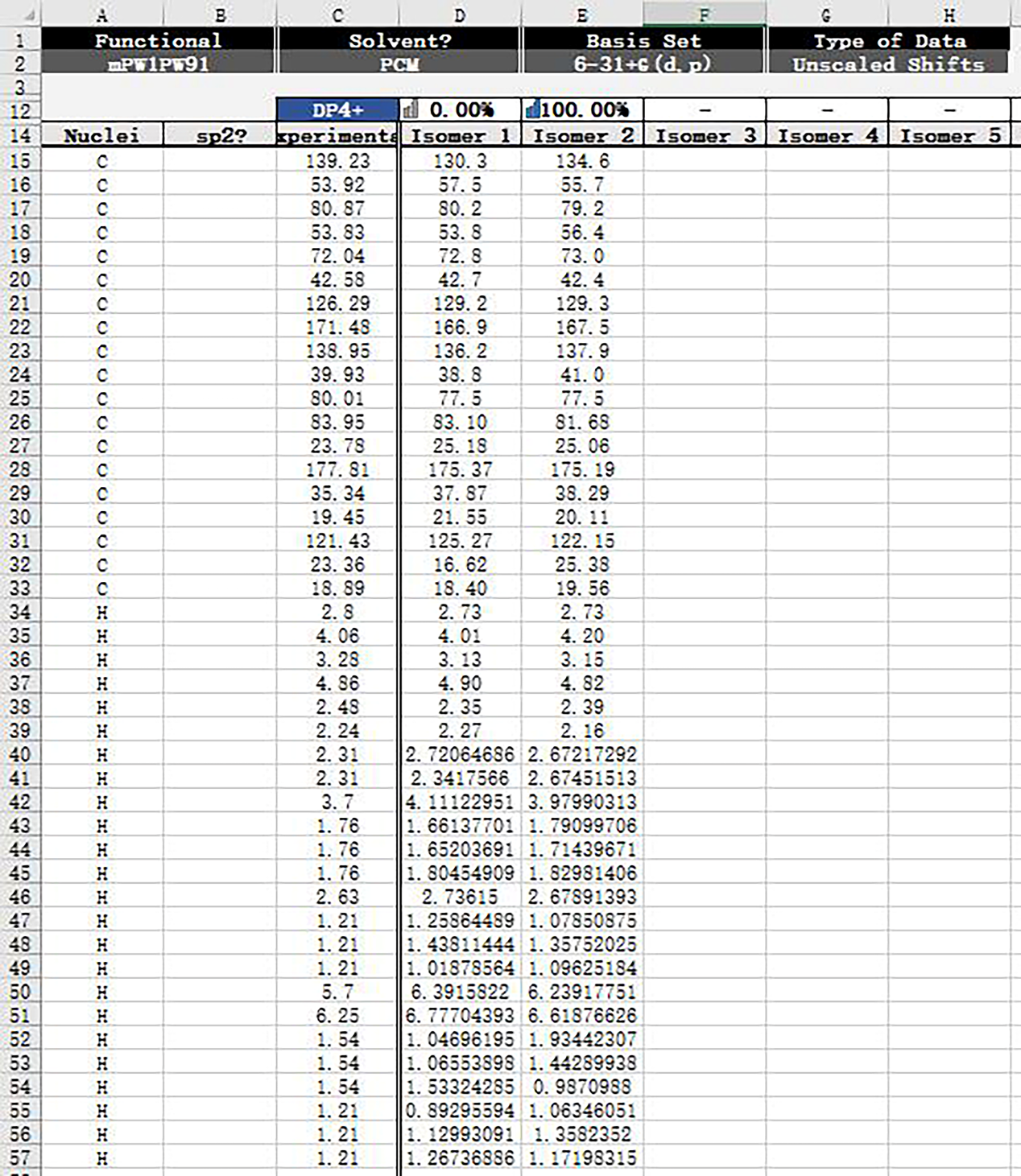


Figure S19. DP4+ result of compound 2


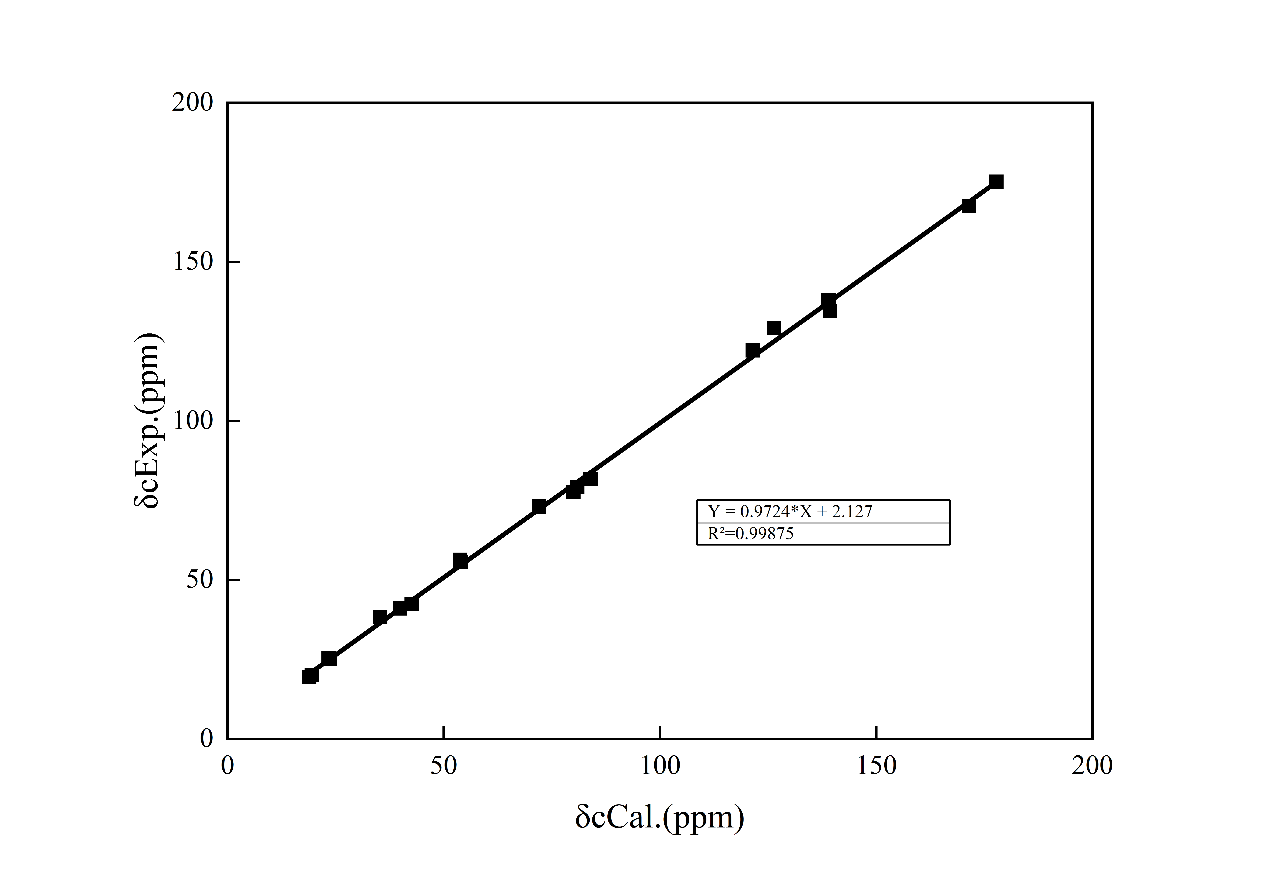


Figure S20. NMR fitted curves of compound 2


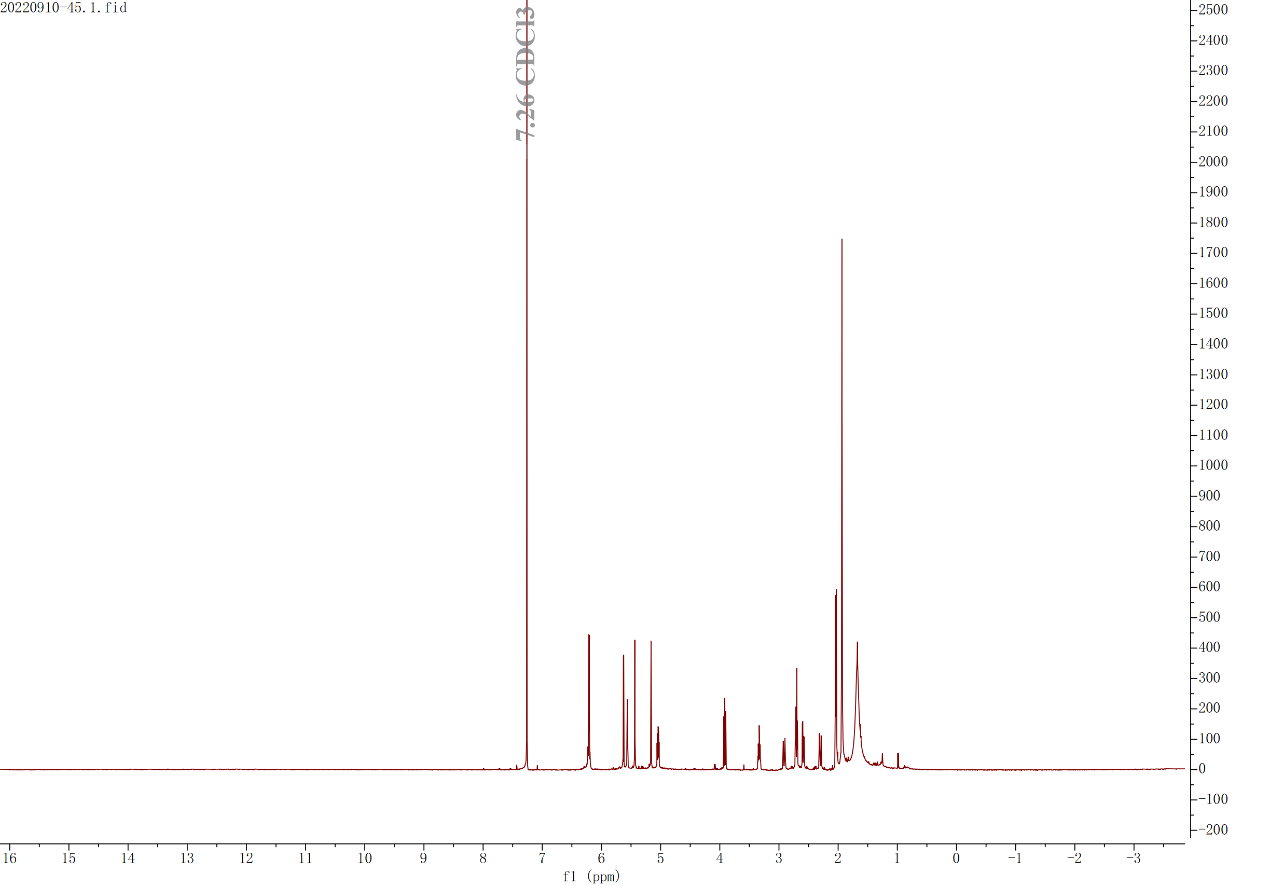


Figure S21. ^1^H-NMR spectrum of compound 3（600 MHz, CDCl_3_）


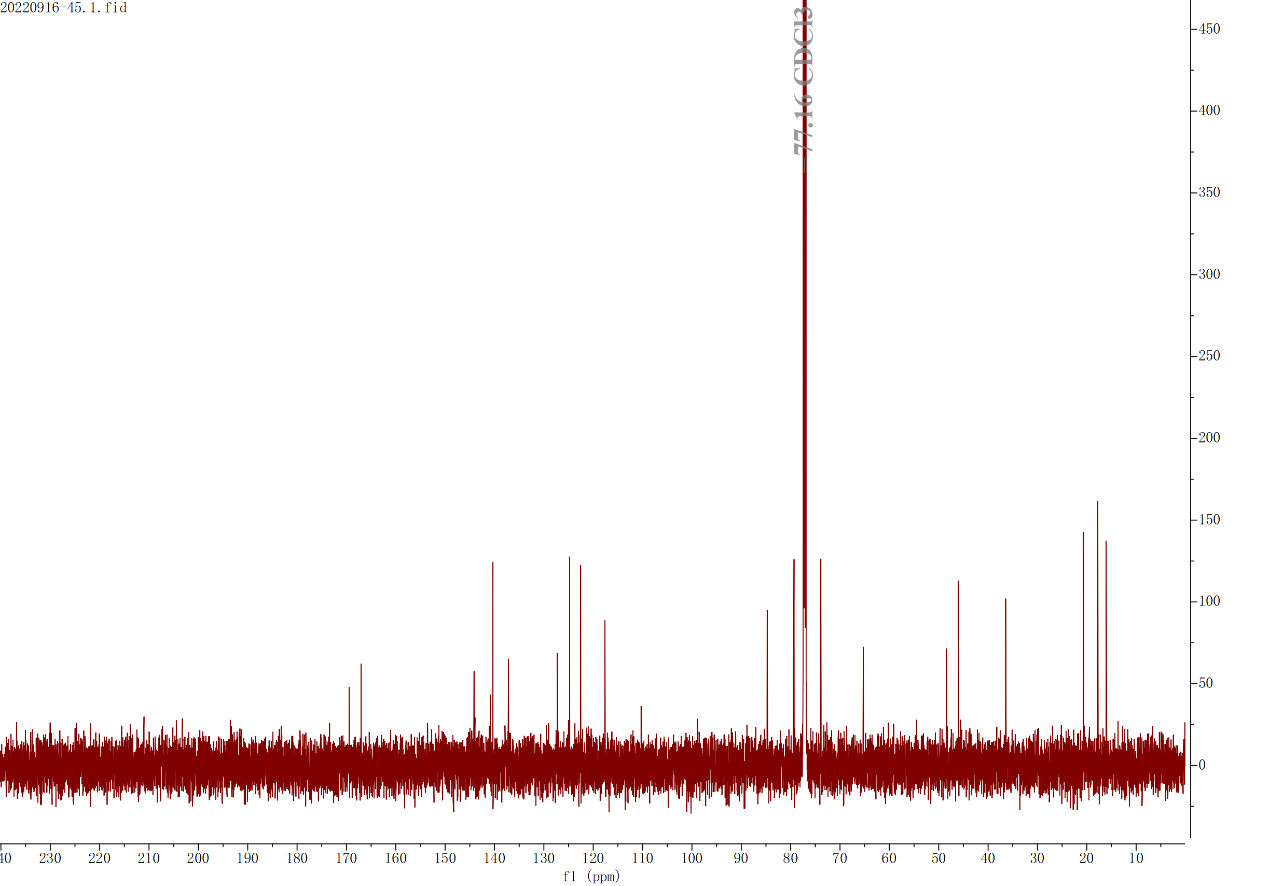


Figure S22. ^13^C-NMR spectrum of compound 3（150 MHz, CDCl_3_）


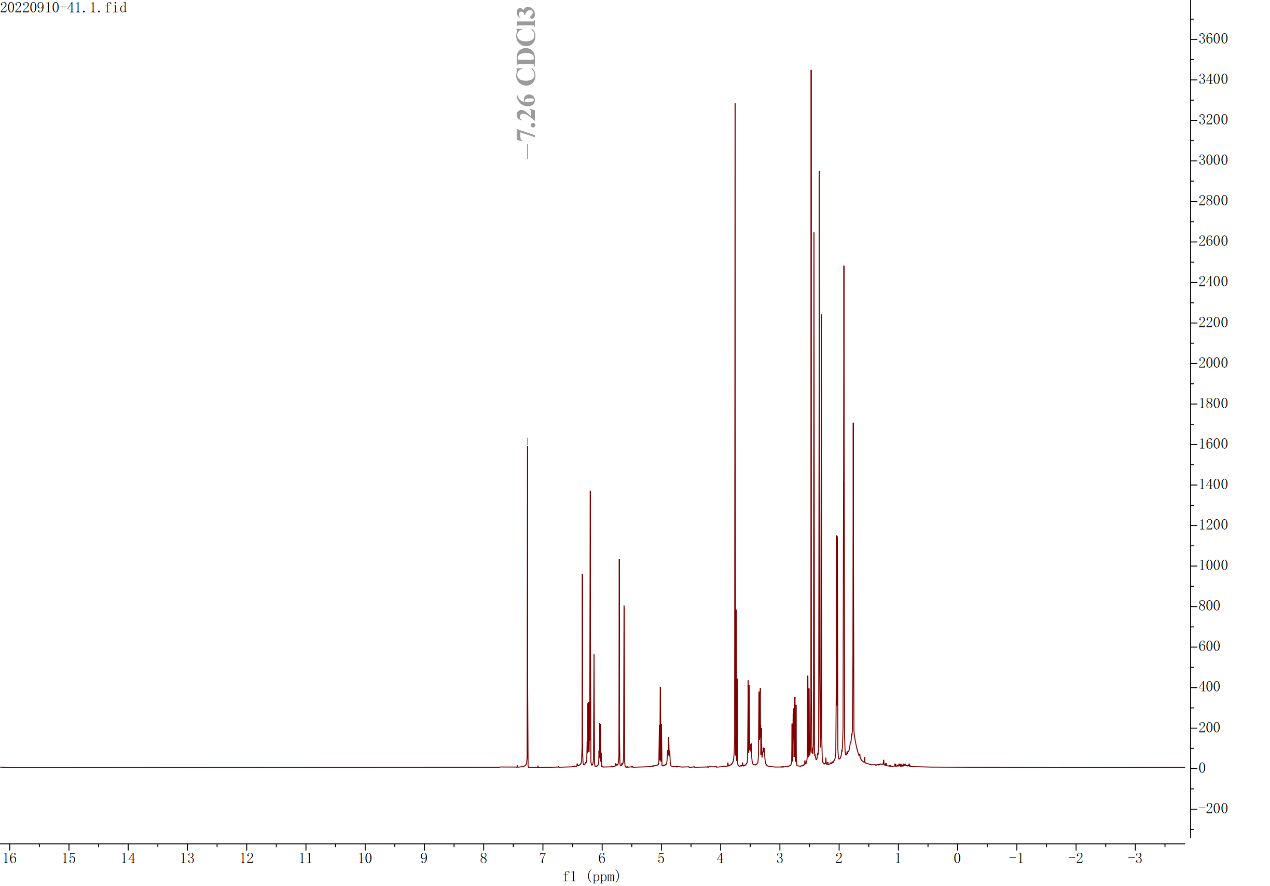


Figure S23. ^1^H-NMR spectrum of compound 4（600 MHz, CDCl_3_）


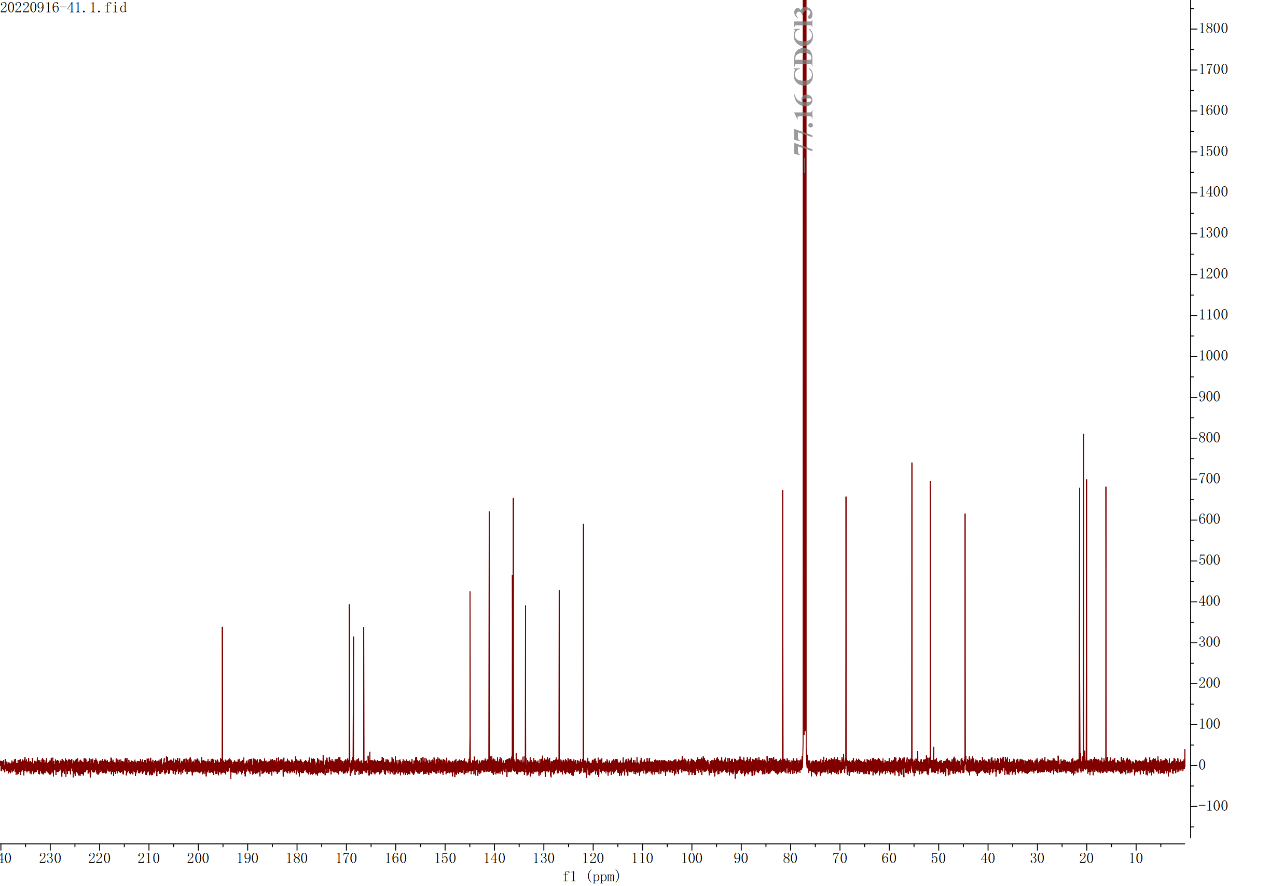


Figure S24. ^13^C-NMR spectrum of compound 4（150 MHz, CDCl_3_）


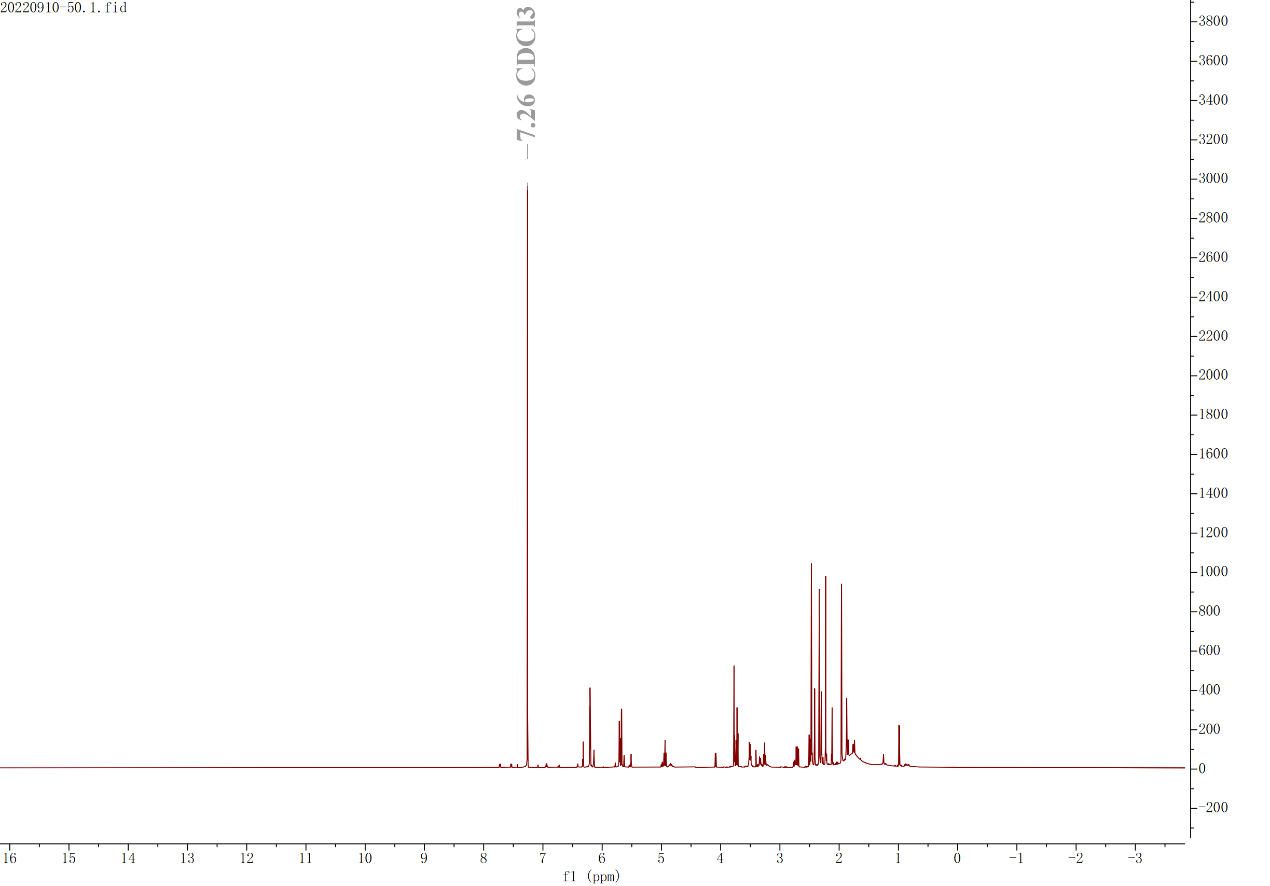


Figure S25. ^1^H-NMR spectrum of compound 5（600 MHz, CDCl_3_）


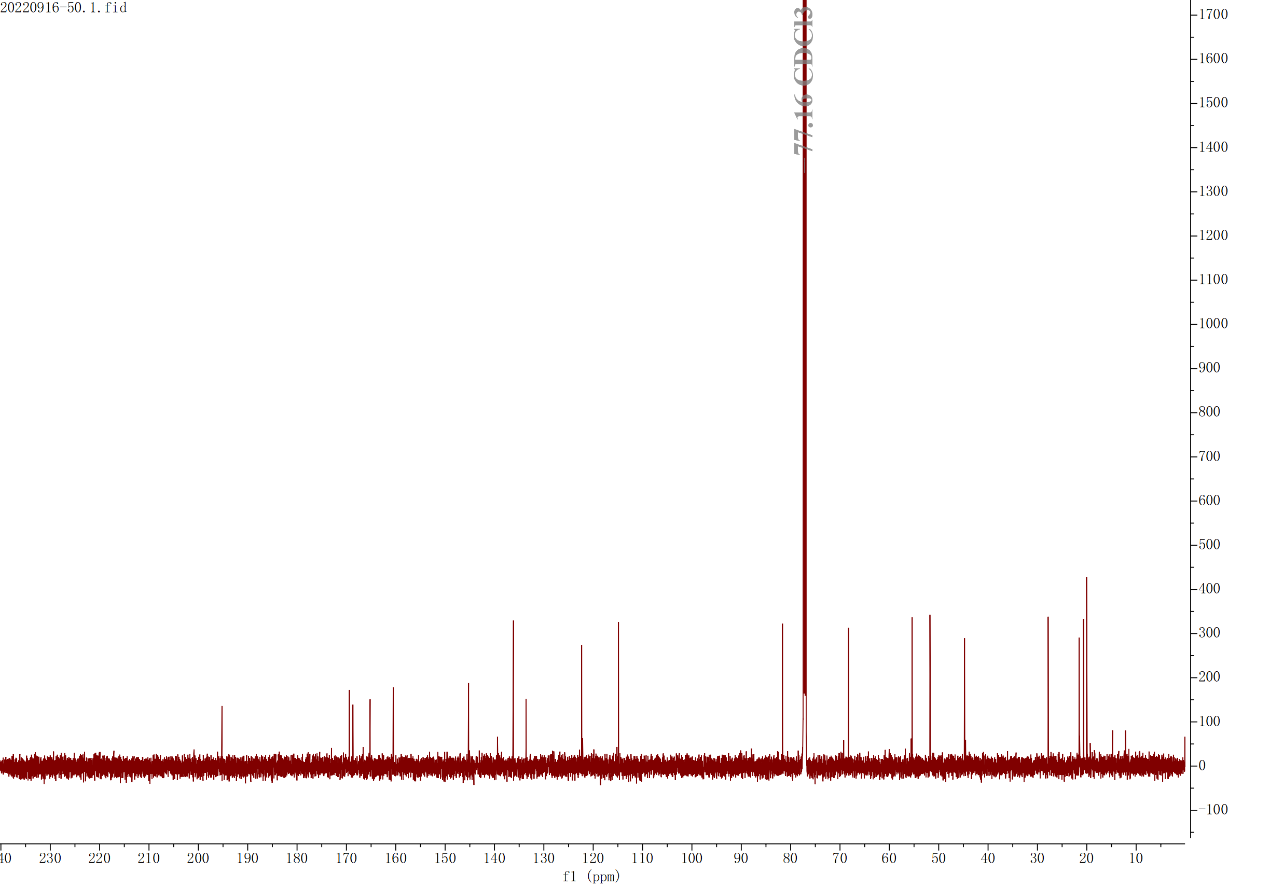


Figure S26. ^13^C-NMR spectrum of compound 5（150 MHz, CDCl_3_）


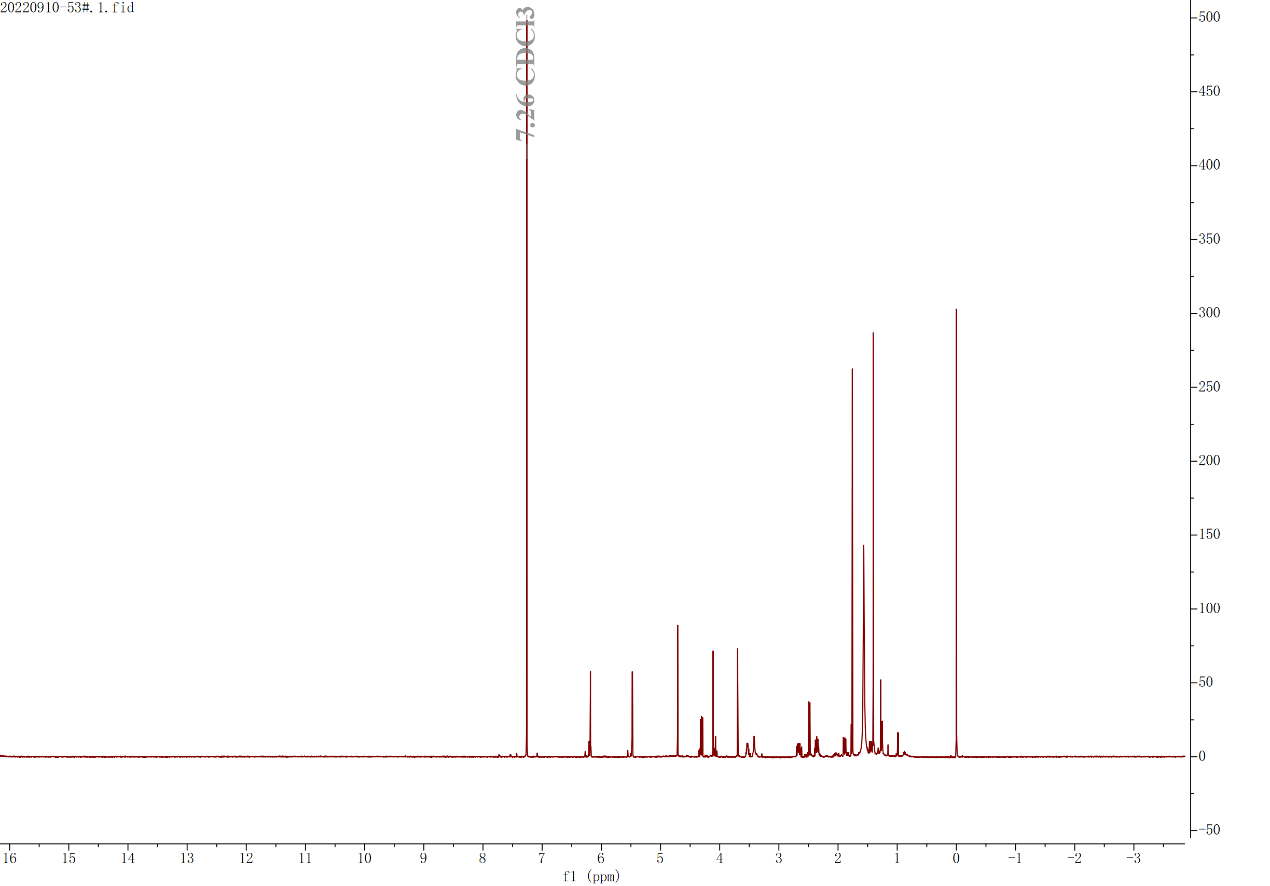


Figure S27. ^1^H-NMR spectrum of compound 6（600 MHz, CDCl_3_）


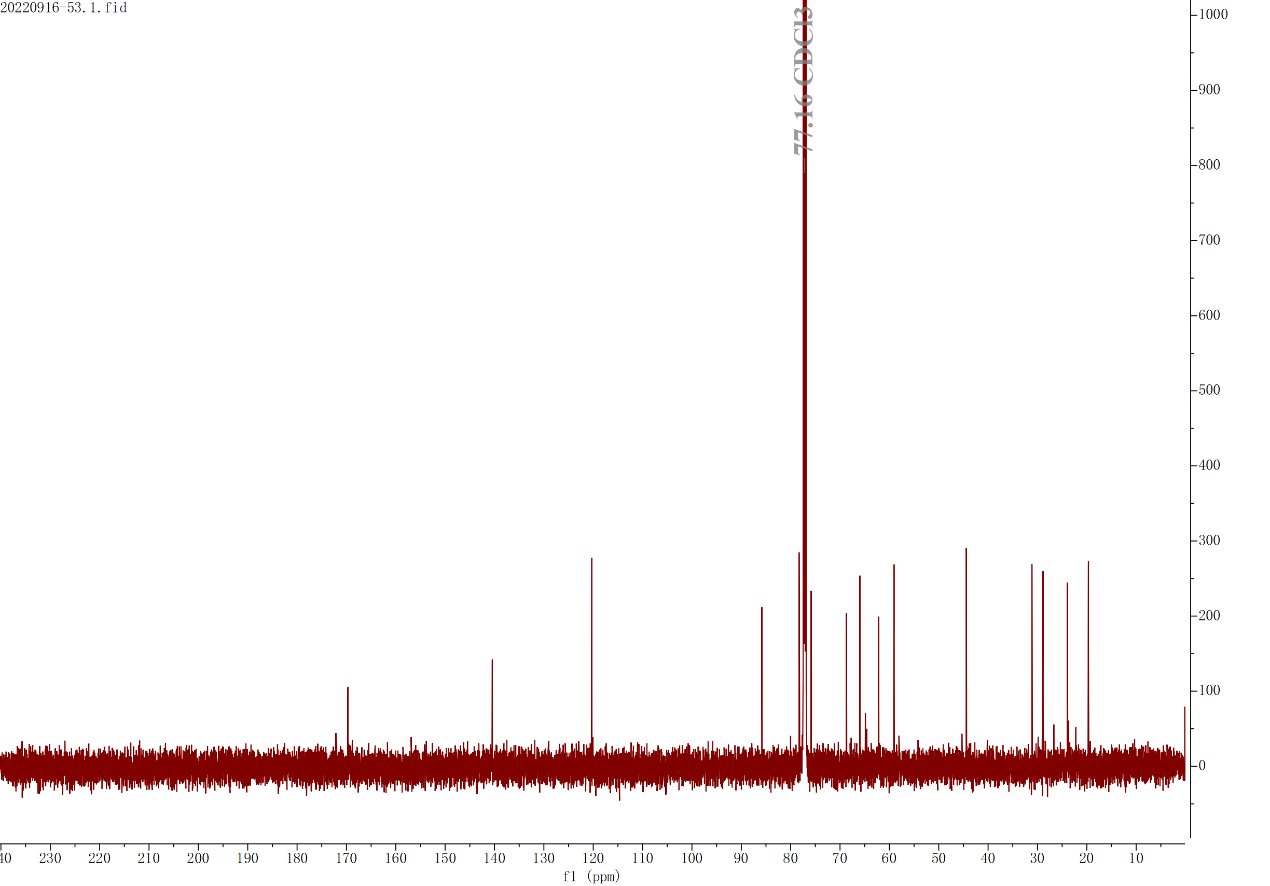


Figure S28. ^13^C-NMR spectrum of compound 6（150 MHz, CDCl_3_）


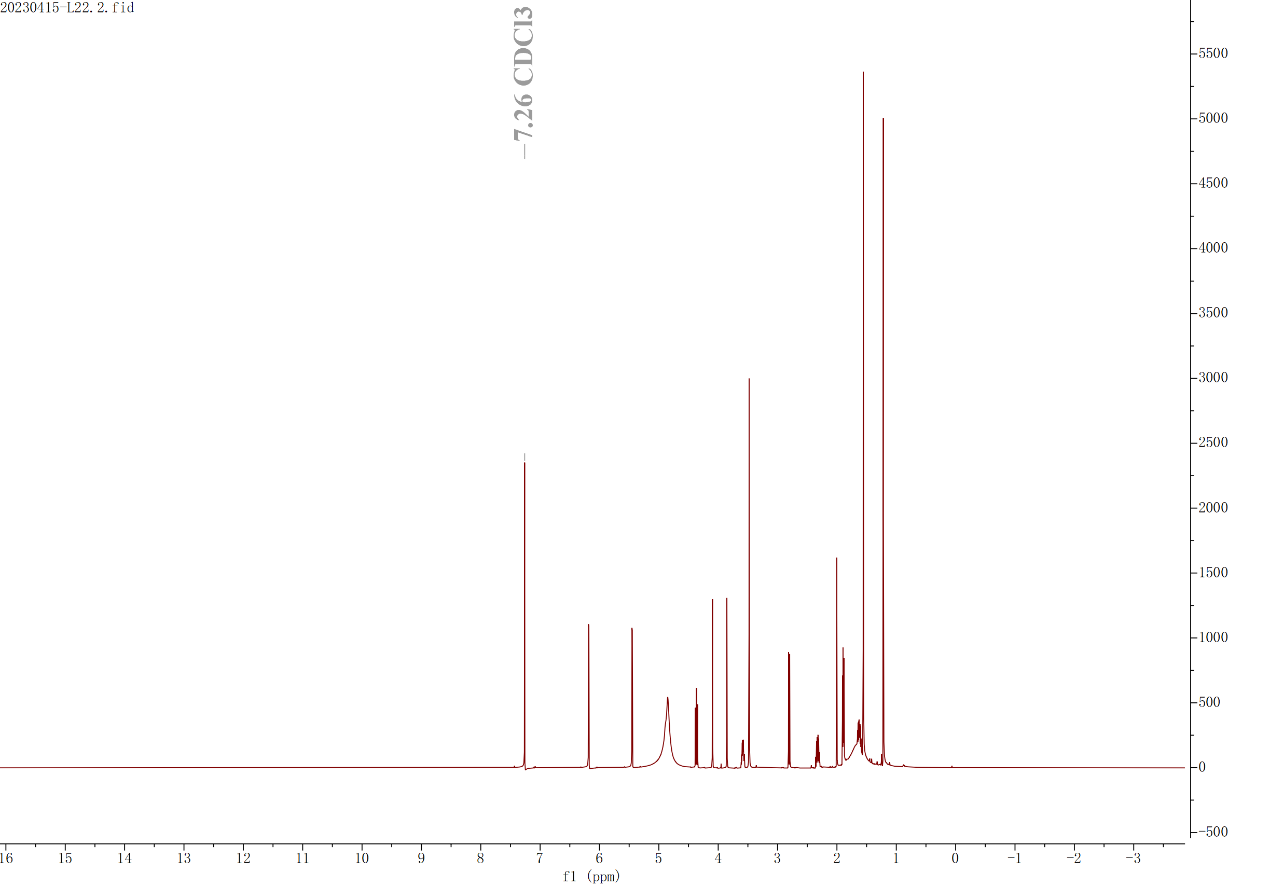


Figure S29. ^1^H-NMR spectrum of compound 7（600 MHz, CDCl_3_）


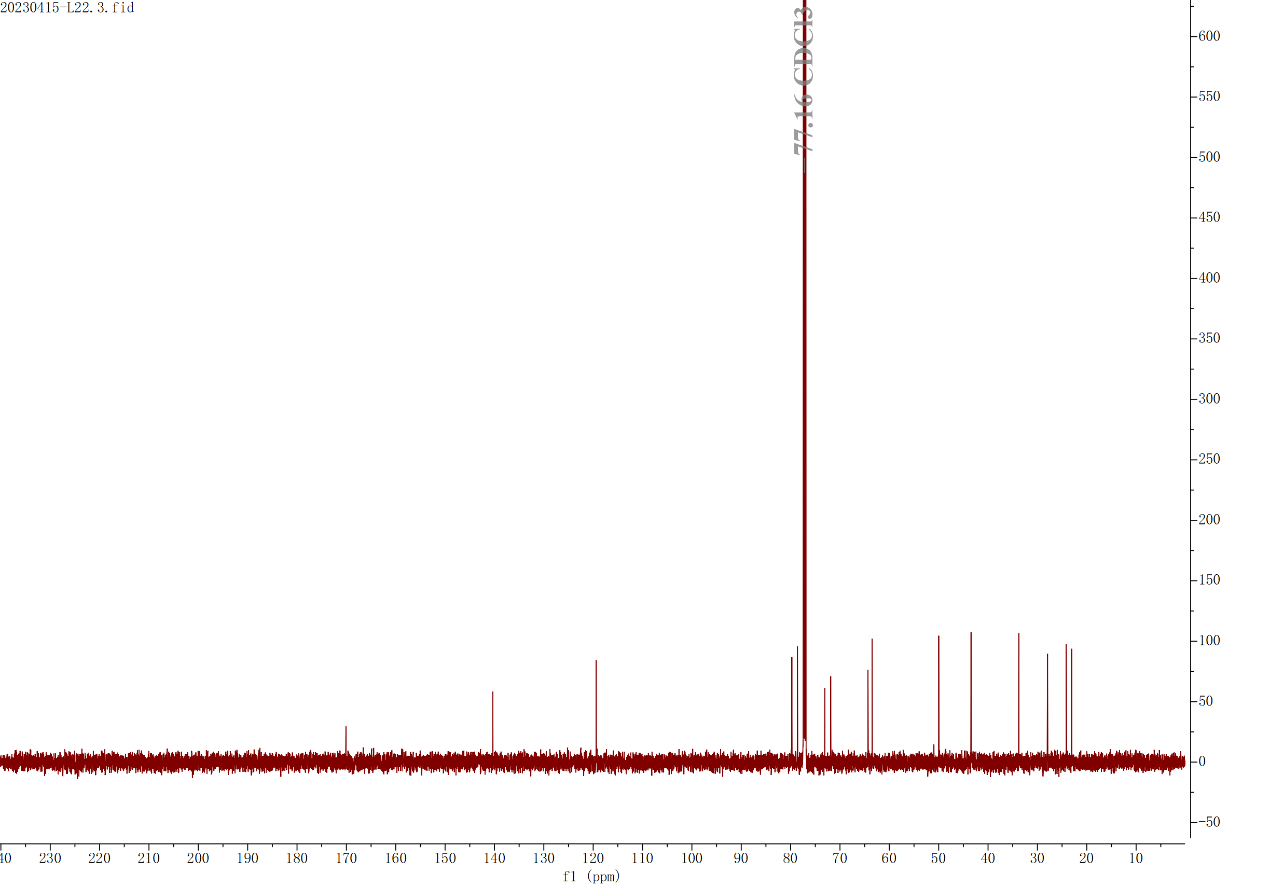


Figure S30. ^13^C-NMR spectrum of compound 7（150 MHz, CDCl_3_）


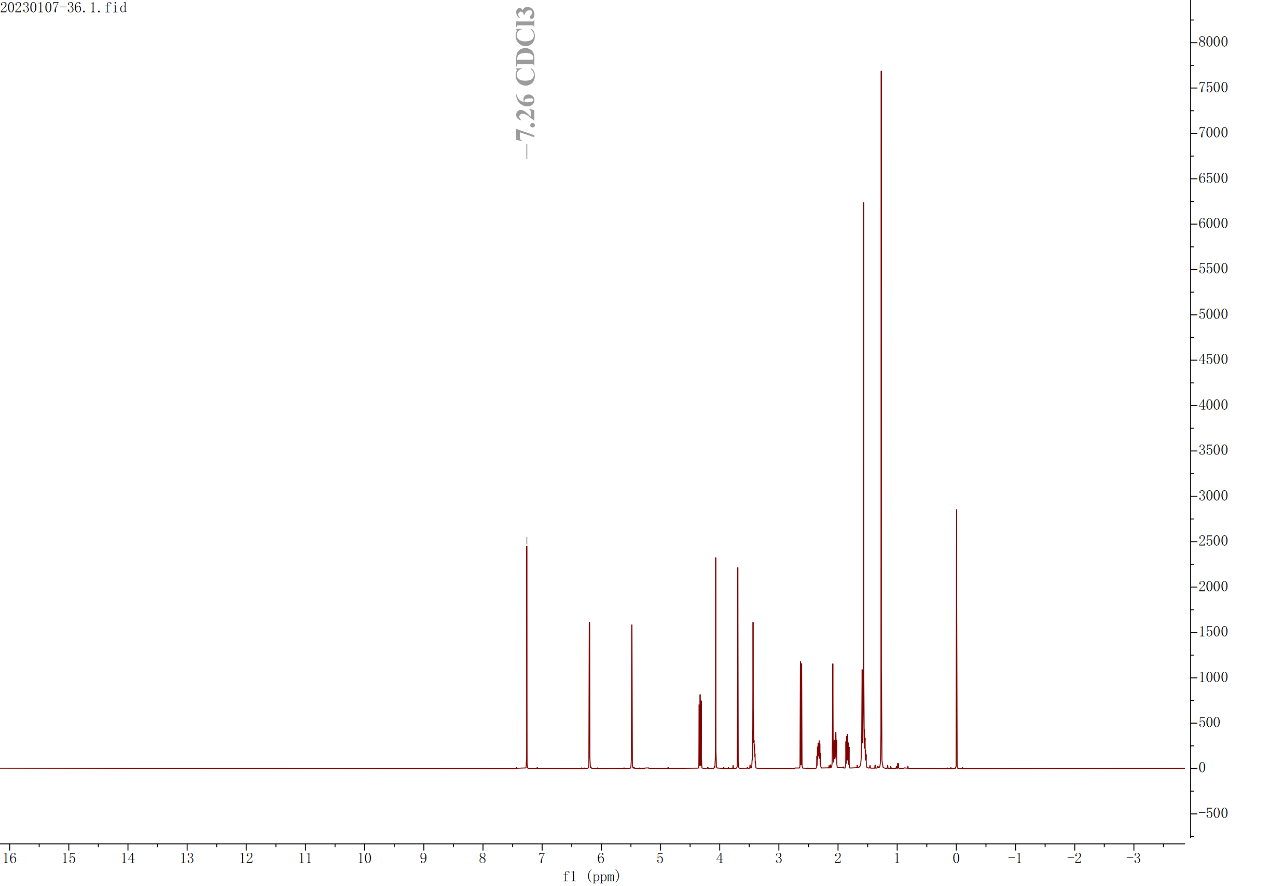


Figure S31. ^1^H-NMR spectrum of compound 8（600 MHz, CDCl_3_）


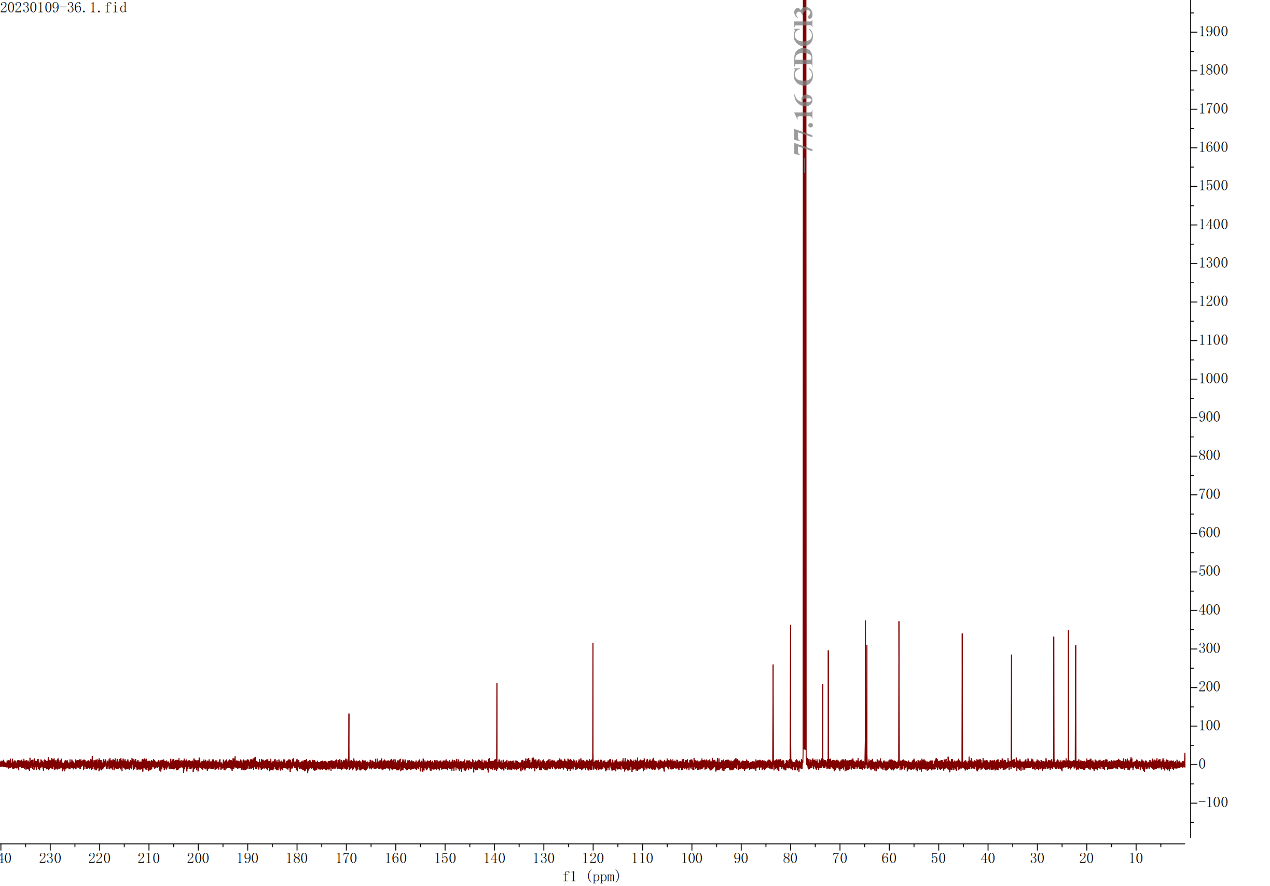


Figure S32. ^13^C-NMR spectrum of compound 8（150 MHz, CDCl_3_）


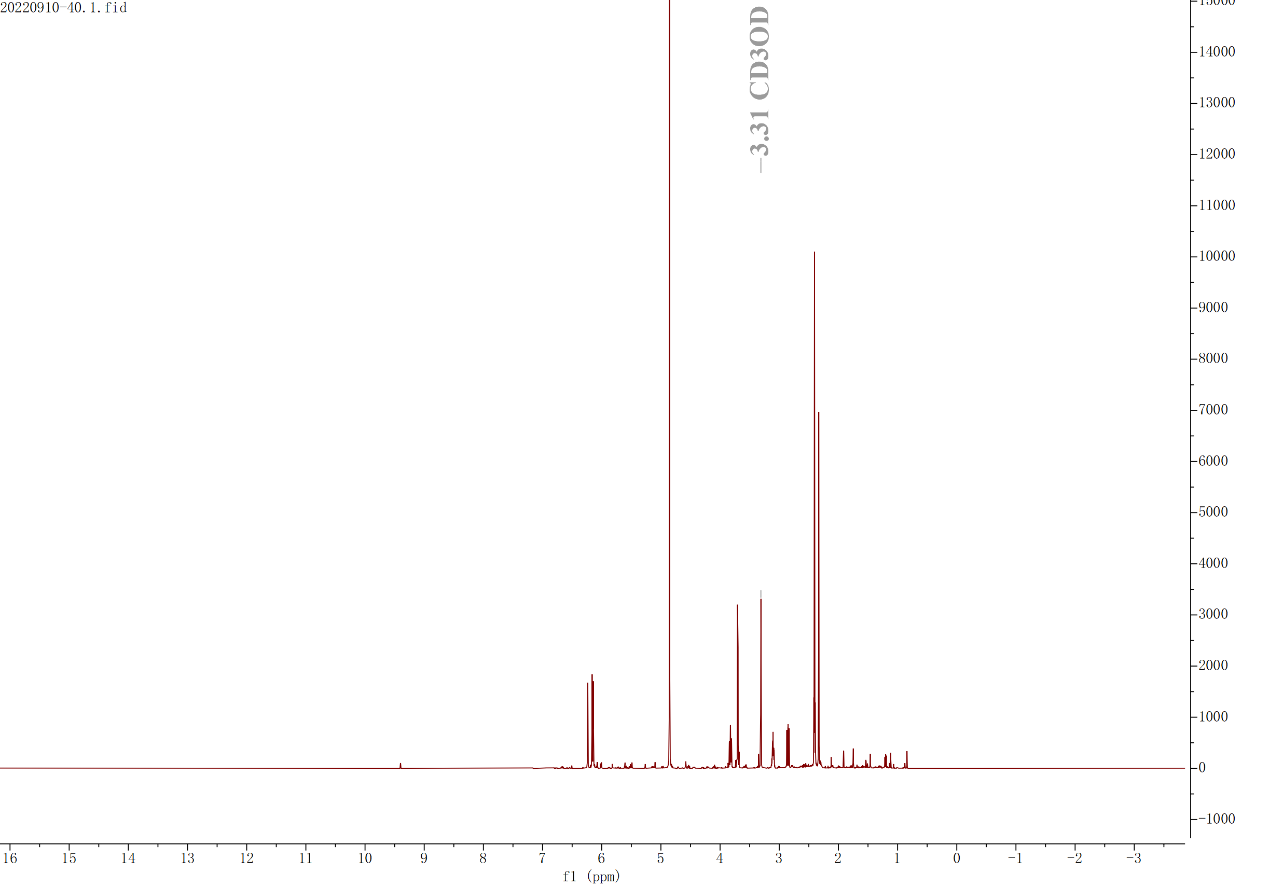


Figure S33. ^1^H-NMR spectrum of compound 9（600 MHz, CD_3_OD）


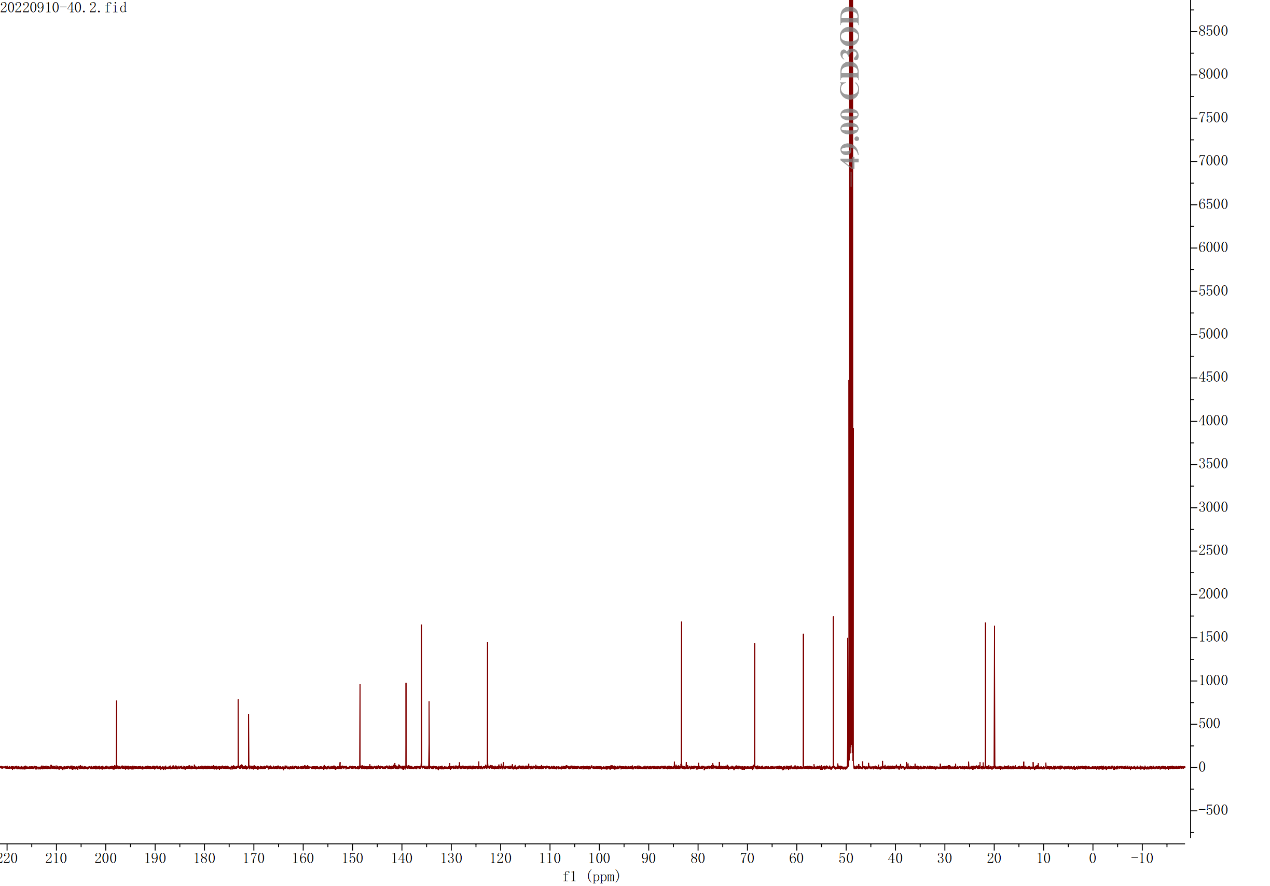


Figure S34. ^13^C-NMR spectrum of compound 9（150 MHz, CD_3_OD）


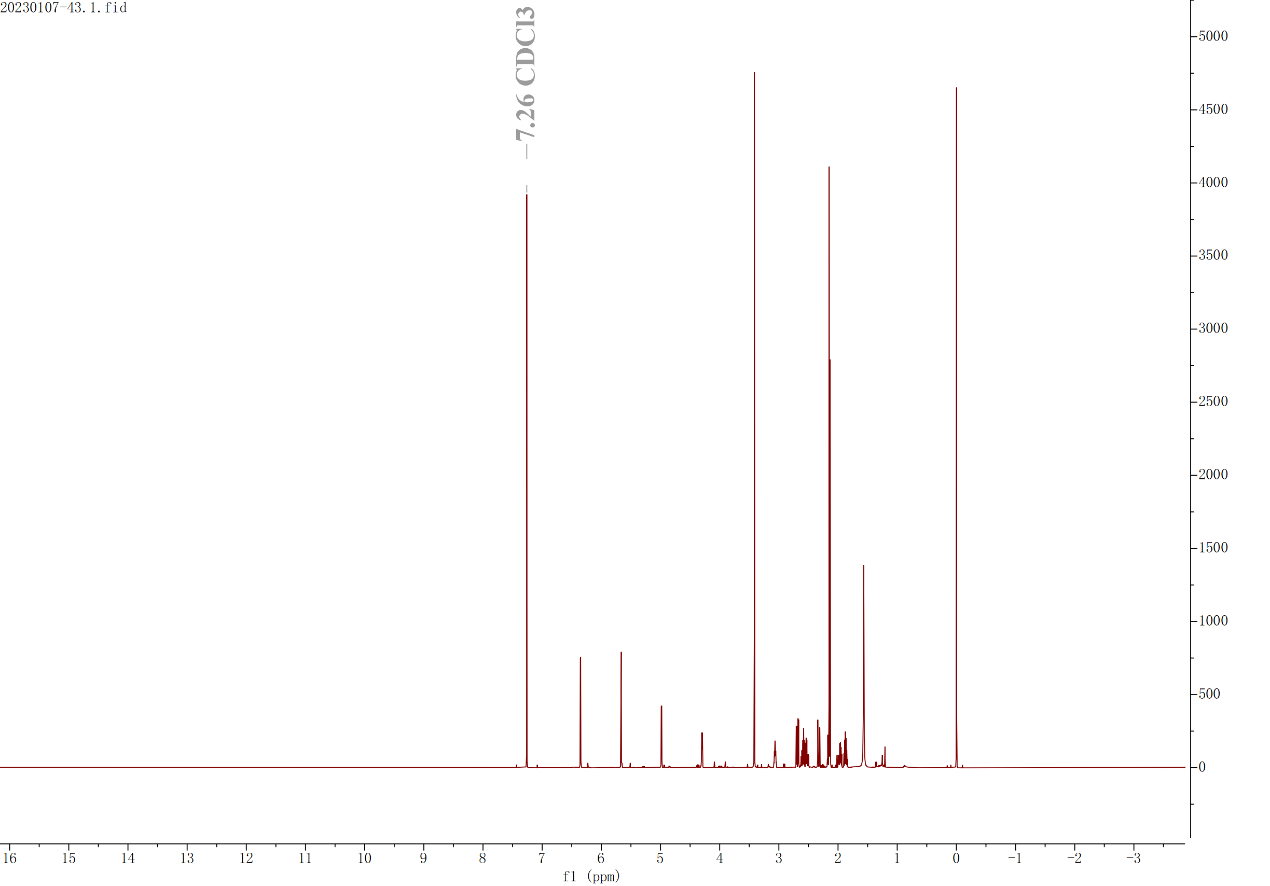


Figure S35. ^1^H-NMR spectrum of compound 10（600 MHz, CDCl_3_）


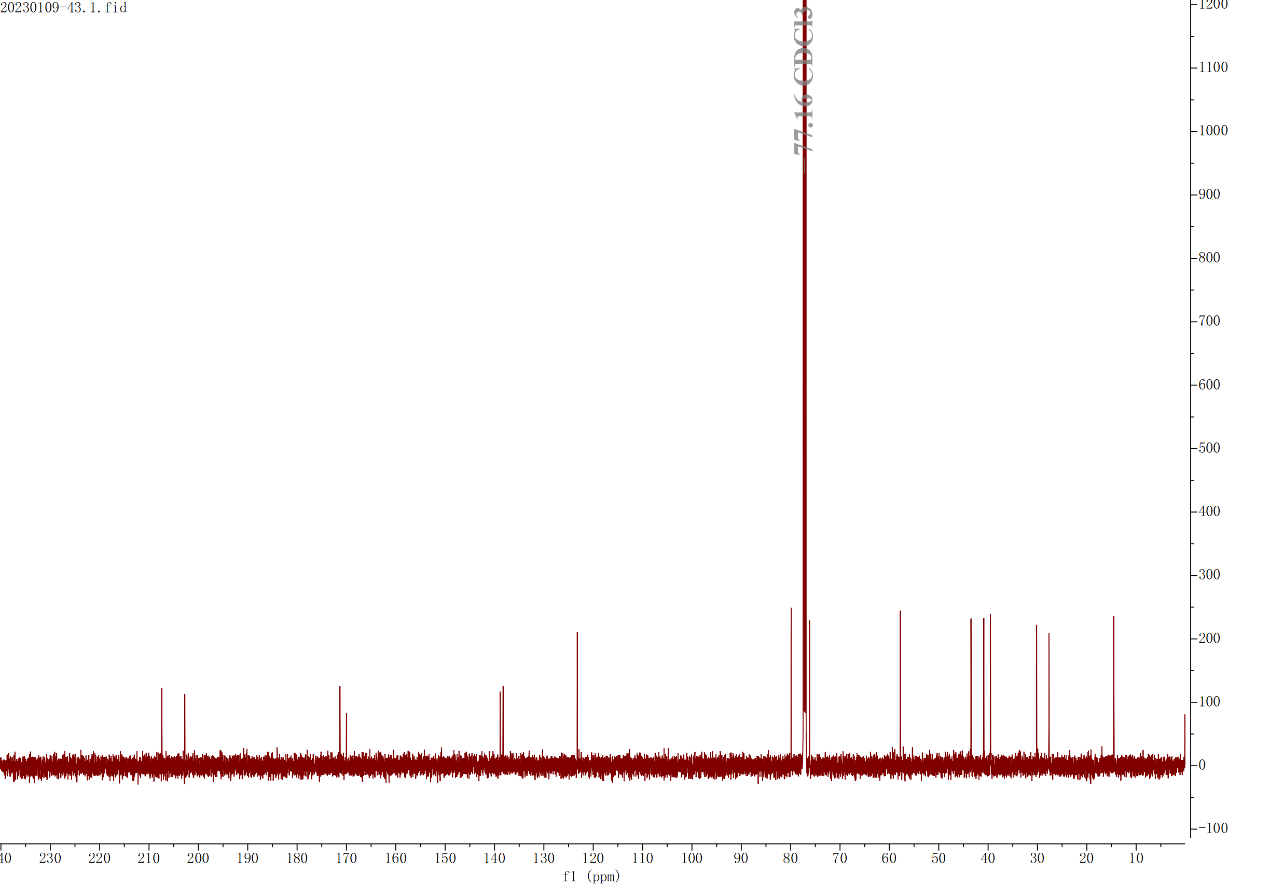


Figure S36. ^13^C-NMR spectrum of compound 10（150 MHz, CDCl_3_）


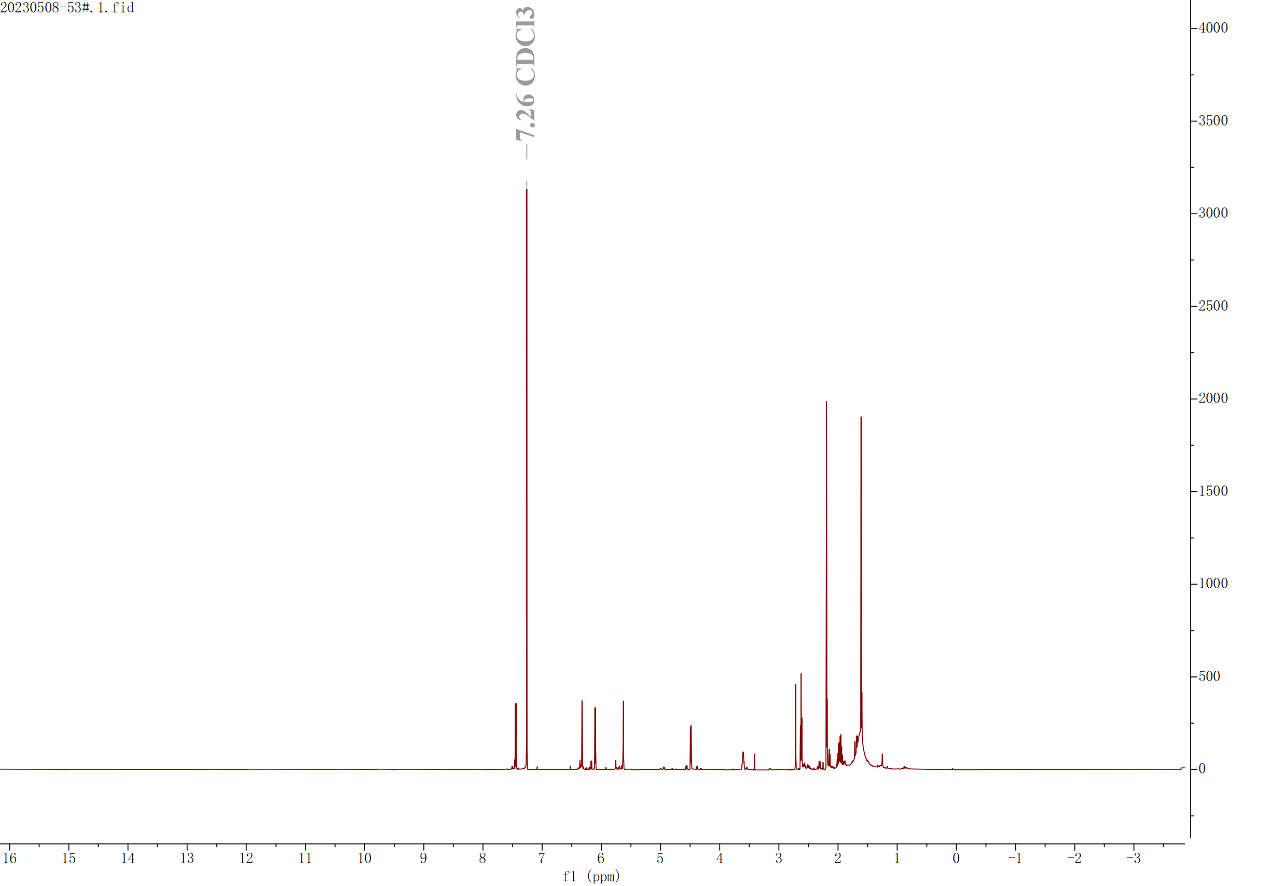


Figure S37. ^1^H-NMR spectrum of compound 11（600 MHz, CDCl_3_）


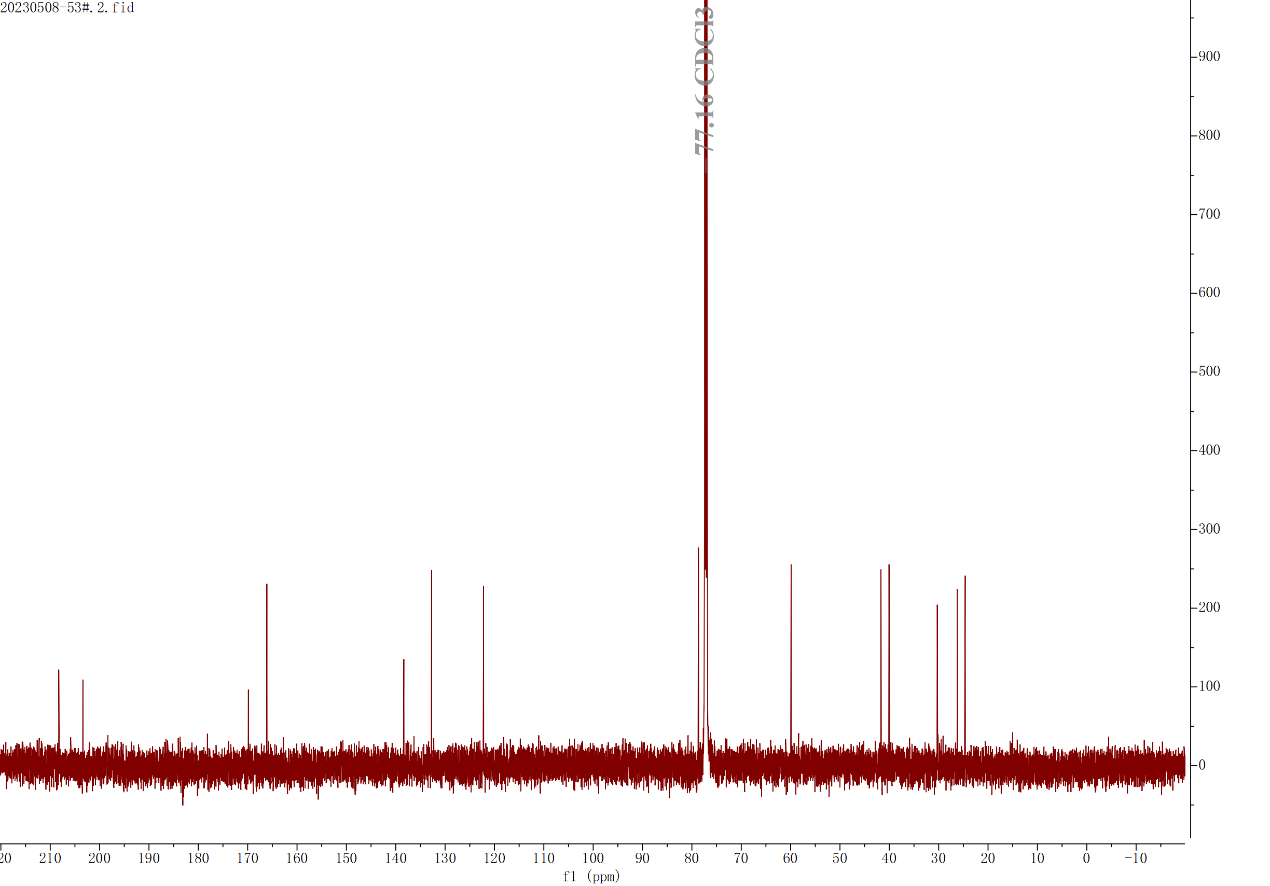


Figure S38. ^13^C-NMR spectrum of compound 11（150 MHz, CDCl_3_）


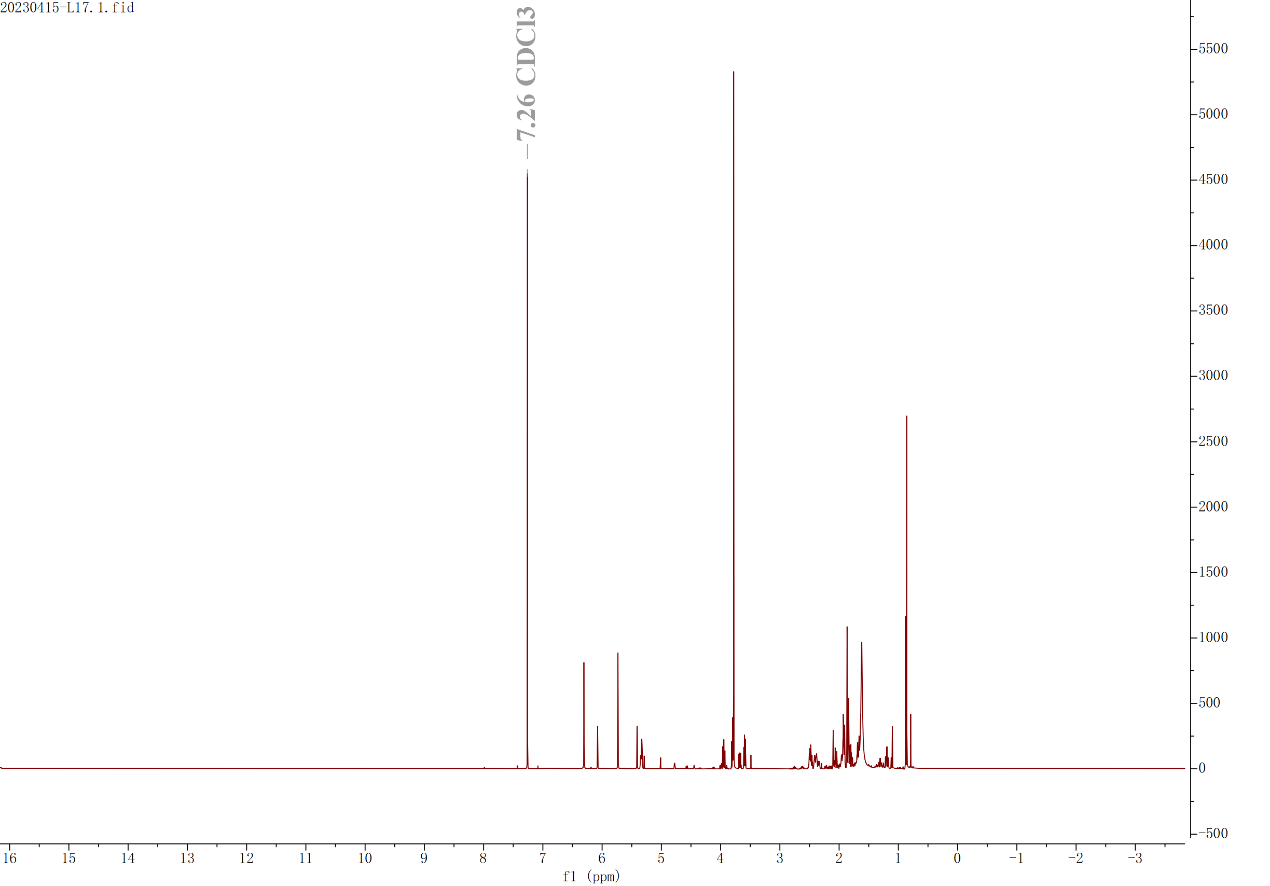


Figure S39. ^1^H-NMR spectrum of compound 12（600 MHz, CDCl_3_）


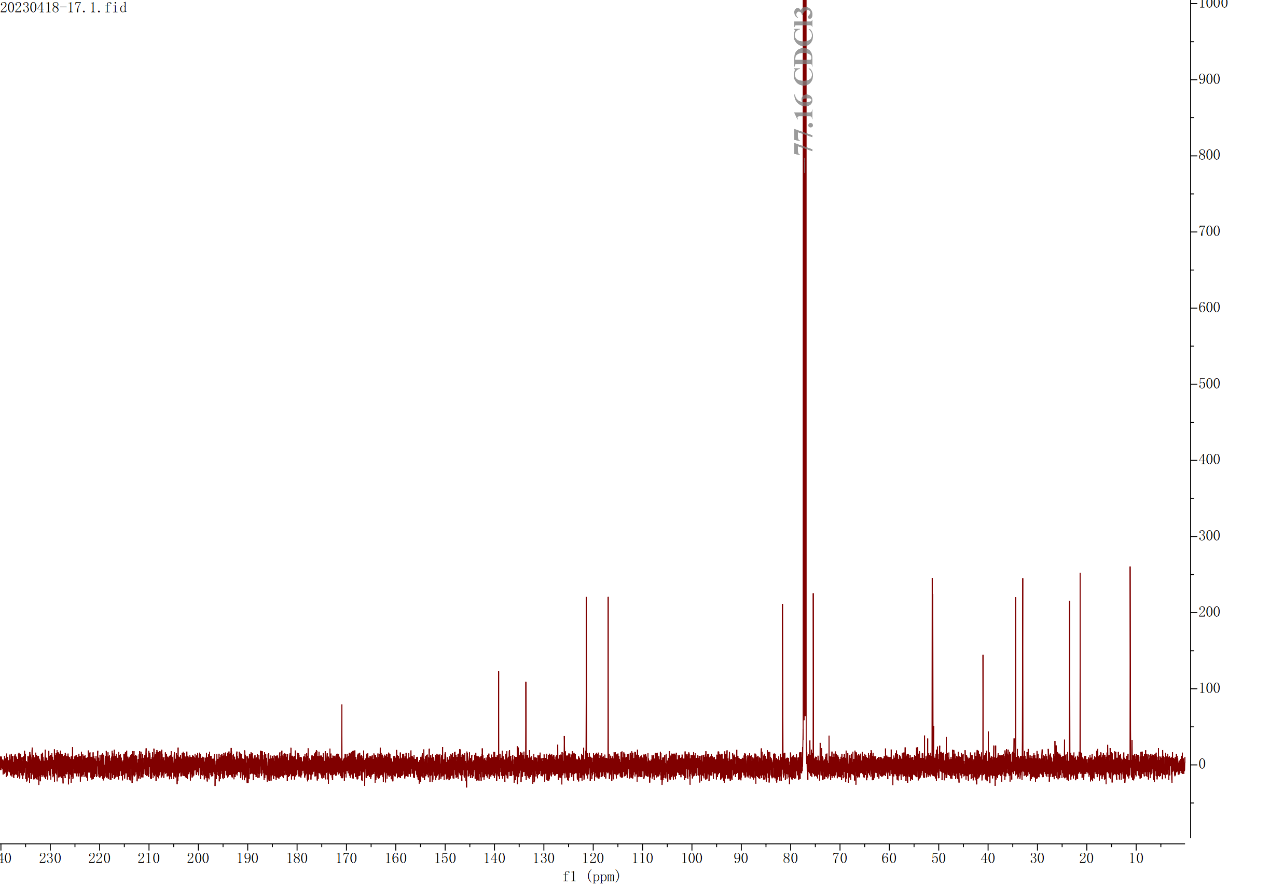


Figure S40. ^13^C-NMR spectrum of compound 12（150 MHz, CDCl_3_）


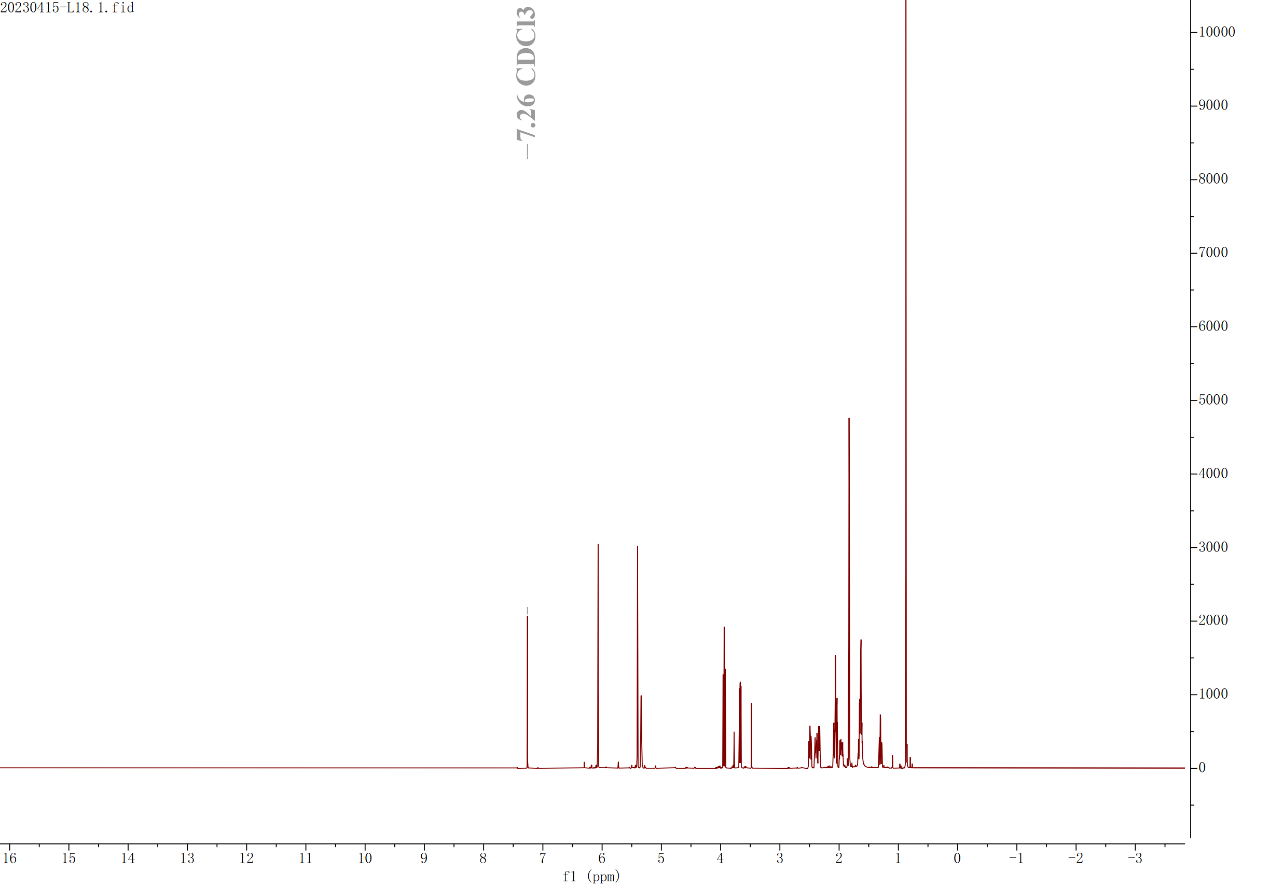


Figure S41. ^1^H-NMR spectrum of compound 13（600 MHz, CDCl_3_）


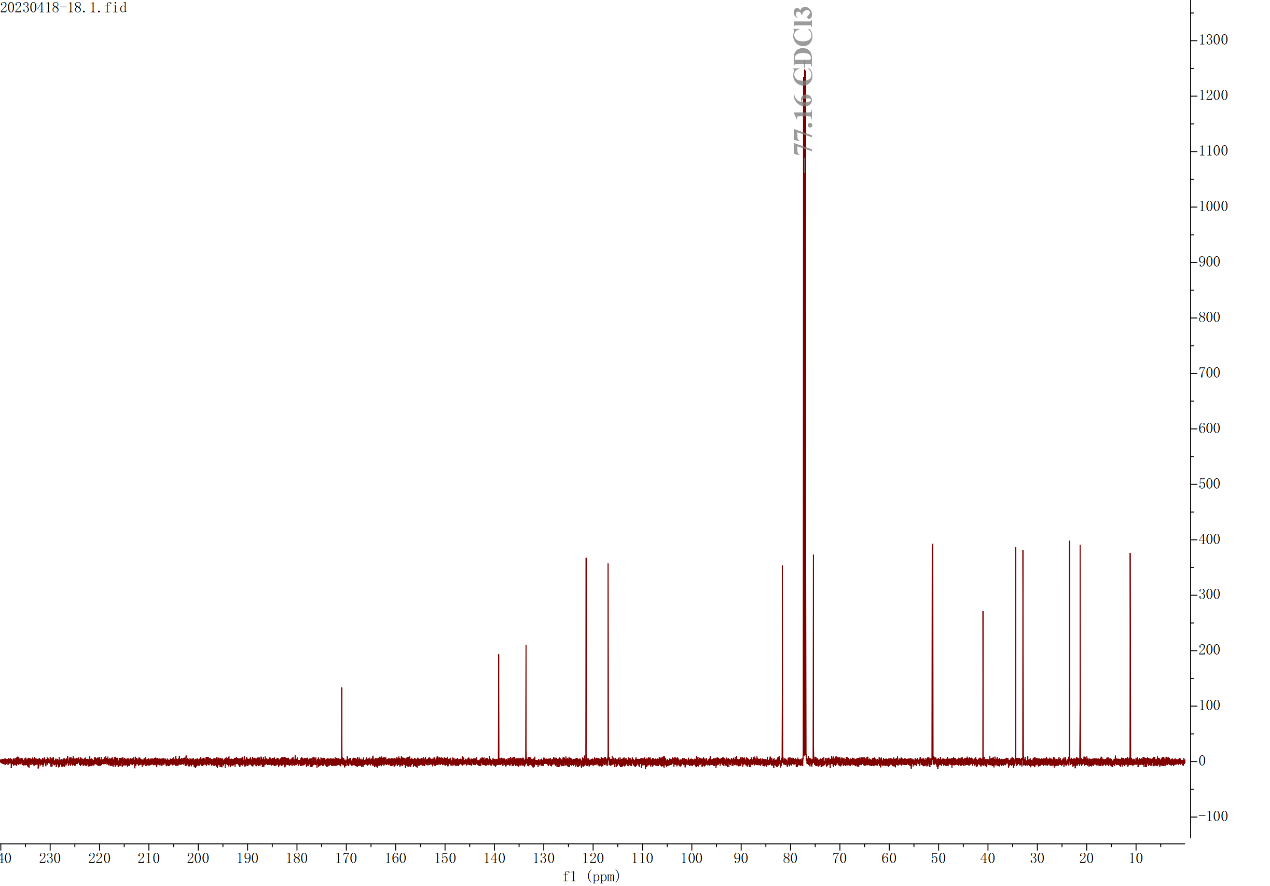


Figure S42. ^13^C-NMR spectrum of compound 13（150 MHz, CDCl_3_）


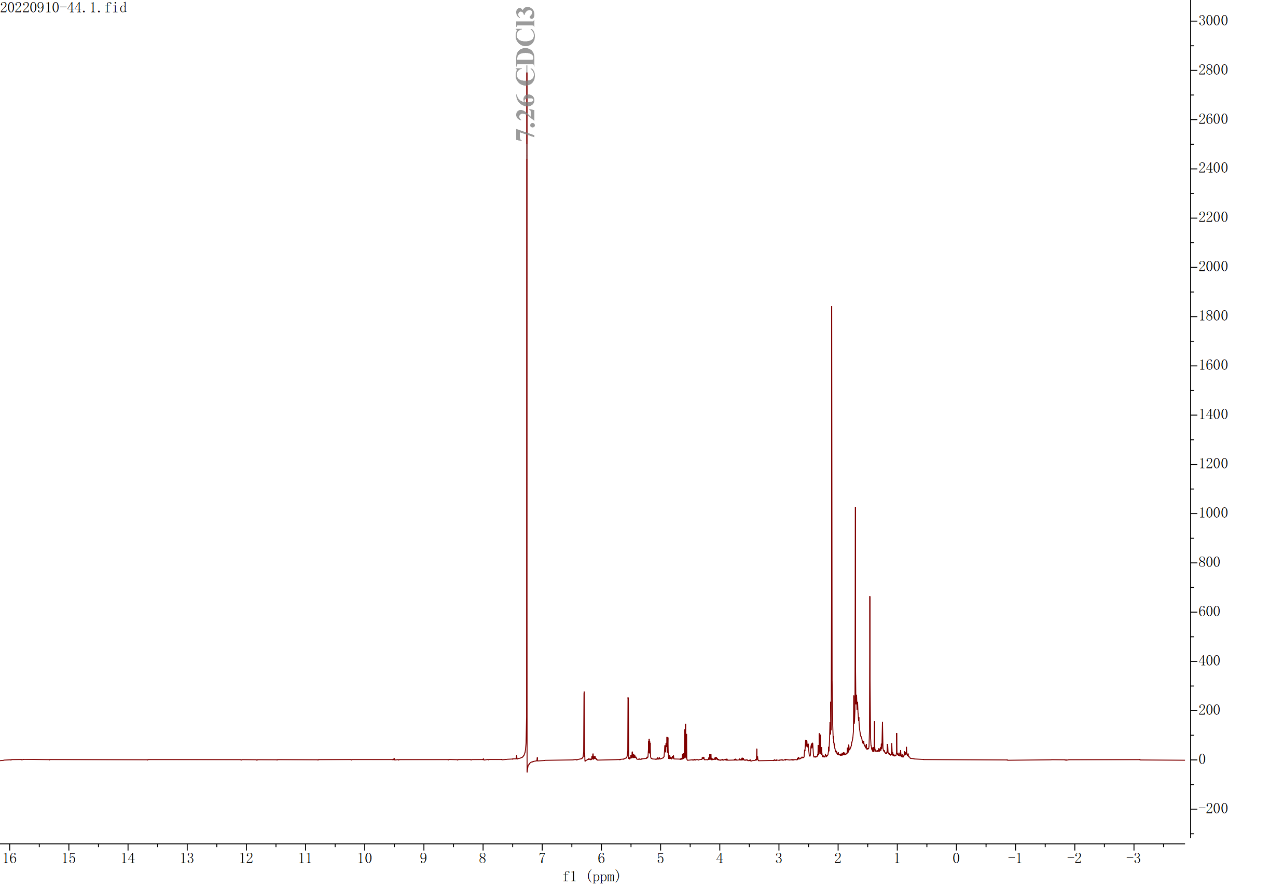


Figure S43. ^1^H-NMR spectrum of compound 14（600 MHz, CDCl_3_）


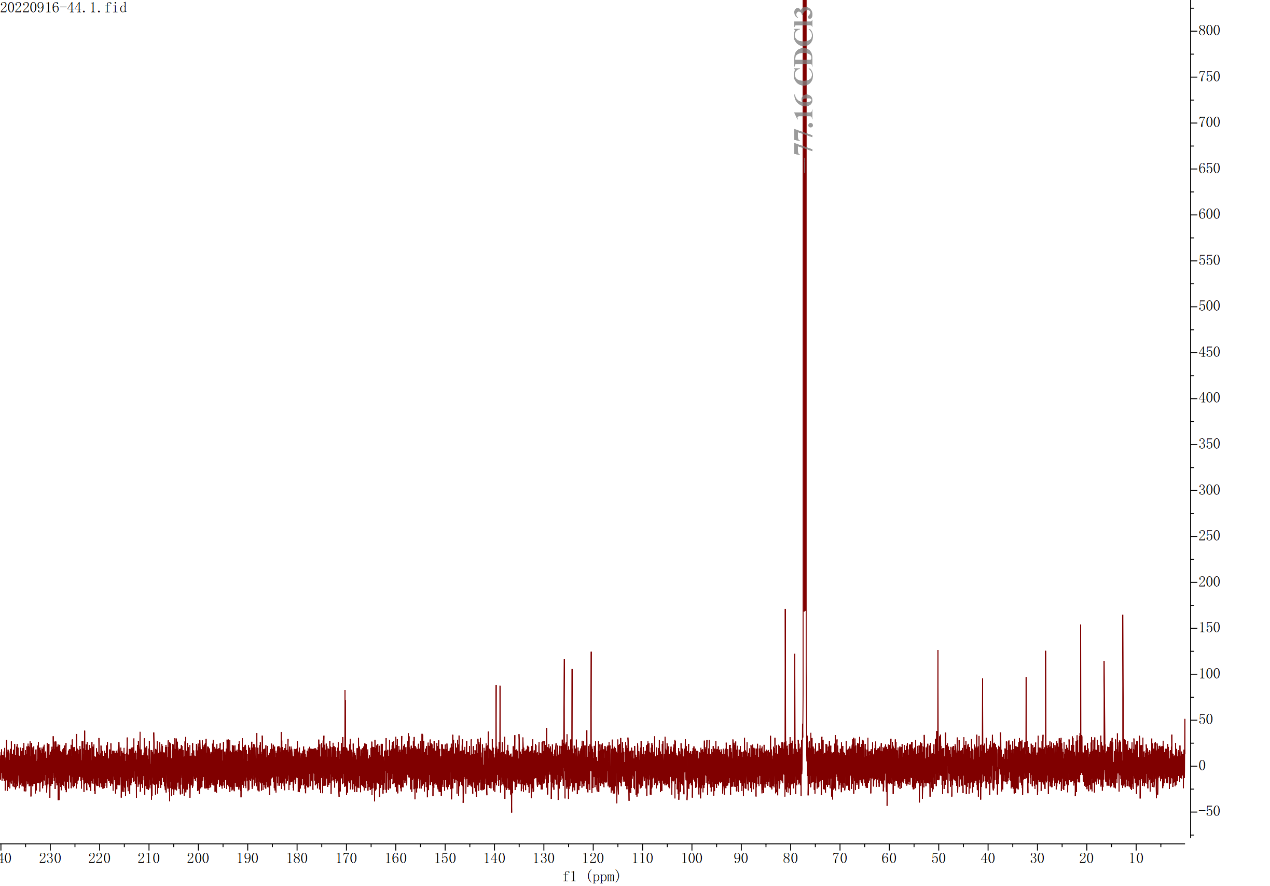


Figure S44. ^13^C-NMR spectrum of compound 14（150 MHz, CDCl_3_）


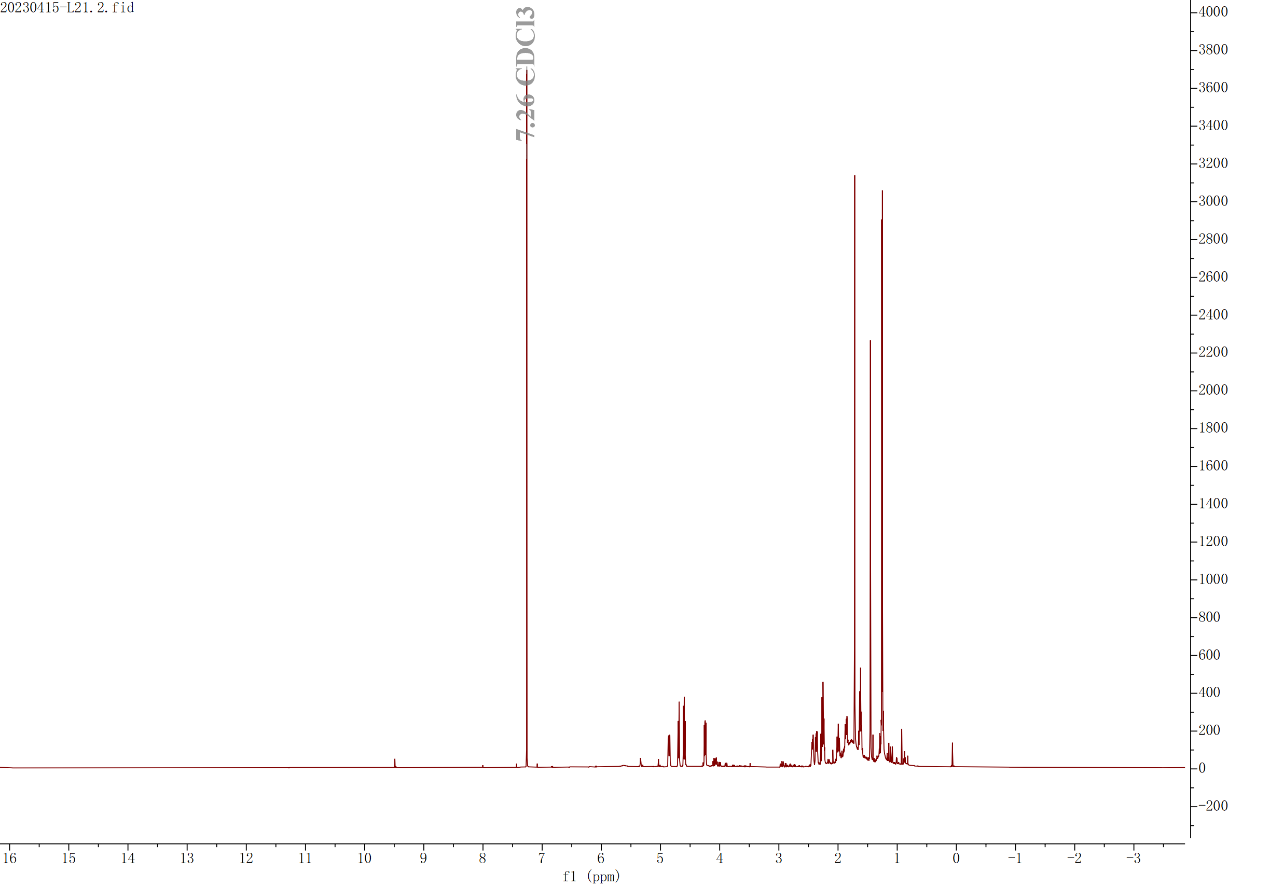


Figure S45. ^1^H-NMR spectrum of compound 15（600 MHz, CDCl_3_）


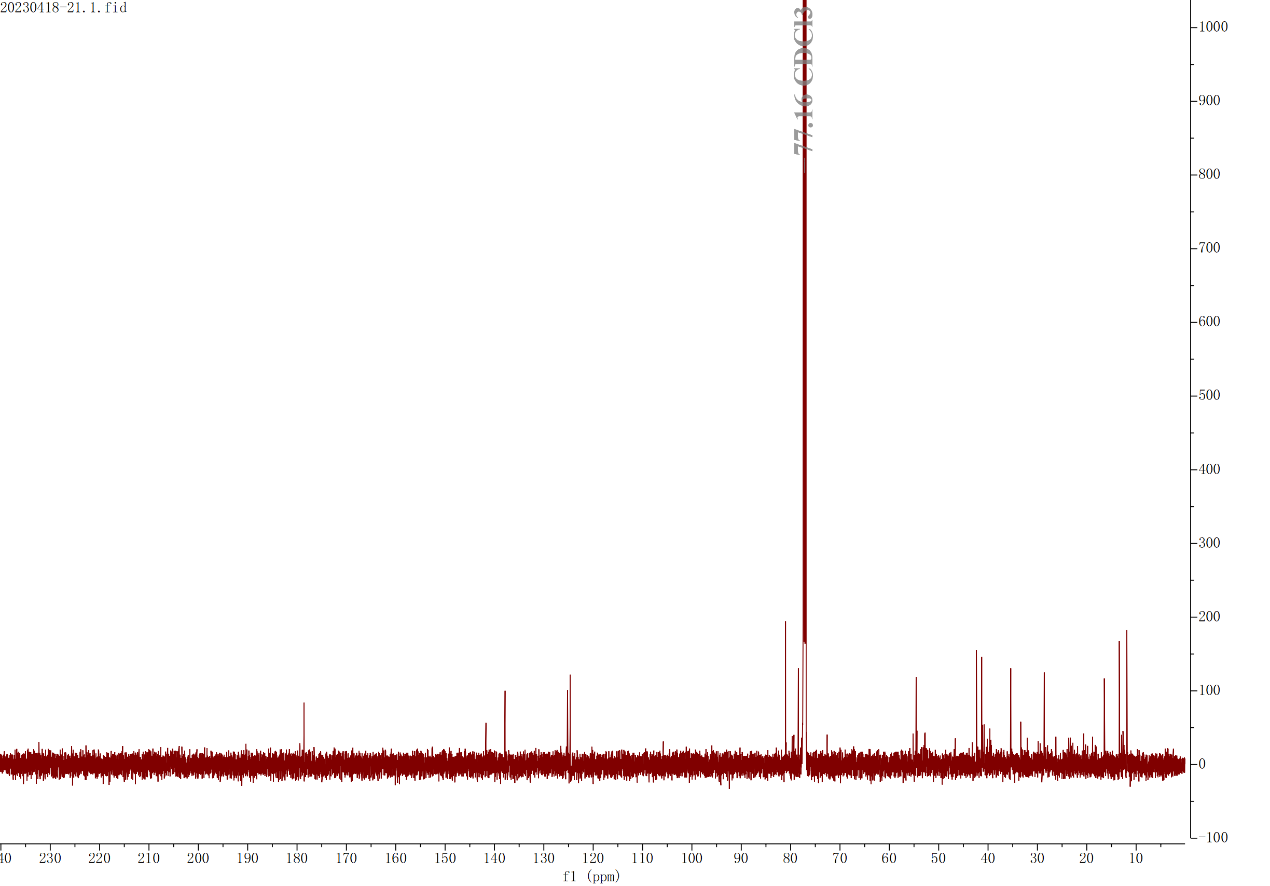


Figure S46. ^13^C-NMR spectrum of compound 15（150 MHz, CDCl_3_）


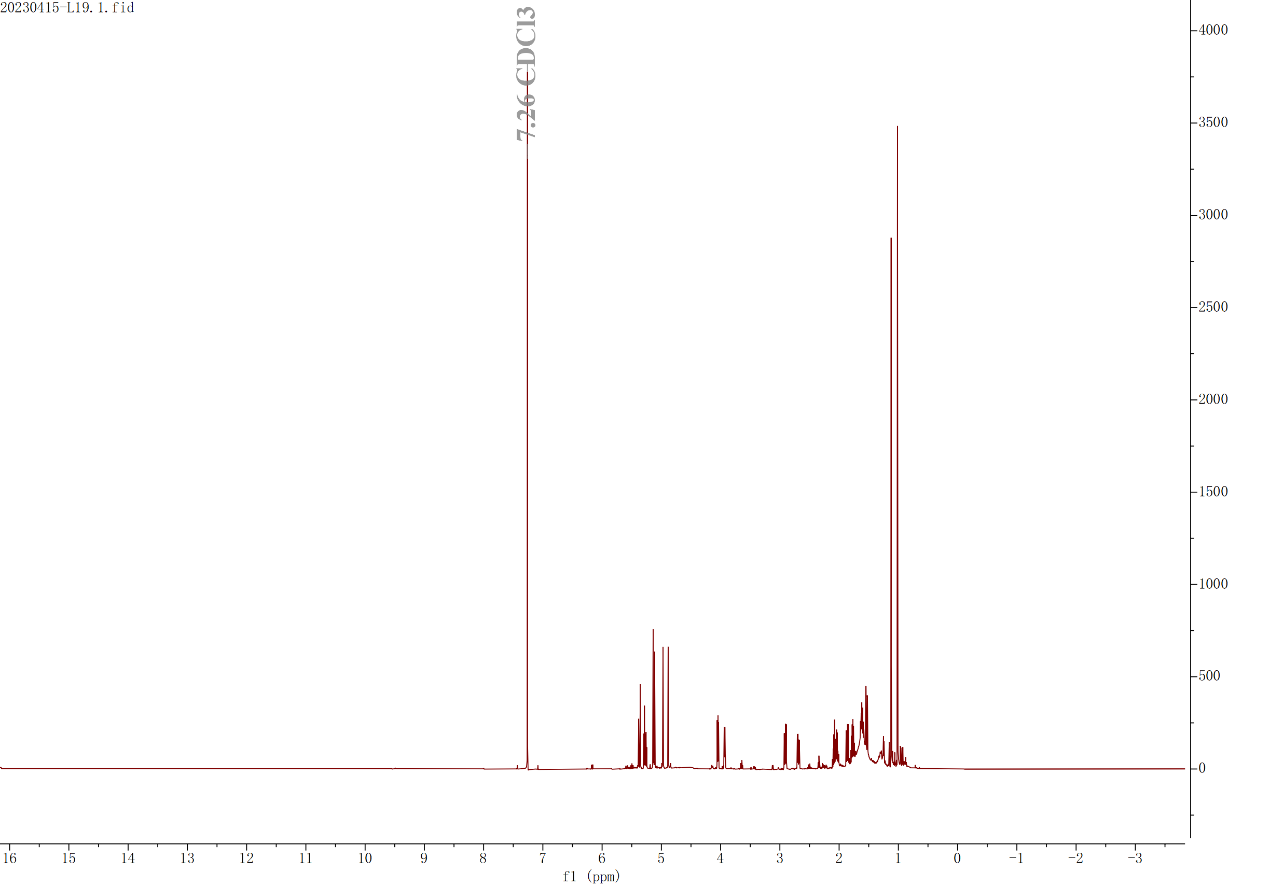


Figure S47. ^1^H-NMR spectrum of compound 16（600 MHz, CDCl_3_）


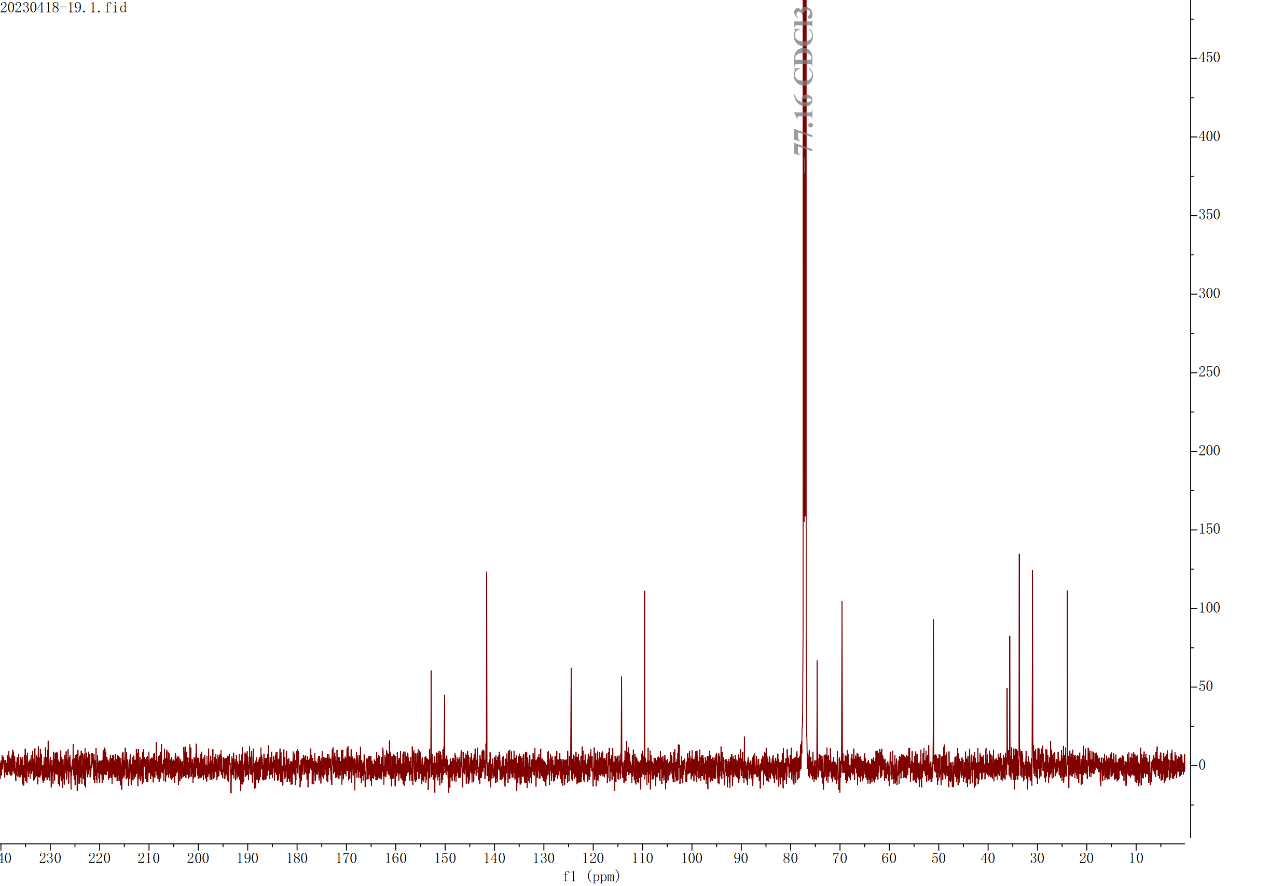


Figure S48. ^13^C-NMR spectrum of compound 16（150 MHz, CDCl_3_）


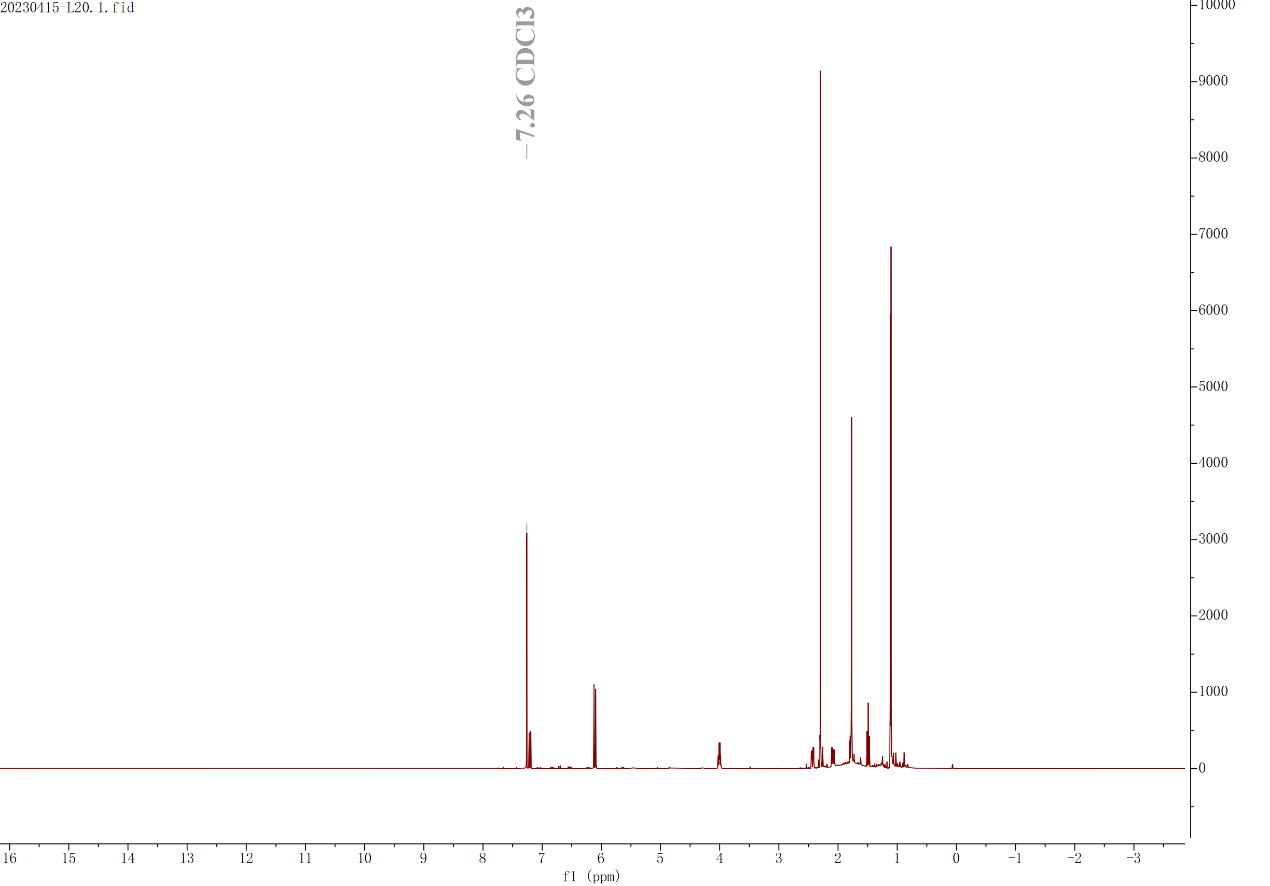


Figure S49. ^1^H-NMR spectrum of compound 17（600 MHz, CDCl_3_）


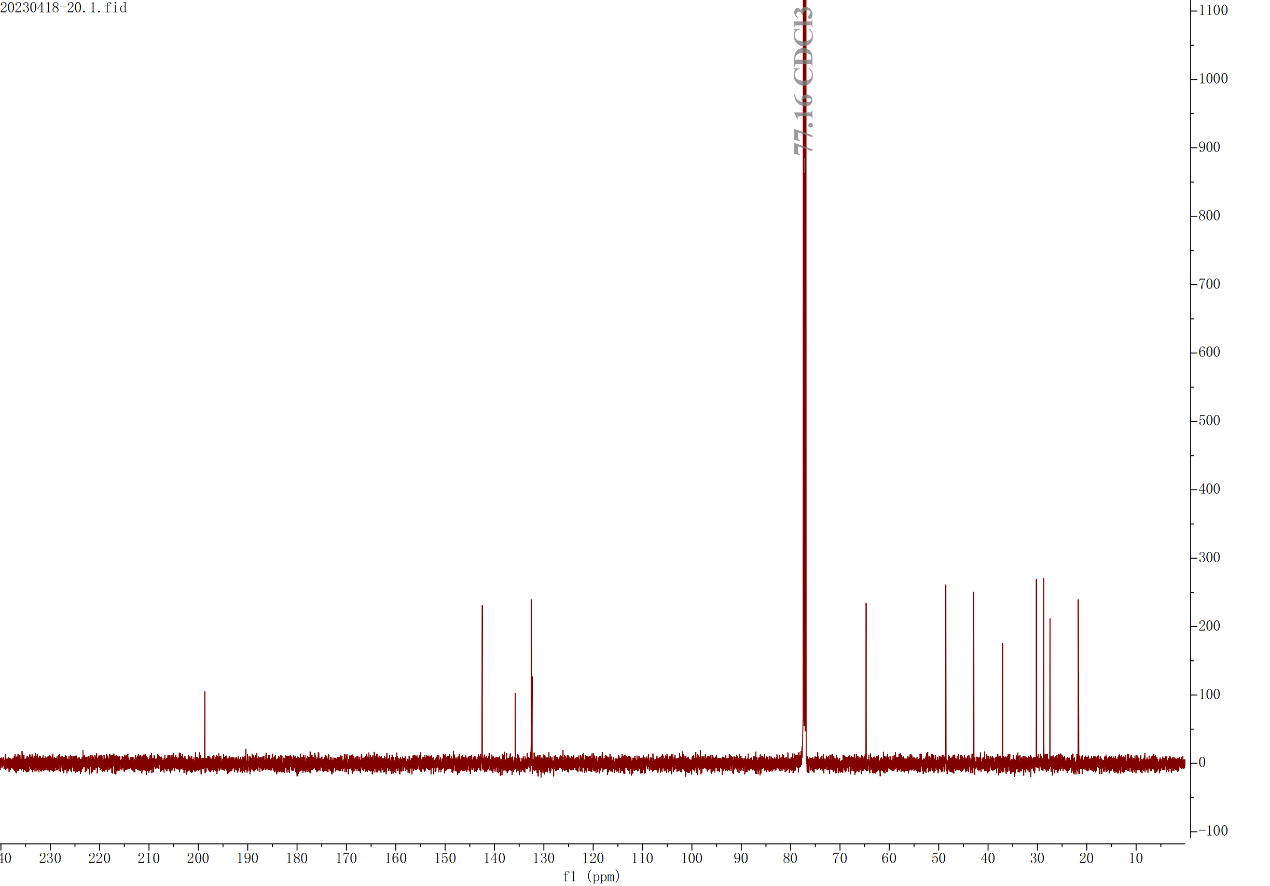


Figure S50. ^13^C-NMR spectrum of compound 17（150 MHz, CDCl_3_）


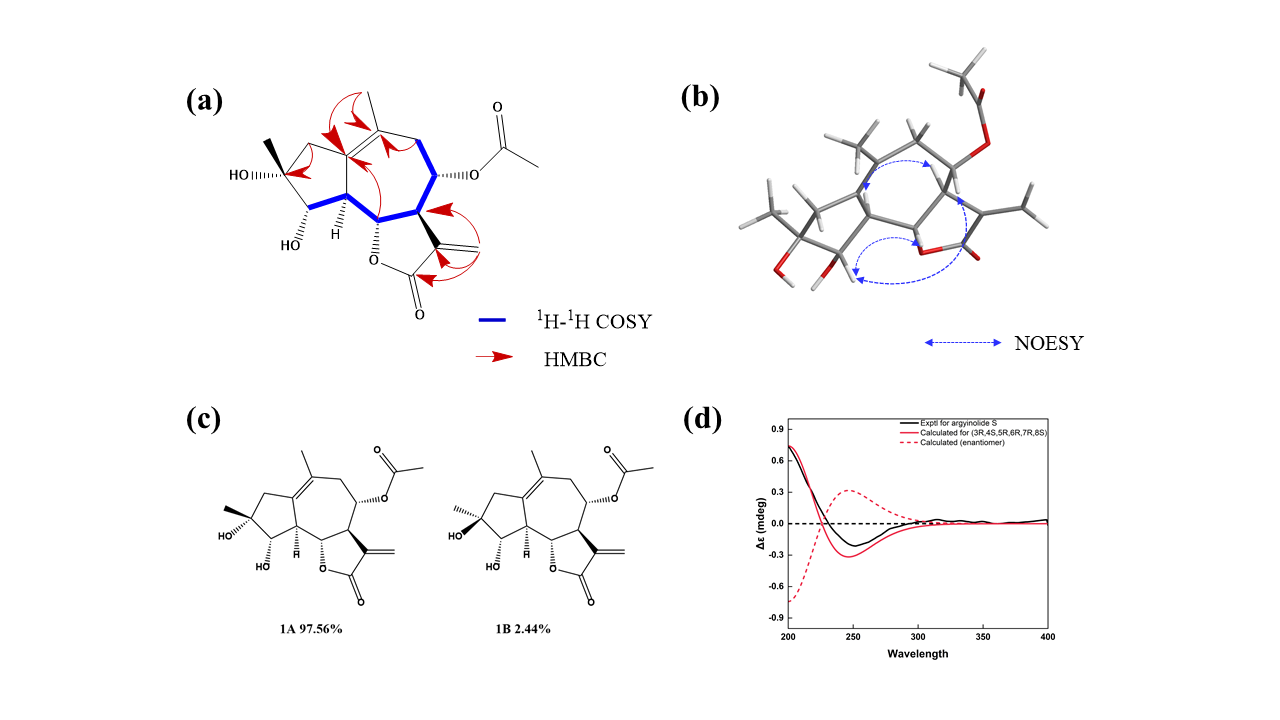


Figure S51. The elucidation process of the structure of Compound 1 (argyinolide S).

(a) Correlation analysis of HMBC and COSY spectra. (b) Key NOESY correlations. (c) DP4+ probability distribution. (d) Experimental and calculated ECD spectra.


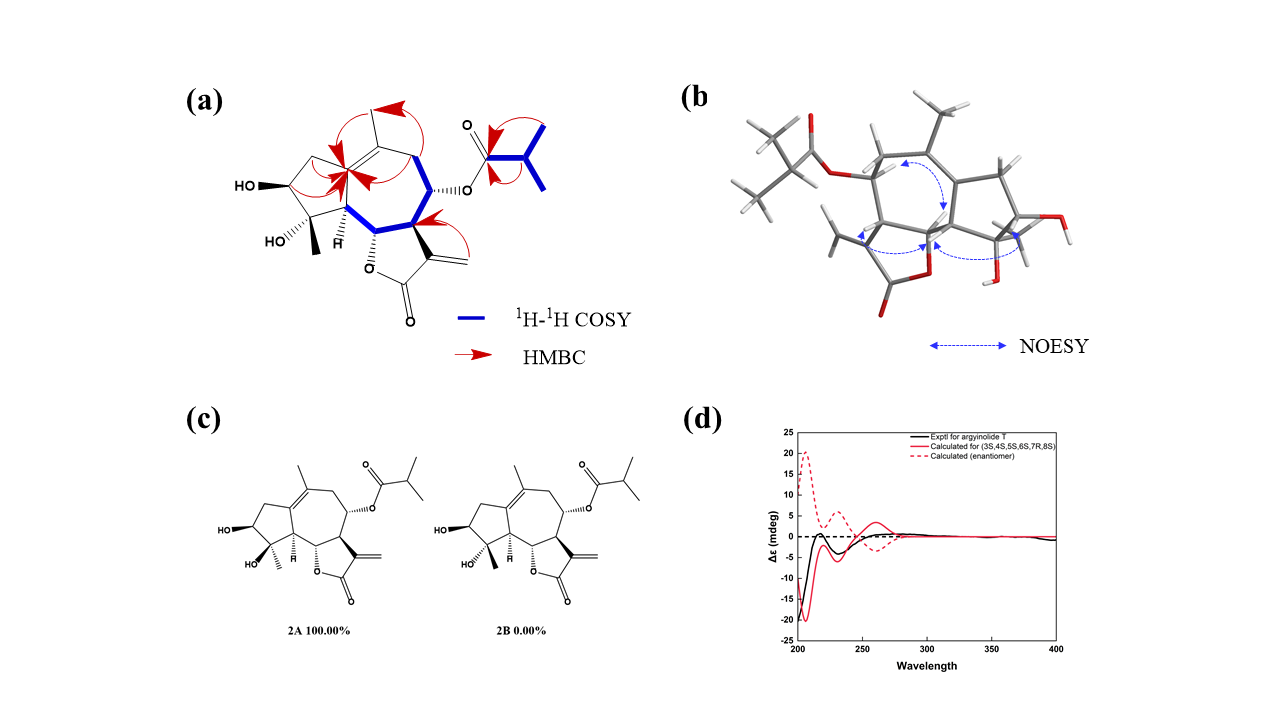


Figure S52. The elucidation process of the structure of Compound 2 (argyinolide T).

(a) Correlation analysis of HMBC and COSY spectra. (b) Key NOESY correlations. (c) DP4+ probability distribution. (d) Experimental and calculated ECD spectra.


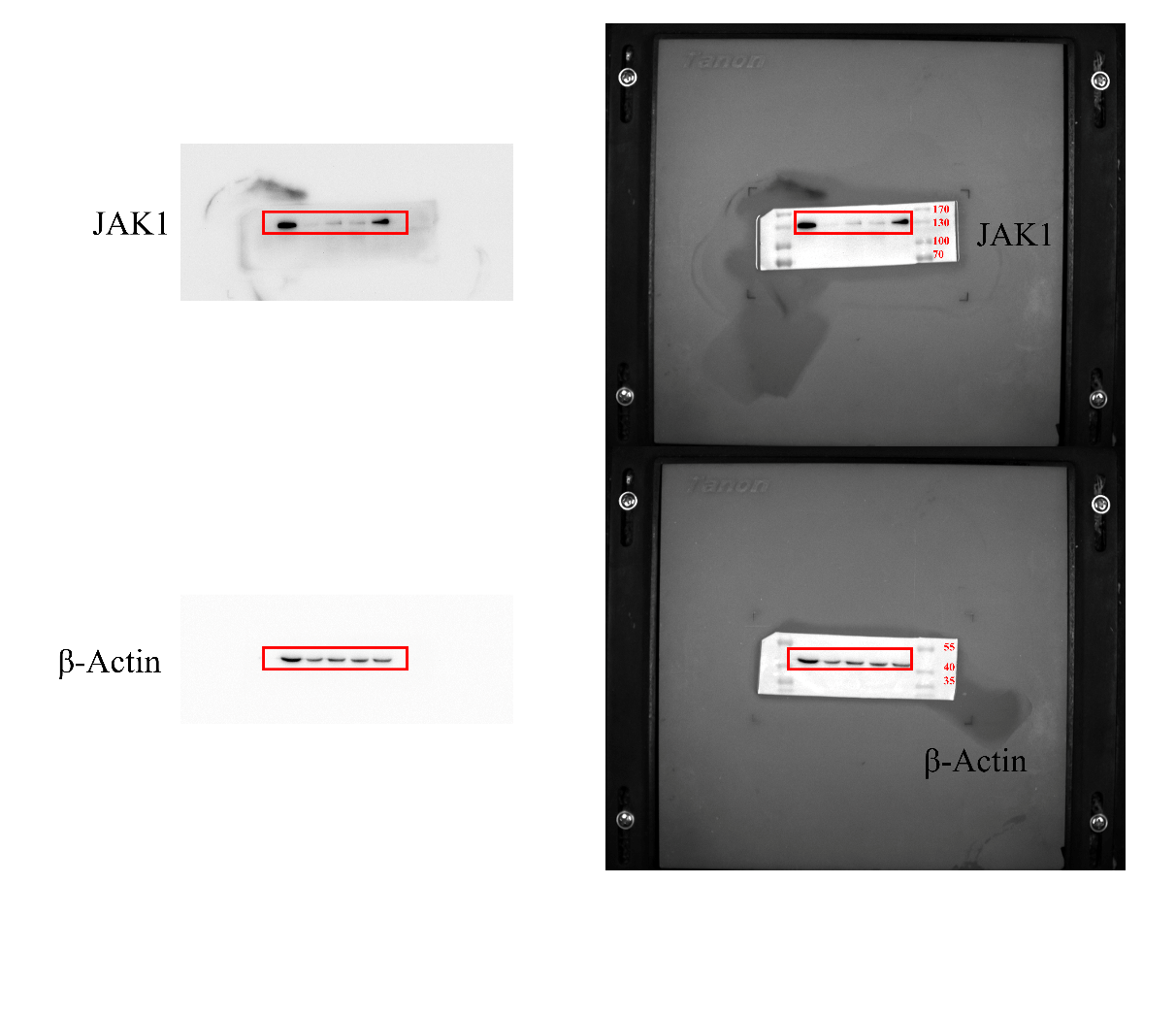


Figure S53. Raw data of the DARTS experiment


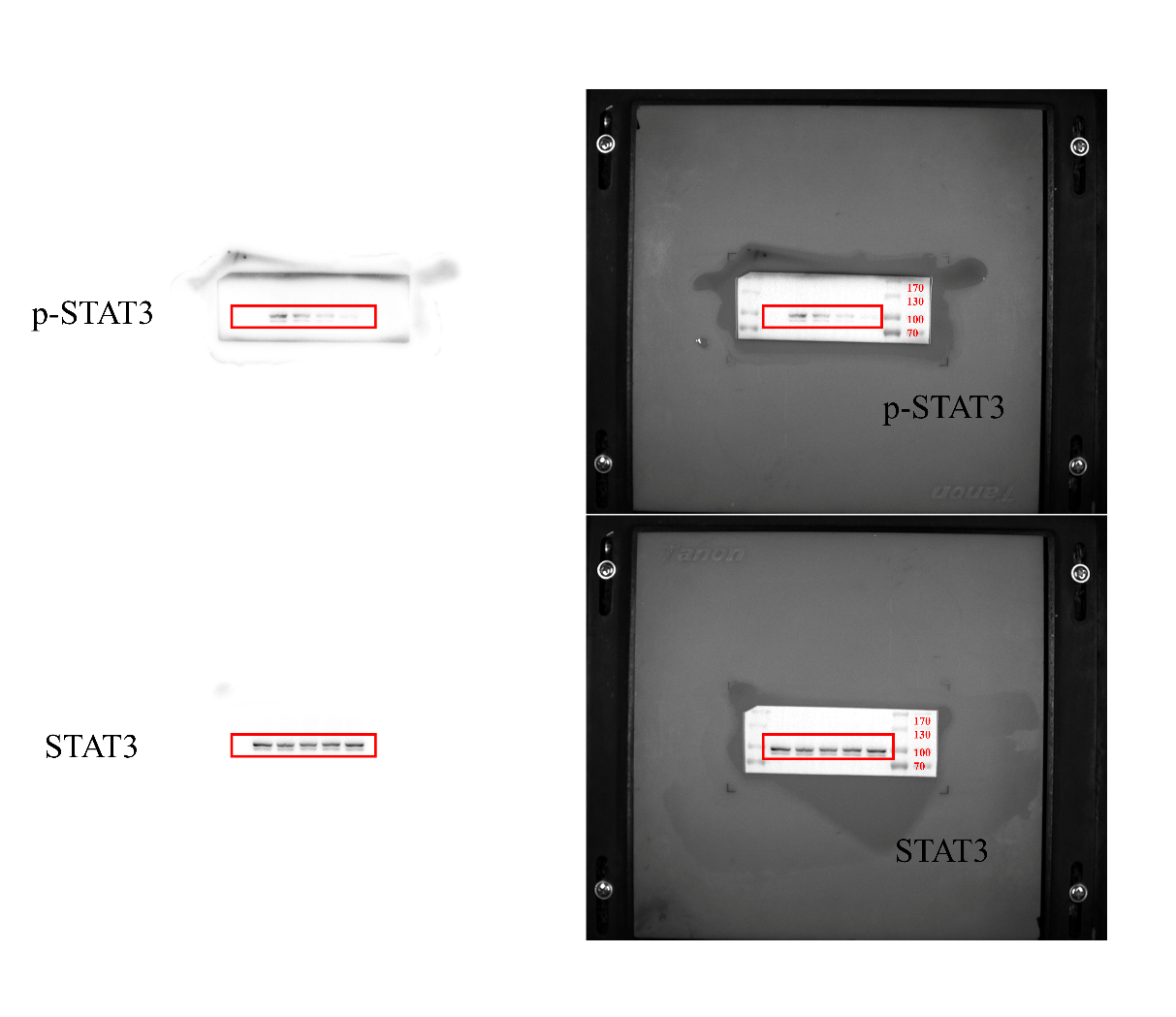


Figure S54. Raw data of the Western Blot experiment

Table S1 The RT-qPCR sequences used in the study

| Genes | Sense(5’-3’) | Antisense(5’-3’) |
| --- | --- | --- |
| IL-1β | TGCCACCTTTTGACAGTGATG | TGATGTGCTGCTGCGAGATT |
| IL-6 | CTCCCAACAGACCTGTCTATAC | CCATTGCACAACTCTTTTCTCA |
| TNFα | AGGGTCTGGGCCATAGAACT | CCACCACGCTCTTCTGTCTAC |
| β-actin | GGCTGTATTCCCCTCCATCG | CCAGTTGGTAACAATGCCATGT |

Table S2 The antibodies used in the study

| Protein | CAT | Manufacturer |
| --- | --- | --- |
| JAK1 | T57173 | Abmart |
| STAT3 | 9139 | CST |
| p-STAT3 | 9145 | CST |
| β-actin | R23613 | Zenbioscience |
