# Supplementary figures and images for: Isolation and anti-neuroinflammation activity of sesquiterpenoids from Artemisia argyi: computational simulation and experimental verification
Source: BMC Complement Med Ther. 2024 Jul 11;24:264. doi: 10.1186/s12906-024-04578-z (PMC11238432; doi:10.1186/s12906-024-04578-z)

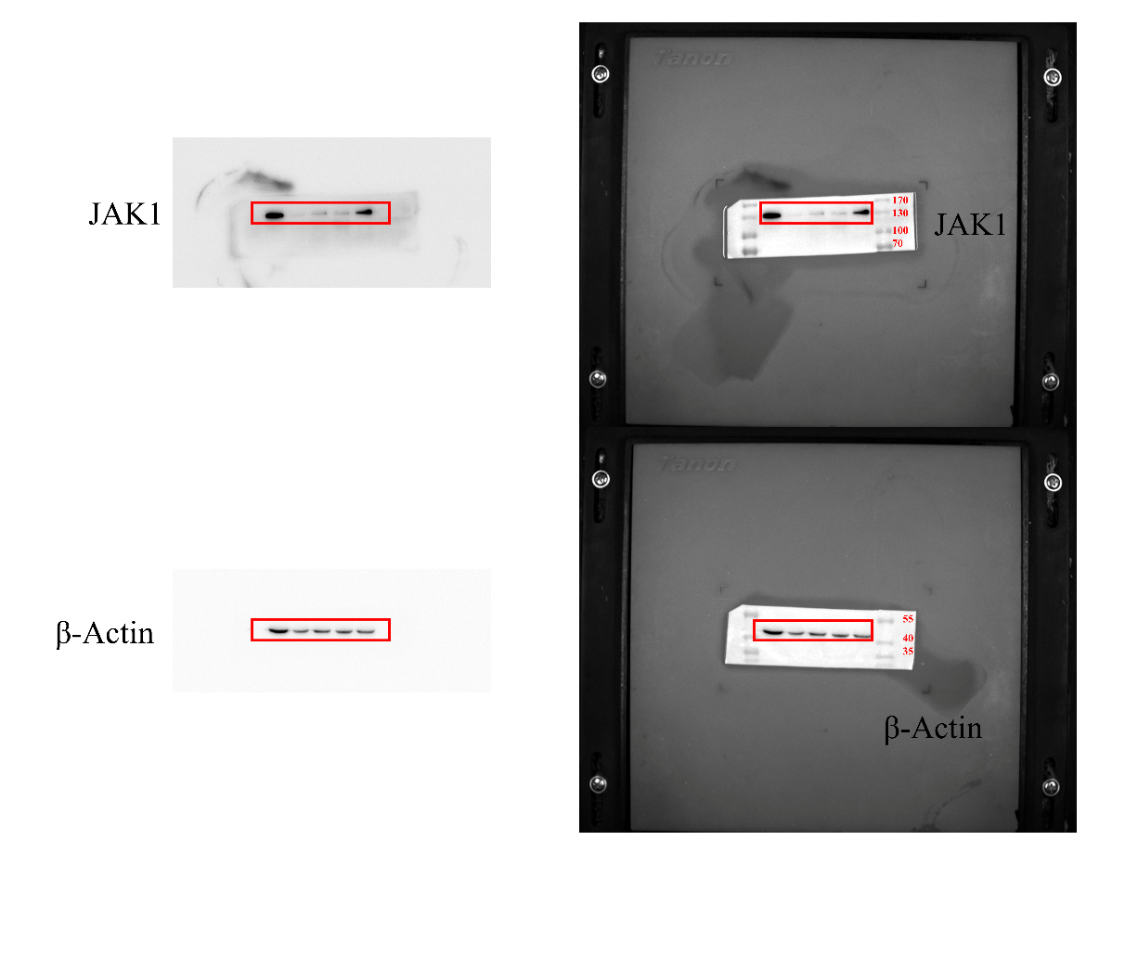


Raw data of the DARTS experiment (Fig. 6a)


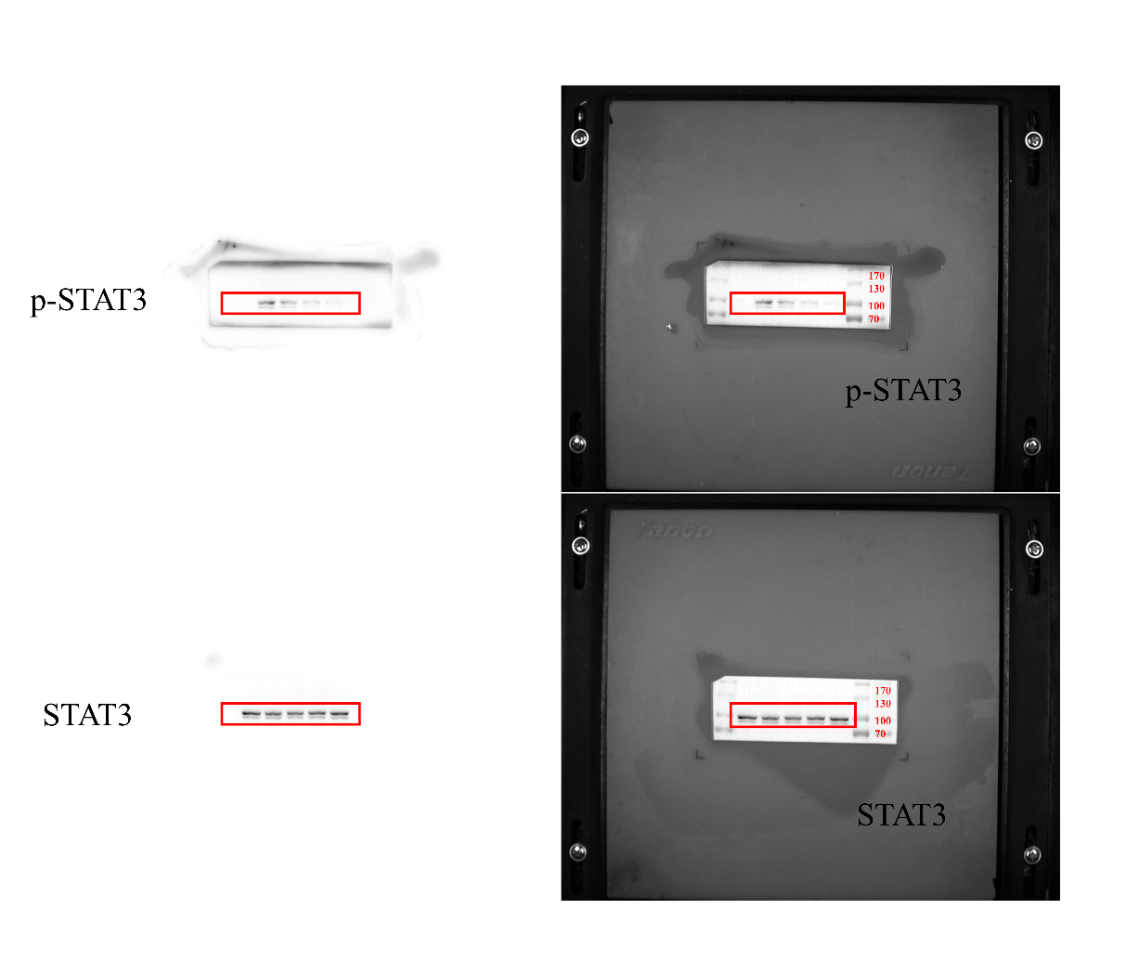


Raw data of the Western Blot experiment (Fig. 6c)

Supplement: Supplementary file 2 — Supplementary Material 2 [file 12906_2024_4578_MOESM2_ESM.docx]
